# Causal impact of gut microbiota on five liver diseases: insights from mendelian randomization and single-cell RNA sequencing

**DOI:** 10.3389/fgene.2024.1362139

**Published:** 2024-11-11

**Authors:** Na Li, Xuanyi Chen, Shuai Xiong, Yuxin Cheng, Jiali Deng, Junli Zhang, Fei Yu, Liyuan Hao, Shenghao Li, Xiaoyu Hu

**Affiliations:** ^1^ Department of Clinical Medicine, Chengdu University of Traditional Chinese Medicine, Chengdu, China; ^2^ Department of Infectious Diseases, Hospital of Chengdu University of Traditional Chinese Medicine, Chengdu, China; ^3^ Acupunctureand Tuina College, Chengdu University of Traditional Chinese Medicine, Chengdu, China; ^4^ Department of Ophthalmology, Key Laboratory of Sichuan Province Ophthalmopathy Prevention and Cure and Visual Function Protection with TCM, Chengdu, Sichuan, China; ^5^ Department of Infectious Diseases, Jiangsu Province Hospital of Chinese Medicine, Affiliated Hospital of Nanjing University of Chinese Medicine, Nanjing, Jiangsu, China

**Keywords:** gut microbiota, five liver diseases, Prevotella, Mendelian randomization, SNPs

## Abstract

**Background:**

Liver disease is among the top ten causes of death globally. With studies suggesting a link between gut microbiota (GM) and liver disease.

**Method:**

We selected summary statistics data from the largest available whole-genome association study (n = 13,266) of GM by the MiBioGen consortium as the exposure, and obtained liver disease-related data from IEU Open GWAS and The NHGRI-EBI GWAS Catalog. A two-sample Mendelian Randomization (MR) analysis employing various methods, to establish the causal relationship between GM and five liver diseases. Meanwhile, single-cell RNA sequencing data were used to examine Prevotella-related genes expression under healthy and disease liver.

**Results:**

The IVW analysis indicate a causal relationship between GM and liver diseases, with Prevotella exhibiting a protective effect in all five liver diseases: Alcoholic liver disease (OR:0.81,95% confidence interval:0.66-1.00,*P*_IVW_ = 0.0494); Cirrhosis (OR: 0.85,95% confidence interval: 0.73-0.99,*P*_IVW_ = 0.0397); Hepatic failure, not elsewhere classified (OR:0.60,95% confidence interval:0.37-0.95,*P*_IVW_ = 0.0305); Benign neoplasm:Liver (OR:0.39,95% confidence interval:0.2-0.75,*P*_IVW_ = 0.0046); Malignant neoplasm of liver, primary (OR:0.41, 95% confidence interval:0.18-0.93,*P*_IVW_ = 0.0334). The single-cell results suggest differential expression of Prevotella-related genes between liver disease patients and healthy individuals.

**Conclusion:**

Our MR results show a causal relationship between the GM and liver disease. Prevotella displays a notable protective effect. This finding may enhance the precision of GM-based therapies and offer new insights for clinical research.

## 1 Introduction

As a vital organ in the human body, the liver plays a crucial role in various biological processes, including metabolism and immunity (Trefts et al., 2017). Liver disease refers to pathological changes caused by various internal and external factors, which significantly impair normal liver function. Factors such as alcohol (Bajaj, 2019), viruses (Liang, 2009), and malnutrition (Mandato et al., 2017) can all lead to liver diseases. Liver disease is one of the ten leading causes of death worldwide (World Health Organization, 2018), resulting in up to 2 million deaths annually due to liver-related complications (Asrani et al., 2019). Research indicates that systemic mucosal immune damage in liver disease patients is closely related to changes in the composition and function of the GM (Bajaj and Khoruts, 2020), This association may be mediated through multiple mechanisms, including impaired intestinal barrier function, dysbiosis of the GM, and increased intestinal permeability, these conditions can lead to the entry of bacterial toxins (such as lipopolysaccharides) into the bloodstream, activating the liver’s immune response, triggering inflammation, exacerbating fibrosis and immune dysregulation, and ultimately driving the progression of liver diseases (Tripathi et al., 2018; Wang et al., 2021). Moreover, certain GM can alter the intestinal barrier to intervene in the progression of liver disease (Plaza-Díaz et al., 2020).

GM is defined as the microbial community that inhabits the human gastrointestinal tract and is a critical factor in regulating host wellbeing. The GM is intricately diverse, including beneficial bacteria that assist in digestion, enhance nutrient uptake, and provide protection from pathogens, along with bacteria that could potentially cause disease. In recent years, research on the GM has grown exponentially (Marchesi et al., 2016), progressively uncovering its regulatory role in various liver diseases. AS research delves deeper, researchers have discovered that disturbances in the gut microbial ecosystem may lead to the exacerbation of multiple liver diseases (Albhaisi et al., 2020). The mutual interaction between the gut and the liver is commonly termed the ‘gut-liver axis’, and it is considered a promising therapeutic approach for managing liver diseases (Wang et al., 2021). Existing research has shown that specific probiotics, such as Bifidobacterium and *Lactobacillus*, can significantly improve the clinical outcomes of various liver diseases, such as alcoholic liver disease (ALD) and non-alcoholic liver disease (NAFLD), by modulating the composition of the GM, enhancing intestinal barrier functions, and reducing inflammatory responses (Hizo and Rampelotto, 2023; Wang et al., 2020).

Currently, an increasing number of studies have identified the GM as a potential target for the treatment of liver diseases (Bajaj et al., 2022). For instance, fecal microbiota transplantation (FMT) has been used to treat alcoholic liver disease and cirrhosis, primarily by restoring the balance of the GM to improve liver function (Boicean et al., 2023). The use of probiotics has also been proven effective for various liver diseases, including ALD (Bajaj, 2019),NAFLD (Vallianou et al., 2021), cirrhosis (Hatton and Shawcross, 2019), and hepatic encephalopathy (Bajaj et al., 2017). These effects are similarly linked to the restoration of GM balance. Thus, Selecting beneficial gut microbes and optimizing the protocols for FMT and probiotics could significantly improve clinical outcomes. However, due to the complexity of GM and its varying roles in different liver diseases, careful selection of gut microbial members and protocol optimization are necessary to maximize clinical benefits. However, it presents a new challenge: what constitutes a “healthy” microbiome that is advantageous for liver diseases? In light of this, establishing a causal relationship between the gut and liver diseases is crucial. This not only aids in revealing the pathogenic mechanisms by which dysbiosis of the GM leads to liver diseases but also provides a basis for precise GM-targeted interventions. However, research in this area is still lacking, and there is an urgent need to explore the causal links between GM and liver diseases. This would enable the identification of ‘liver-beneficial’ microbial communities, thus offering more effective guidance for clinical treatments.

While randomized controlled trials (RCTs) are the gold standard for establishing causality, the complexity of the GM and the influence of host genetics make such trials challenging in this field (Goodrich et al., 2016). The emergence of Mendelian randomization (MR) has made the regulatory role of the GM in diseases more visual. MR, as a statistical method, utilizes genetic variants associated with the exposure as instrumental variables (IVs) and then assesses the association between IVs and outcomes, elucidating causal relationships between exposure and outcomes (Emdin et al., 2017). MR is immune to the influence of confounding factors or reverse causality, ensuring the accuracy and reliability of experimental results (Neeland and Kozlitina, 2017). Consequently, it has garnered significant attention from the scientific community. At present, MR has found extensive application in liver diseases such as non-alcoholic fatty liver disease (Li et al., 2023), autoimmune liver diseases (Fu et al., 2023), and cirrhosis (Xiao et al., 2023). However, there are still research gaps when it comes to diseases like ALD, liver failure, and liver cancer. Furthermore, researchers have not conducted a systematic exploration of the potential relationship between the GM and a variety of liver diseases.

To address this gap, this research is based on the principles of scientific reliability and replicability, using MR as the primary method to probe the potential causal connections between the GM and five distinct liver diseases. We take the GM as the exposure and select five common and progressively worsening liver diseases, namely ALD, cirrhosis, liver failure, benign liver tumors, and primary liver malignancies, as the outcomes. We have found a significant causal association between the GM and the aforementioned liver diseases. What’s astonishing is that the particular genus Prevotella has exhibited a protective effect in all these liver conditions. Additionally, our single-cell analysis revealed significant differences in the expression of Prevotella-related genes under various liver states. This study employs both MR and single-cell, focusing on the GM perspective, to offer more precise treatment strategies for addressing the socioeconomic burden resulting from liver diseases.

## 2 Method

### 2.1 The hypothesis and design of MR studies

**FIGURE 1:**
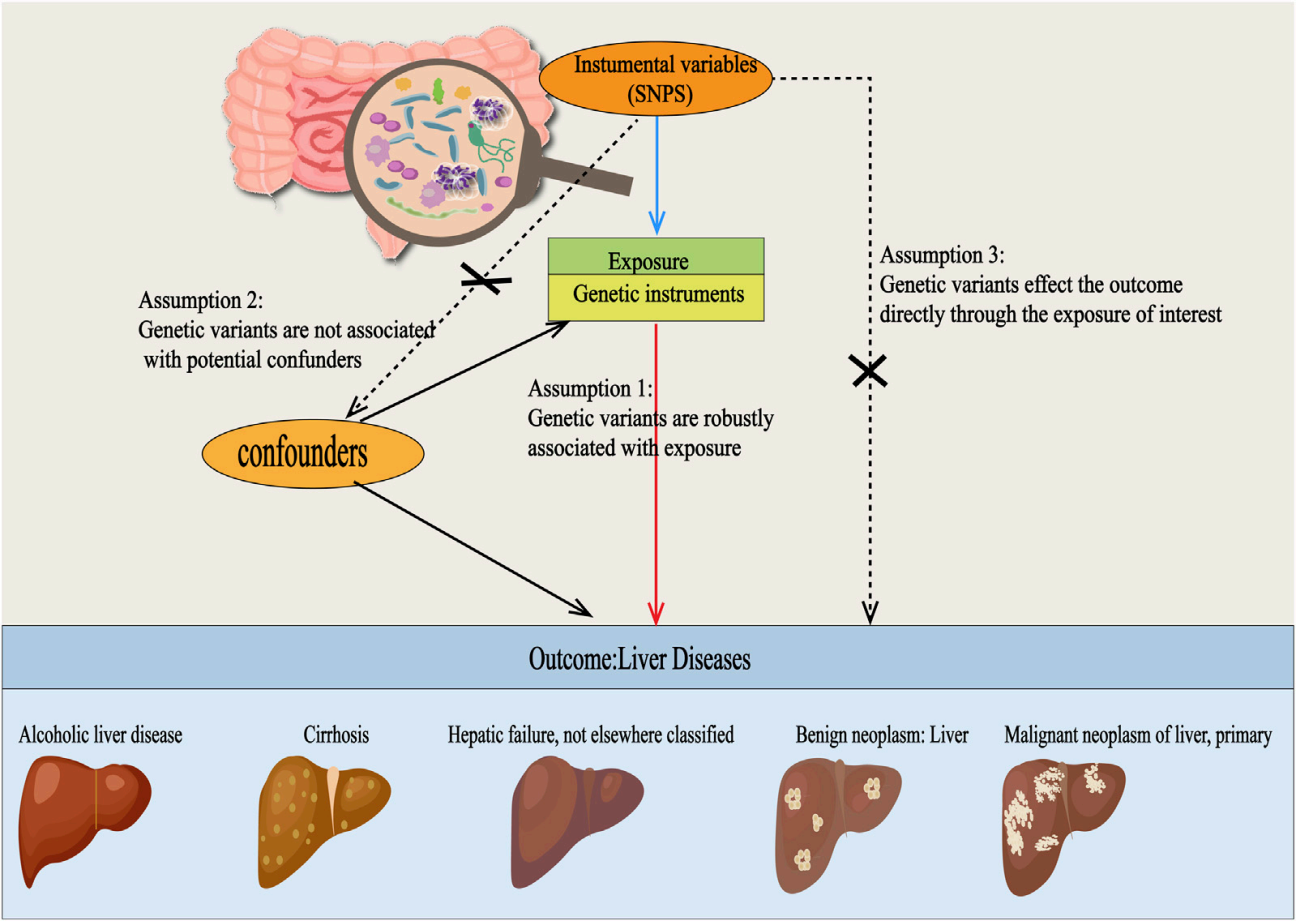
Three Hypotheses Regarding Mendelian Randomization in this Study. SNPs were used as instrumental variables to investigate the causal relationships between gut microbiota and various types of liver diseases, including alcoholic liver disease, cirrhosis, liver failure, benign liver tumors, and primary liver malignancies. For this approach to be valid, three core assumptions must be met: (1) Relevance assumption: the SNPs must be strongly associated with the exposure. (2) Independence assumption: the SNPs should be independent of any confounding factors. (3) Exclusion restriction assumption: the SNPs should only influence the outcome through exposure and not via any other pathways.

**FIGURE 2:**
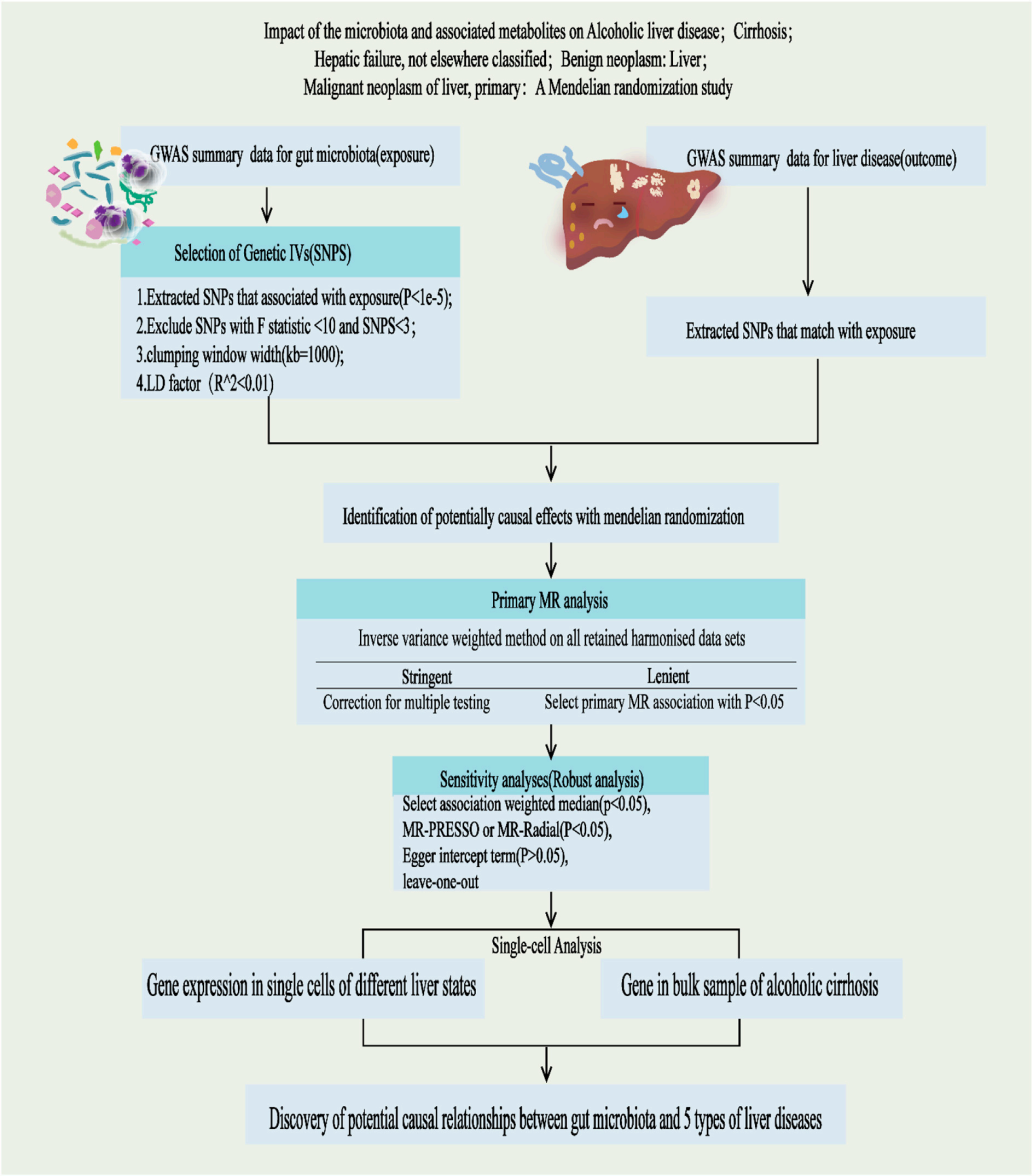
Study design and Operational flow for this Study. IVs, Instrumental Variables; MR, Mendelian Randomization; IVW, inverse variance weighted; MRPRESSO, MR pleiotropy residual sum and outlier; MR-RAPS, MR-Robust Adjusted Profile Score.

This study adheres to the STREGA guidelines (Little et al., 2009) and follows the principles outlined in the Strengthening the Reporting of Observational Studies in Epidemiology (STROBE) guidelines (Skrivankova et al., 2021). The MR method is based on three key assumptions (Davies et al., 2018): ①Association Assumption: SNPs are strongly associated with the exposure factor; ②Independence Assumption: SNPs are independent of confounding factors; ③Exclusivity Assumption: SNPs can only influence the outcome through exposure (Figure 1). We investigated the causal relationship between gut microbiota and five common liver diseases using this framework. GM was treated as the exposure, with liver disease outcomes including liver failure, cirrhosis, alcoholic liver disease (ALD), benign liver tumors, and primary liver malignancies. The sample sizes ranged from 214,056 to 456,348, all drawn from European populations. Subsequently, single-cell sequencing was employed to analyze the gene expression of specific microbial taxa under different liver disease states, followed by a comprehensive analysis and evaluation of the results (flowchart in Figure 2).

### 2.2 Ethical review

The whole-genome association studies (GWAS) included in this research have already been published, and ethical reviews for the respective GWAS studies have been conducted. This study solely utilized summarized data and does not require additional ethical clearance.

### 2.3 Data sources

#### 2.3.1 Exposure data

We obtained human GM-related GWAS datasets as exposure from the international consortium MiBioGen (https://mibiogen.gcc.rug.nl/). This multi-ethnic, large-scale GWAS study encompassed 24 cohorts and obtained 16S rRNA gene sequencing profiles and genotype data from 18,340 participants to investigate the relationship between genetic variations and trans kingdoms. It included a total of 211 GM taxa (9 phyla, 16 classes, 20 orders, 35 families, and 131 genera) (Kurilshikov et al., 2021).

#### 2.3.2 Outcome data

GWAS summary statistics for ALD, cirrhosis, liver failure, and benign neoplasm of the liver were obtained separately from the publicly available IEU Open GWAS (https://gwas.mrcieu.ac.uk/), A complete dataset of primary liver malignancies was obtained from The NHGRI-EBI GWAS Catalog (https://www.ebi.ac.uk/gwas/) (specific information on outcome data is shown in Table 1).

**TABLE 1:** Outcome-related data.

Outcome	id. Outcome	Year	Population	Sample size	N-case	N-control	Number of SNPs
Alcoholic liver disease	finn-b-ALCOLIVER	2021	European	218,792	1,416	217,376	16,380,466
Cirrhosis	ebi-a-GCST90018826	2021	European	347,406	122	347,284	19,079,888
Hepatic failure, not elsewhere classified	finn-b-K11_HEPFAIL	2021	European	214,056	464	213,592	16,380,437
Benign neoplasm: Liver	finn-b-CD2_BENIGN_LIVER	2021	European	218,792	232	218,560	16,380,466
Malignant neoplasm of liver, primary	GCST90041812	2021	European	456,348	128	456,220	11,831,932

#### 2.3.3 Single-cell data

The single-cell RNA sequencing data were obtained from the Single Cell Portal platform (http://singlecell.broadinstitute.org), encompassing a total of 328,783 cells from 65 samples. These samples consist of 38 from healthy livers, 26 from diseased livers, and 1 from an unidentified source. The datasets include GSE185477, GSE125188, GSE156625, GSE192740, GSE115469, and GSE136103 (Fabre et al., 2023).

### 2.4 Selection of instrumental variable

In this study, we excluded 15 microbial taxonomic groups that lacked specific species names (unknown family or genus), and incorporated a total of 196 bacterial taxonomic groups. To ensure the robustness of the data and the accuracy of the results, we conducted a quality check on the SNPs of the GM. Considering the limited number of available SNPs, we selected SNPs related to gut bacterial taxonomic groups with a genome-wide significance threshold of P < 1e-5 as potential IVs. We calculated the F-statistic to assess the issue of weak instrument bias (Pierce et al., 2011).

Additionally, to ensure the effectiveness of the IVs, we excluded IVs with an F-statistic less than 10 (indicating weak IVsand those consisting of fewer than 3 SNPs. Using a clustering process (R^2^ < 0.01, clustering distance = 10,000 kb) to assess the linkage disequilibrium (LD) among the included SNPs, to obtain IVs that meet the criteria for subsequent research on potential causal relationships between exposure and outcomes.

### 2.5 MR analysis

We use the “TwoSampleMR” R package to perform Two-Sample MR analysis investigating the link between exposure and outcomes, our primary method for causal inference is the inverse-variance weighted (IVW). Additionally, we apply the Weighted Median (Bowden et al., 2016), MR-Egger (Bowden et al., 2015), Simple mode, and Weighted mode (Hartwig et al., 2017) as alternative models. Specifically, IVW provides accurate estimates across all IVs and is sensitive to invalid IVs (Xue et al., 2021); MR-Egger is applicable for identifying and correcting pleiotropy, but its estimation precision is relatively lower (Burgess and Thompson, 2017). While the Weighted Median approach can offer a precise estimation, it necessitates that at least 50% of the IVs are valid^26]^; The Simple model provides robust polytropy, but is less powerful than IVW, and the weighted mode is more sensitive to outcomes (Hartwig et al., 2017). We initially apply MR-Egger for a horizontal pleiotropy test, if the p-value is greater than 0.05, it indicates no significant horizontal pleiotropy. MR-PRESS is used to identify and correct horizontal heterogeneity to ensure higher accuracy (Verbanck et al., 2018). We utilized Cochran’s Q test to assess heterogeneity among IVs, and performed a leave-one-out sensitivity analysis to examine the stability of outliers and outcomes. We considered the nominal significance level for MR estimates, meaning that we regarded *p* < 0.05 as having a nominal causal effect. Additionally, we employed the Benjamini-Hochberg (BH) method for FDR correction. Results are considered to show a significant association if both the nominal p-value and the BH-corrected p-value are less than 0.05; results are considered suggestive of an association if the nominal p-value is less than 0.05 but the BH-corrected p-value is greater than 0.05. All analyses were conducted using the R software (version 4.3.1) (website: http://www.rstudio.com/) with the “TwoSampleMR” package.

### 2.6 Mapping SNPs to genes

We utilized the online database SNPnexus (https://www.snp-nexus.org/v4/), a web-based variant annotation tool, to map each queried variant to its nearest gene, which could either be an overlapping gene or one located upstream or downstream. Based on these mappings, we performed a single-cell analysis of genes related to Prevotella.

### 2.7 Single-cell RNA sequencing data analysis

To better understand the role of Prevotella in liver health and disease, we performed a comprehensive single-cell RNA sequencing analysis. Specifically, we explored the heterogeneity of Prevotella across healthy and diseased states through detailed bubble plot analyses, focusing on the gene expression differences of three particular Prevotella taxa (including genus. Prevotella7. id.11182, genus. Prevotella9. id.11183, and genus. Paraprevotella.id.962) across different liver states. Additionally, we selected primary liver samples from two patients with liver cirrhosis and two healthy controls from the aforementioned database, yielding scRNA-seq data for 20,502 cells. We employed Uniform Manifold Approximation and Projection (UMAP) to visualize these high-dimensional scRNA-seq datasets and performed cellular clustering based on UMAP-1 and UMAP-2 dimensions.

## 3 Results

### 3.1 MR results

In this study, we investigated potential causal relationships between 211 gut microbial taxa and alcohol-related liver disease, cirrhosis, liver failure, benign liver tumors, and primary liver malignancies through MR analysis. The key findings are summarized as follows (For specific details, please consult Table 2 and Attachment 1).

**TABLE 2:** Prominent gut microbiota linked to liver disease.

Liver disease (outcome)	Gut microbiota (exposure)	Method	Number of SNPs	β	SE	p-value	Adjusted p	OR	95% CI	Q_pval
Alcoholic liver disease	genus.Lachnospira.id.2004	IVW	6	−0.7687	0.3191	0.0160	0.6069	0.46	(0.25-0.87)	0.3506
	genus.Prevotella7.id.11182	IVW	11	−0.2097	0.1067	0.0494	0.7183	0.81	(0.66-1.00)	0.4895
	genus.RuminococcaceaeUCG002.id.11360	IVW	22	0.2988	0.1448	0.0391	0.7183	1.35	(1.02-1.79)	0.6077
	genus.Ruminiclostridium9.id.11357	IVW	8	−0.7144	0.2681	0.0077	0.6069	0.49	(0.29-0.83)	0.8219
	genus.Romboutsia.id.11347	IVW	13	−0.4376	0.1855	0.0183	0.6069	0.65	(0.45-0.93)	0.0794
	genus.Desulfovibrio.id.3173	IVW	10	−0.3700	0.1772	0.0369	0.7183	0.70	(0.49-0.98)	0.4168
	genus.Gordonibacter.id.821	IVW	12	−0.2404	0.1036	0.0204	0.6069	0.79	(0.64-0.96)	0.0728
	family.Clostridiaceae1.id.1869	IVW	10	−0.5168	0.2090	0.0134	0.4284	0.60	(0.4-0.9)	0.6167
	class.Mollicutes.id.3920	IVW	12	−0.4304	0.1737	0.0132	0.2114	0.65	(0.46-0.91)	0.4464
	phylum.Tenericutes.id.3919	IVW	12	−0.4304	0.1737	0.0132	0.0594	0.65	(0.46-0.91)	0.4464
	phylum.Actinobacteria.id.400	IVW	15	−0.4920	0.1917	0.0103	0.0594	0.61	(0.42-0.89)	0.0596
Cirrhosis	genus.Parasutterella.id.2892	IVW	15	−0.3465	0.1218	0.0045	0.4947	0.71	(0.56-0.9)	0.9751
	genus.Adlercreutzia.id.812	IVW	8	0.3774	0.1646	0.0219	0.4947	1.46	(1.06-2.01)	0.7214
	genus.Prevotella7.id.11182	IVW	11	−0.1625	0.0790	0.0397	0.6075	0.85	(0.73-0.99)	0.7510
	genus.Terrisporobacter.id.11348	IVW	5	−0.3841	0.1477	0.0093	0.4947	0.68	(0.51-0.91)	0.1616
	genus.Parabacteroides.id.954	IVW	6	−0.7549	0.3185	0.0178	0.4947	0.47	(0.25-0.88)	0.9196
	genus.Anaerofilum.id.2053	IVW	11	0.2387	0.1064	0.0249	0.4947	1.27	(1.03-1.56)	0.0649
	genus.Alistipes.id.968	IVW	14	0.3619	0.1605	0.0242	0.4947	1.44	(1.05-1.97)	0.1608
	family.Desulfovibrionaceae.id.3169	IVW	10	−0.3573	0.1459	0.0144	0.2957	0.70	(0.53-0.93)	0.7166
	family.Alcaligenaceae.id.2875	IVW	12	0.4701	0.1996	0.0185	0.2957	1.60	(1.08-2.37)	0.6856
	order.Desulfovibrionales.id.3156	IVW	12	−0.3319	0.1377	0.0160	0.3190	0.72	(0.55-0.94)	0.7958
	class.Deltaproteobacteria.id.3087	IVW	13	−0.3250	0.1400	0.0202	0.3236	0.72	(0.55-0.95)	0.7001
Hepatic failure, not elsewhere classified	genus.Prevotella9.id.11183	IVW	15	−0.5163	0.2386	0.0305	0.7414	0.60	(0.37-0.95)	0.1043
	genus.Enterorhabdus.id.820	IVW	6	−0.8923	0.3607	0.0134	0.7414	0.41	(0.2-0.83)	0.6494
	genus.Barnesiella.id.944	IVW	13	0.7116	0.3302	0.0311	0.7414	2.04	(1.07-3.89)	0.0873
	genus.Eubacteriumfissicatenagroup.id.14373	IVW	9	0.5782	0.2175	0.0078	0.7414	1.78	(1.16-2.73)	0.3804
	genus.Odoribacter.id.952	IVW	7	0.9581	0.4420	0.0302	0.7414	2.61	(1.1-6.2)	0.9552
	order.Selenomonadales.id.2165	IVW	12	−0.9091	0.3806	0.0169	0.3381	0.40	(0.19-0.85)	0.2283
	class.Negativicutes.id.2164	IVW	12	−0.9091	0.3806	0.0169	0.2705	0.40	(0.19-0.85)	0.2283
Benign neoplasm: Liver	genus.Prevotella9.id.11183	IVW	15	−0.9484	0.3350	0.0046	0.2763	0.39	(0.2-0.75)	0.1843
	genus.Enterorhabdus.id.820	IVW	6	−1.3545	0.5049	0.0073	0.2898	0.26	(0.1-0.69)	0.8032
	genus.Ruminococcustorquesgroup.id.14377	IVW	9	−1.4947	0.6529	0.0221	0.6565	0.22	(0.06-0.81)	0.5913
	genus.Ruminococcusgnavusgroup.id.14376	IVW	12	0.9907	0.3205	0.0020	0.2370	2.69	(1.44-5.05)	0.3915
	family.Alcaligenaceae.id.2875	IVW	11	−1.3394	0.5601	0.0168	0.5369	0.26	(0.09-0.79)	0.2252
	order.Burkholderiales.id.2874	IVW	10	−1.3120	0.5562	0.0183	0.3665	0.27	(0.09-0.8)	0.2892
	phylum.Actinobacteria.id.400	IVW	15	−0.9967	0.4682	0.0332	0.2682	0.37	(0.15-0.92)	0.8969
Malignant neoplasm of liver, primary	genus.Subdoligranulum.id.2070	IVW	11	−1.5685	0.7310	0.0319	0.6617	0.21	(0.05-0.87)	0.2270
	genus.Catenibacterium.id.2153	IVW	5	−1.2793	0.4822	0.0080	0.4766	0.28	(0.11-0.72)	0.2049
	genus.Paraprevotella.id.962	IVW	13	−0.8992	0.4226	0.0334	0.6617	0.41	(0.18-0.93)	0.8384
	genus.Eubacteriumnodatumgroup.id.11297	IVW	11	−0.7681	0.3300	0.0199	0.6617	0.46	(0.24-0.89)	0.1670
	genus.Howardella.id.2000	IVW	10	0.9906	0.3736	0.0080	0.4766	2.69	(1.29-5.6)	0.3932
	genus.LachnospiraceaeUCG004.id.11324	IVW	12	1.2911	0.6504	0.0471	0.7511	3.64	(1.02-13.01)	0.7299
	genus.Veillonella.id.2198	IVW	7	−1.4996	0.7001	0.0322	0.6617	0.22	(0.06-0.88)	0.1876
	family.ClostridialesvadinBB60group.id.11286	IVW	15	1.4126	0.4893	0.0039	0.1244	4.11	(1.57-10.71)	0.5377
	family.Oxalobacteraceae.id.2966	IVW	14	−0.8266	0.3585	0.0211	0.3382	0.44	(0.22-0.88)	0.3853

#### 3.1.1 Alcoholic liver disease

**FIGURE 3:**
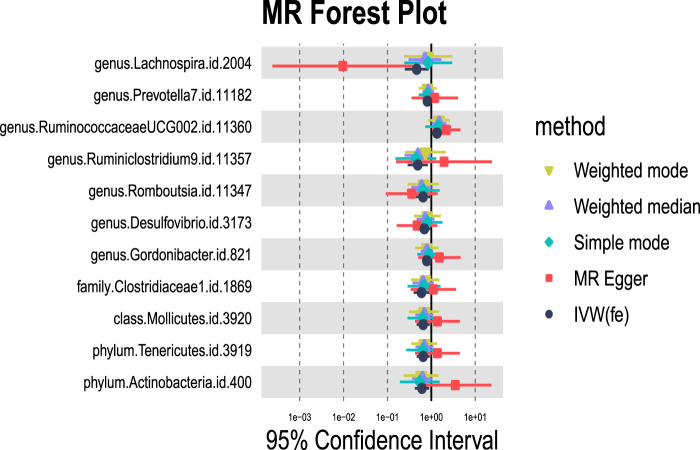
MR forest plot of gut microbiota significantly associated with alcoholic liver disease. The *x*-axis represents the 95% confidence interval for each genus, while the *y*-axis lists the specific genera. Various MR methods are represented by different colored lines and symbols: yellow for Weighted Mode, purple for Weighted Median, green for Simple Mode, red for MR Egger, and black for IVW.

**FIGURE 4:**
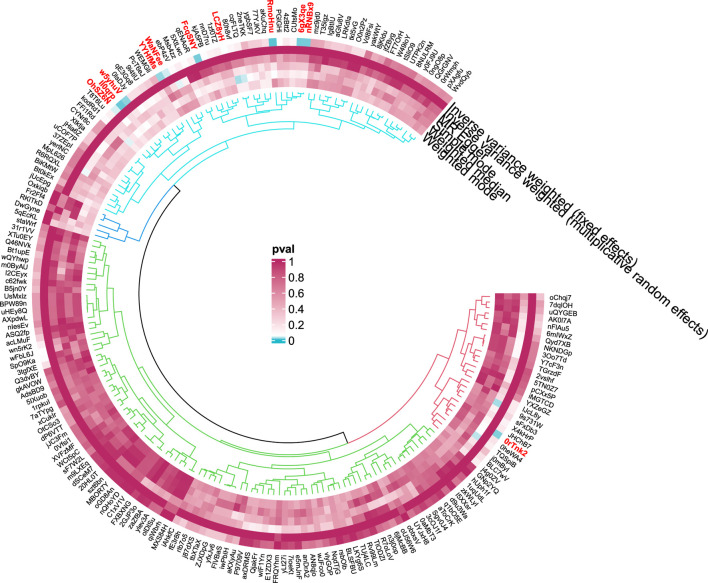
Circular diagram of gut microbiota in alcoholic liver disease. In the circular plot, the colors represent different levels of significance. Red indicates lower p-values (higher significance), suggesting a potential causal relationship between these microbes and the disease, Blue represents lower significance.

We identified 11 potential causal relationships between GM and ALD (Figures 3, 4). However, these associations did not remain significant after adjusting for BH correction. Specifically, ten types of GM were found to be associated with a reduced risk of ALD, including Lachnospira (OR = 0.46, 95% CI = 0.25-0.87, *P*_IVW_ = 0.0160,*P*_adj_ = 0.6069), Prevotella 7(OR = 0.81, 95% CI = 0.66-1.00, *P*_IVW_ = 0.0494,*P*_adj_ = 0.7183), Ruminiclostridium 9(OR = 0.49, 95% CI = 0.29-0.83, *P*_IVW_ = 0.0077,*P*_adj_ = 0.6069), Romboutsia (OR = 0.65, 95% CI = 0.45-0.93, *P*_IVW_ = 0.0183,*P*_adj_ = 0.6069), Desulfovibrio (OR = 0.70, 95% CI = 0.49-0.98, *P*_IVW_ = 0.0369,*P*_adj_ = 0.7183), Gordonibacter (OR = 0.79, 95% CI = 0.64-0.96, *P*_IVW_ = 0.0204,*P*_adj_ = 0.6069), Clostridiaceae 1(OR = 0.60, 95% CI = 0.4-0.9, *P*_IVW_ = 0.0134,*P*_adj_ = 0.4284), Mollicutes (OR = 0.65, 95% CI = 0.46-0.91, *P*_IVW_ = 0.0132,*P*_adj_ = 0.2114), Tenericutes (OR = 0.65, 95% CI = 0.46-0.91, *P*_IVW_ = 0.0132,*P*_adj_ = 0.0594), Actinobacteria (OR = 0.61, 95% CI = 0.42-0.89, *P*_IVW_ = 0.0103,*P*_adj_ = 0.0594). Conversely, an increased risk of ALD was observed with Ruminococcaceae UCG002(OR = 1.35, 95% CI = 1.02-1.79, *P*_IVW_ = 0.0391,*P*_adj_ = 0.7183).

#### 3.1.2 Cirrhosis

**FIGURE 5:**
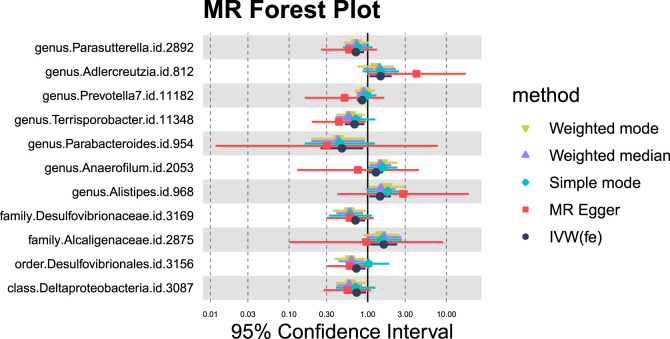
MR forest plot of gut microbiota significantly associated with cirrhosis.

**FIGURE 6:**
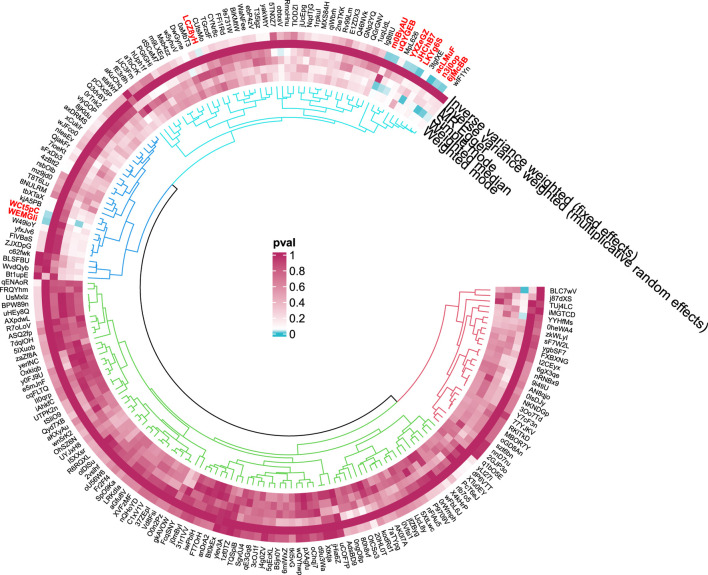
Circular diagram of gut microbiota in cirrhosis.

In the case of cirrhosis, 10 potential causal relationships were observed (Figures 5, 6). Although these did not remain significant after adjusting for BH correction, six GM taxa showed a nominal association with reduced cirrhosis risk, including Parasutterella (OR = 0.71,95% CI = 0.56-0.9, *P*_IVW_ = 0.0045,*P*_adj_ = 0.4947), Prevotella 7(OR = 0.85,95% CI = 0.73-0.99, *P*_IVW_ = 0.0397,*P*_adj_ = 0.6075), Terrisporobacter (OR = 0.68,95% CI = 0.51-0.91, *P*_IVW_ = 0.0093,*P*_adj_ = 0.4947), Parabacteroides (OR = 0.47,95% CI = 0.25-0.88, *P*_IVW_ = 0.0178,*P*_adj_ = 0.4947), Desulfovibrionaceae (OR = 0.70,95% CI = 0.53-0.93, *P*_IVW_ = 0.0144,*P*_adj_ = 0.2957), Desulfovibrionales (OR = 0.72,95% CI = 0.55-0.94, *P*_IVW_ = 0.0160,*P*_adj_ = 0.3190), and Deltaproteobacteria (OR = 0.72,95% CI = 0.55-0.95, *P*_IVW_ = 0.0202,*P*_adj_ = 0.3236). On the other hand, four taxa, including Adlercreutzia (OR = 1.46,95% CI = 1.06-2.01, *P*_IVW_ = 0.0219,*P*_adj_ = 0.4947), Anaerofilum (OR = 1.27,95% CI = 1.03-1.56, *P*_IVW_ = 0.0249,*P*_adj_ = 0.4947), Alistipes (OR = 1.44,95% CI = 1.05-1.97, *P*_IVW_ = 0.0242,*P*_adj_ = 0.4947), and Alcaligenaceae (OR = 1.60,95% CI = 1.08-2.37, *P*_IVW_ = 0.0185,*P*_adj_ = 0.2957) were nominally linked to an increased risk of cirrhosis.

#### 3.1.3 Hepatic failure

**FIGURE 7:**
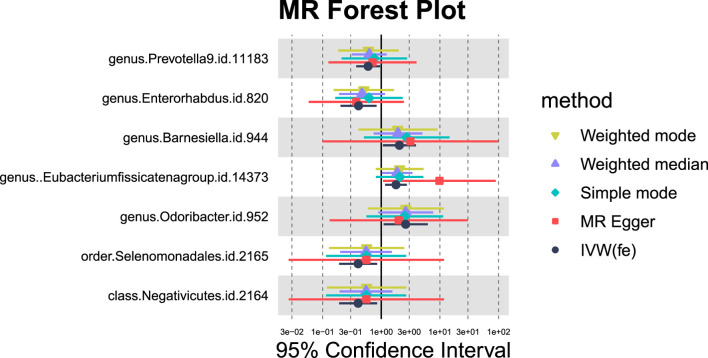
MR forest plot of gut microbiota significantly associated with hepatic failure, not elsewhere classified.

**FIGURE 8:**
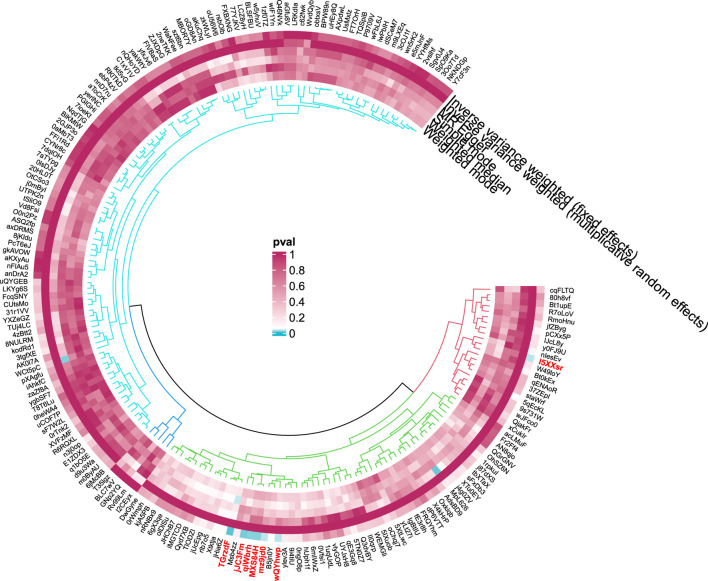
Circular diagram of gut microbiota in hepatic failure, not elsewhere classified.

For hepatic failure, 7 GM taxa showed potential causal relationships (Figures 7, 8). However, none reached significance after adjusting for BH correction. Four taxa, including Prevotella 9 (OR = 0.60,95% CI = 0.37-0.95, *P*_IVW_ = 0.0305, *P*_adj_ = 0.7414), Enterorhabdus (OR = 0.41,95% CI = 0.2-0.83, *P*_IVW_ = 0.0134, *P*_adj_ = 0.7414), Selenomonadales (OR = 0.40,95% CI = 0.19-0.85,*P*_IVW_ = 0.0169, *P*_adj_ = 0.3381), and Negativicutes (OR = 0.40,95% CI = 0.19-0.85, *P*_IVW_ = 0.0169, *P*_adj_ = 0.2705). Conversely, three types of GM were nominally associated with an increased risk of hepatic failure, including Barnesiella (OR = 2.04,95% CI = 1.07-3.89, *P*_IVW_ = 0.0311, *P*_adj_ = 0.7414), Eubacterium fissicatenagroup (OR = 1.78,95% CI = 1.16-2.73, *P*_IVW_ = 0.0078, *P*_adj_ = 0.7414) and Odoribacter (OR = 2.61,95% CI = 1.1-6.2, *P*_IVW_ = 0.0302, *P*_adj_ = 0.7414).

#### 3.1.4 Liver benign neoplasm

**FIGURE 9:**
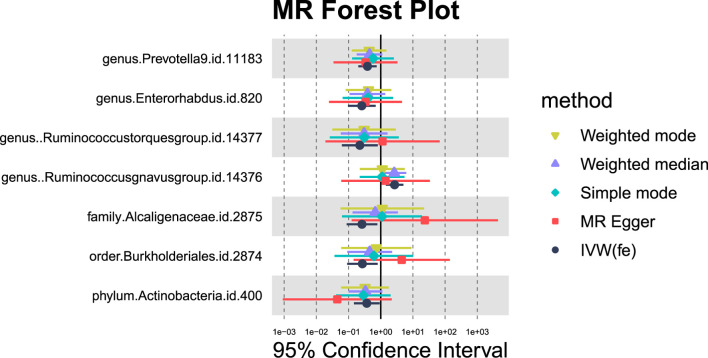
MR Forest plot of gut microbiota significantly associated with “Benign neoplasm: Liver”.

**FIGURE 10:**
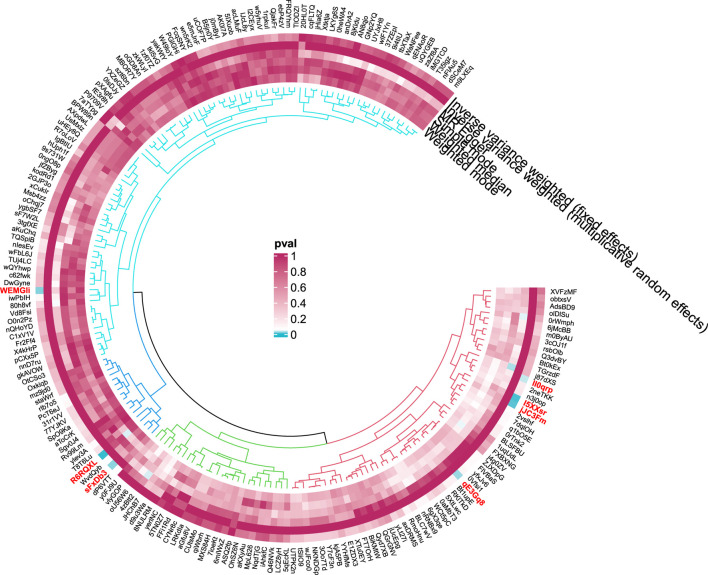
Circular diagram of gut microbiota in “Benign neoplasm: Liver”.

Seven GM species exhibit potential causal relationships with hepatic failure, not elsewhere classified (Figures 9, 10). However, these associations did not reach significance levels after BH correction. Specifically, six types of GM are nominally associated with a reduced risk of benign neoplasm in the liver, including Prevotella9 (OR = 0.39, 95%CI = 0.2-0.75, *P*_IVW_ = 0.0046,*P*_adj_ = 0.2763), Enterorhabdus (OR = 0.26, 95%CI = 0.1-0.69,*P*_IVW_ = 0.0073,*P*_adj_ = 0.2898), Ruminococcustorquesgroup (OR = 0.22, 95%CI = 0.06-0.81, *P*_IVW_ = 0.0221,*P*_adj_ = 0.6565), Alcaligenaceae (OR = 0.26,95%CI = 0.09-0.79, *P*_IVW_ = 0.0168,*P*_adj_ = 0.5369), Burkholderiales (OR = 0.27,95%CI = 0.09-0.8,*P*_IVW_ = 0.0183,*P*_adj_ = 0.3665), and Actinobacteria (OR = 0.37,95%CI = 0.15-0.92,*P*_IVW_ = 0.0332,*P*_adj_ = 0.2682). Conversely, Ruminococcusgnavusgroup is nominally associated with an increased risk of benign neoplasm in the liver (OR = 2.69, 95% CI = 1.44-5.05, *P*_IVW_ = 0.0020,*P*_adj_ = 0.2370).

#### 3.1.5 Malignant neoplasm of liver, primary

**FIGURE 11:**
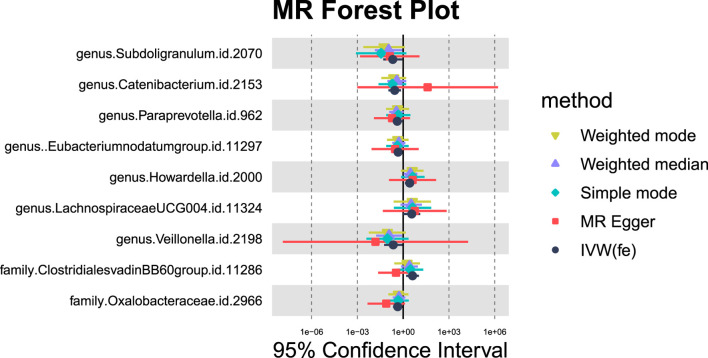
MR forest plot of gut microbiota significantly associated with “Malignant neoplasm of liver, primary”.

**FIGURE 12:**
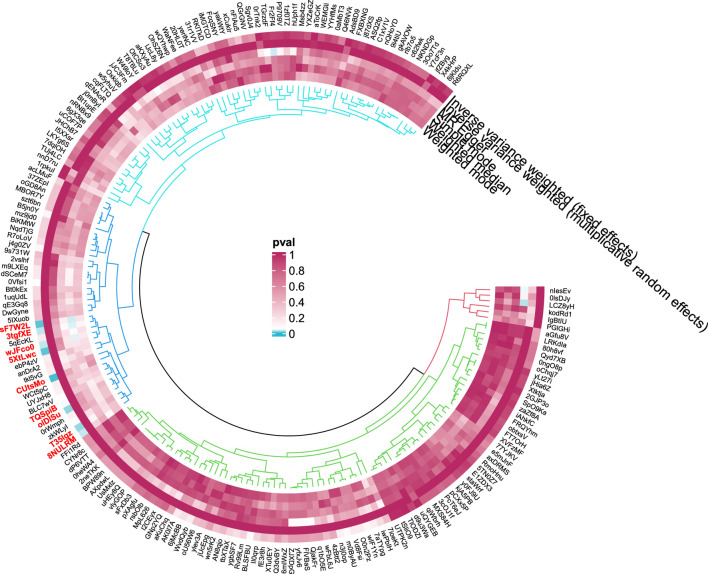
Circular diagram of gut microbiota in “Malignant neoplasm of liver, primary”.

Nine GM taxa were found to be nominally associated with primary malignant liver neoplasms (Figures 11, 12). After BH correction, none reached statistical significance. Specifically, six types of GM are nominally associated with a reduced risk of primary malignant neoplasm of the liver, including Subdoligranulum (OR = 0.21, 95% CI = 0.05-0.87, *P*_IVW_ = 0.0319, *P*_adj_ = 0.6617), Catenibacterium (OR = 0.28, 95% CI = 0.11-0.72, *P*_IVW_ = 0.0080, *P*_adj_ = 0.4766), Paraprevotella (OR = 0.41, 95% CI = 0.18-0.93, *P*_IVW_ = 0.0334, *P*_adj_ = 0.6617), Eubacterium nodatum group (OR = 0.46, 95% CI = 0.24-0.89, *P*_IVW_ = 0.0199, *P*_adj_ = 0.6617), Veillonella (OR = 0.22, 95% CI = 0.06-0.88, *P*_IVW_ = 0.0322, *P*_adj_ = 0.6617), and Oxalobacteraceae (OR = 0.44, 95% CI = 0.22-0.88, *P*_IVW_ = 0.0211, *P*_adj_ = 0.3382). Conversely, Howardella (OR = 2.69, 95% CI = 1.29-5.60, *P*_IVW_ = 0.0080, *P*_adj_ = 0.4766), Lachnospiraceae UCG004 (OR = 3.64, 95% CI = 1.02-13.01, *P*_IVW_ = 0.0471, *P*_adj_ = 0.7511), and Clostridiales vadinBB60 group (OR = 4.11, 95% CI = 1.57-10.71, *P*_IVW_ = 0.0039, *P*_adj_ = 0.1244) are nominally associated with an increased risk of primary malignant neoplasm of the liver.

#### 3.1.6 Sensitivity analysis

In the sensitivity analysis of the aforementioned five liver diseases-associated bacterial taxa, the p-values from both MR-Egger and MR-PRESSO tests were less than 0.05, indicating the absence of horizontal pleiotropy and outliers. All p-values in the Cochrane’s Q test were greater than 0.05, indicating no significant heterogeneity. All the results are elaborated in the annex 1.

**FIGURE 13:**
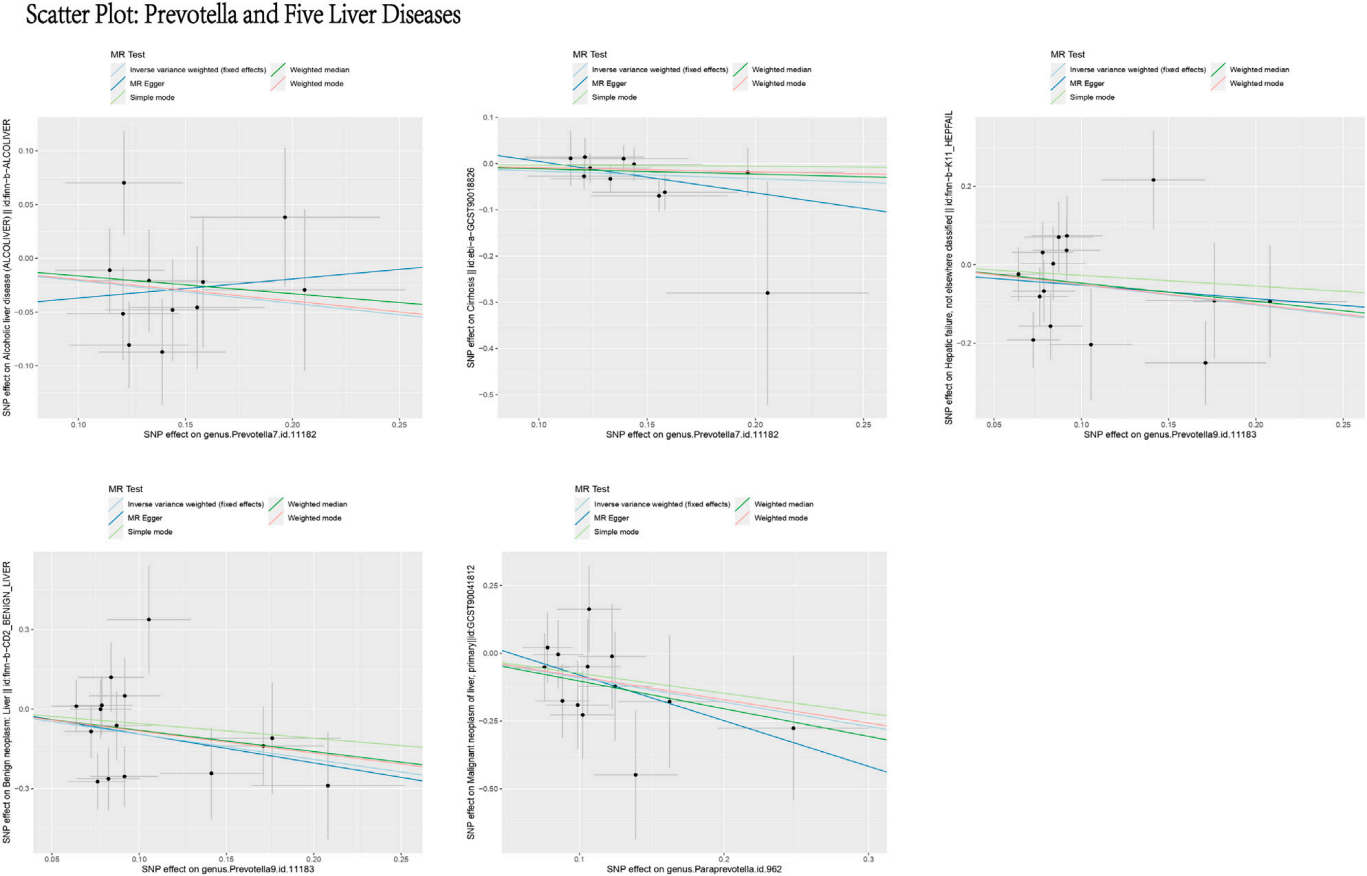
Scatter plot: prevotella and five liver diseases. This figure illustrates the causal relationship between Prevotella and five liver diseases using different MR methods. The *X*-axis represents the SNP effects on Prevotella (exposure), and the *Y*-axis represents the SNP effects on liver diseases (outcome). The different line colors represent various MR methods, including light blue (IVW, fixed effects), dark blue (MR Egger), light green (Simple mode), dark green (Weighted median), and red (Weighted mode). Overall, the causal effect of Prevotella on these five liver diseases appears to be protective.

**FIGURE 14:**
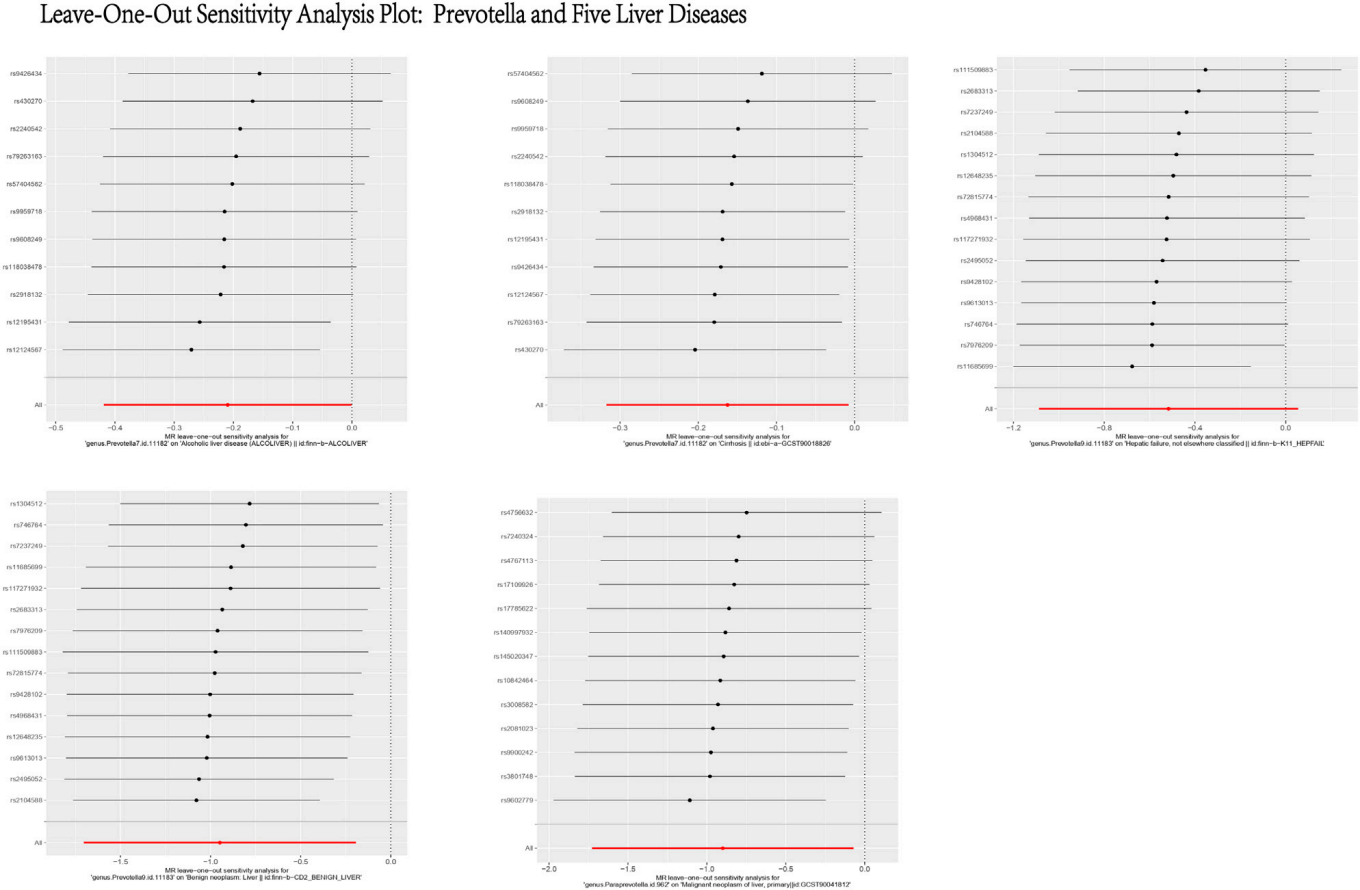
Leave-One-Out Sensitivity Analysis Plot: Prevotella and Five Liver Diseases. This figure presents the results of a Leave-One-Out Sensitivity Analysis, which evaluates the robustness of the causal relationship between Prevotella and five liver diseases by removing individual SNPs. The *X*-axis represents the effect of each SNP on the association between Prevotella and the respective liver disease, while the *Y*-axis lists the SNP IDs. Each black dot indicates the effect estimate after excluding a specific SNP, with the horizontal lines representing the 95% confidence intervals. The analysis demonstrates that the causal relationship between Prevotella and the five liver diseases remains generally robust, as the removal of most SNPs does not significantly alter the results.

Based on the MR analysis of GM and various liver diseases in the above table, We were surprised to find that Prevotella, a genus of gut bacteria, consistently appeared as a protective factor in all five liver diseases (Figures 13, 14). Liu (Liu et al., 2021) et al. confirmed that afterFMT treatment, beneficial bacterial genera like Prevotella increased, leading to a reduction in liver damage induced by D-GALN in mice. Additionally, probiotics shifted the GM towards beneficial bacteria, including Prevotella, thereby suppressing the growth of liver cell carcinoma in mice (Li et al., 2016), These scholars’ observations align with the findings of our study.

### 3.2 Single-cell analysis results

**FIGURE 15:**
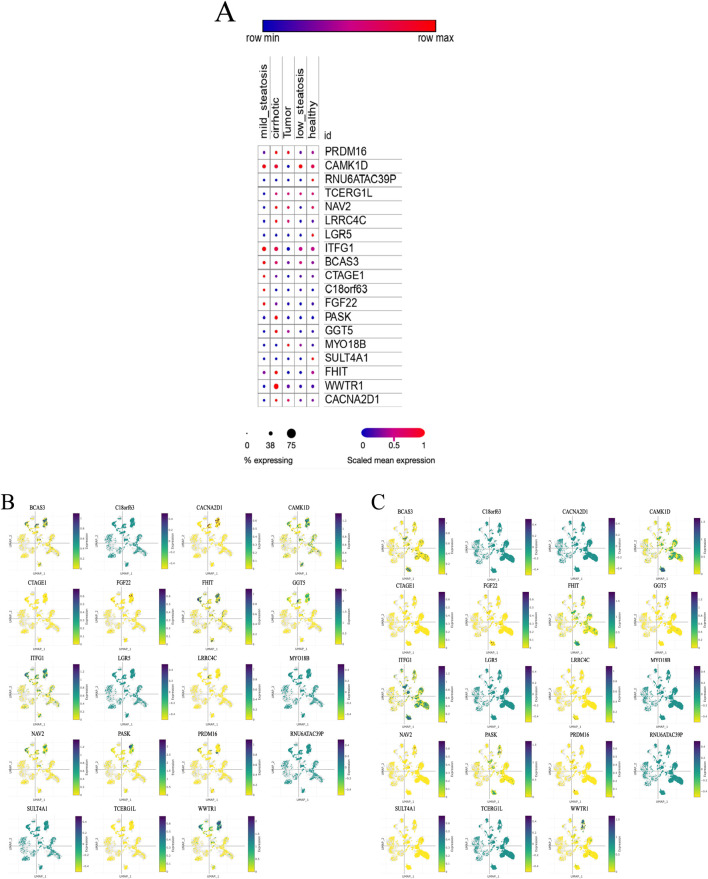
Single-cell analysis results. **(A)** Expression levels of Prevotella-related genes across different liver health or disease states. **(B)** UMAP expression distribution of Prevotella-related genes in alcoholic cirrhosis patients. **(C)** UMAP expression distribution of Prevotella-related genes in healthy liver samples. **(A)** illustrates five conditions: mild steatosis, tumor, low steatosis, cirrhosis, and healthy. The expression level of each gene under these conditions is represented by dots. The color and size of the dots reflect expression levels and the percentage of samples expressing the gene. Dot color ranges from blue to red, indicating low to high gene expression, while the size of the dots corresponds to the percentage of samples in which the gene is expressed, with smaller dots representing low expression and larger dots indicating a higher percentage of expression. **(B, C)** present a two-dimensional visualization of the expression distribution of Prevotella-related genes using UMAP. Each plot represents a specific gene, showing its expression pattern across different cells or samples. The UMAP axes (*X*-axis labeled “UMAP-1” and *Y*-axis labeled “UMAP-2”) represent the reduced dimensional coordinates, revealing the underlying structure within the cell samples. The color scale to the right of the plots ranges from yellow (low expression) to purple (high expression). The dots in the plots represent individual cell samples, with darker purple indicating higher gene expression in those cells, while yellow dots indicate lower expression.

We analyzed scRNA-seq data from 64 samples (except unknown), including 38 healthy controls and 26 liver disease patients. Using genetic variants linked to Prevotella as instrumental variables, the relationship between these IVs and their associated genes is outlined in detail in Annex 1. We examined the expression of these genes under different liver conditions. The results revealed that 18 genes related to Prevotella showed differential expression between healthy individuals and various liver disease states, which is visually represented in the bubble plot (Figure 15A). Further UMAP analysis indicated that the expression levels of genes such as PRDM16 and WWTR1 were elevated in alcoholic cirrhosis patients, while other genes like C18orf63 and RNU6ATAC39P showed decreased expression (Figures 15B, C). These findings indicate that Prevotella-related genes may be critical in the progression of liver diseases.

## 4 Summary and discussions

**FIGURE 16:**
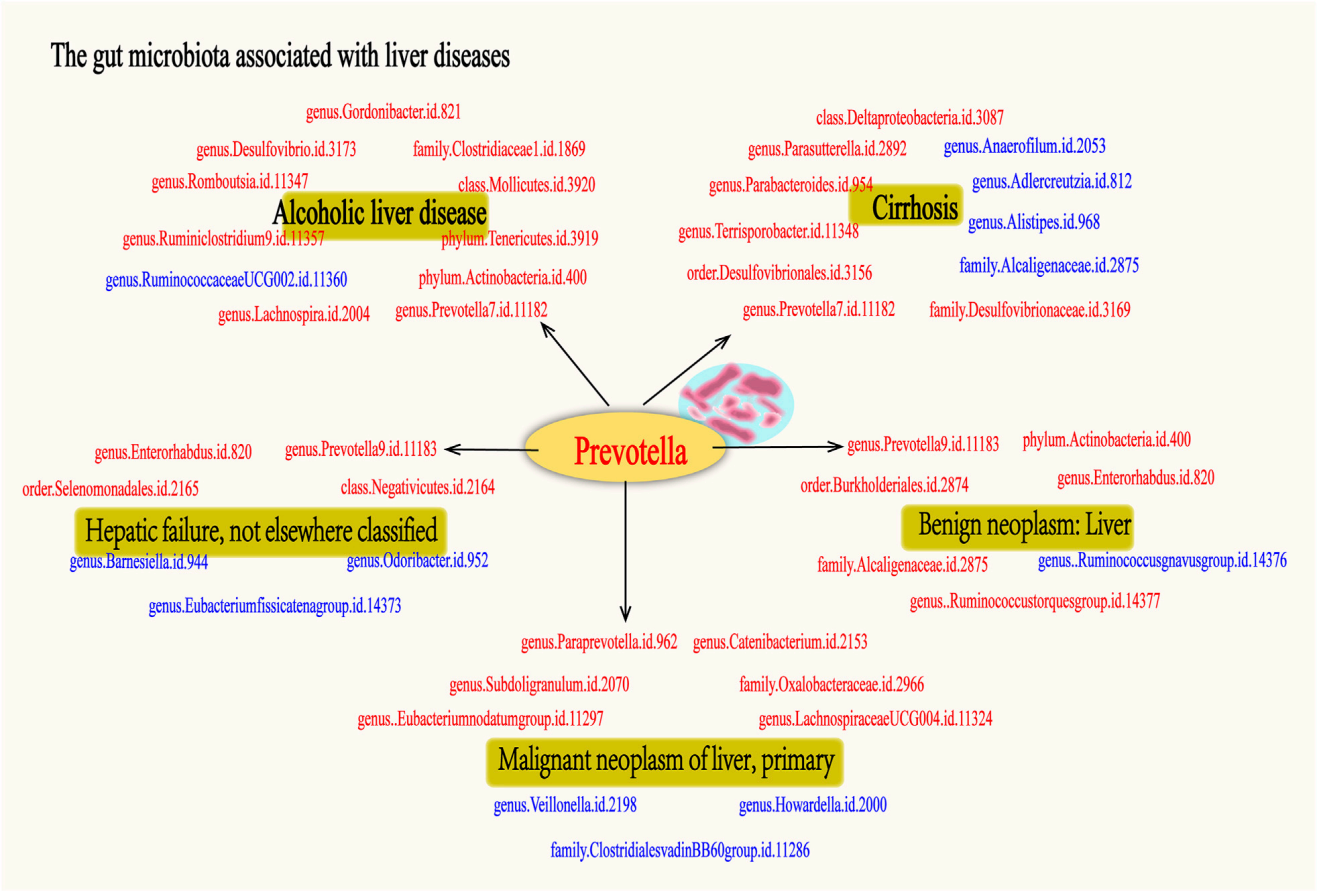
The gut microbiota associated with fiver live diseases. The figure illustrates the associated with five liver diseases: alcoholic liver disease, cirrhosis, hepatic failure, benign liver tumors, and primary malignant liver tumors. Microbiota shown in red indicates a potential protective effect on the liver, suggesting their beneficial role in mitigating disease progression. In contrast, microbiota depicted in blue represent risk factors, which may contribute to the development or worsening of these liver diseases. Each group of gut microbiota is associated with specific liver conditions, highlighting the complex interplay between gut microbiota and liver pathology. Notably, the Prevotella genus is consistently identified as a protective factor across all five liver diseases, underscoring its potential in liver disease treatment.

As far as we are aware, this study represents the first MR investigation of the causal relationships between GM and various liver diseases. In order to explore the regularities between these factors, we conducted a comprehensive analysis of the GM associated with the nominally significant taxa in “ALD”, “Cirrhosis”, “Hepatic failure, not elsewhere classified”, “Benign neoplasm: Liver”, and “Malignant neoplasm of liver, primary”. The results indicate that ALD has 10 positive causal directions and 1 negative causal direction; Cirrhosis has 7 positive causal directions and 4 negative causal directions; Hepatic failure, not elsewhere classified has 4 positive causal directions and 3 negative causal directions; Benign neoplasm: Liver has 6 positive causal directions and 1 negative causal direction; Malignant neoplasm of liver, primary has 6 positive causal directions and 3 negative causal directions (Figure 16). Notably, as shown in Figure 16, Prevotella is considered a protective factor in the five liver diseases mentioned. Additionally, our single-cell analysis results indicate that genes related to Prevotella exhibit significant expression differences across different liver conditions. Through further literature research, we found that Prevotella bacteria have been recognized as beneficial microbiota involved in the regulation of various diseases.

Prevotella belongs to the phylum Bacteroidetes, class Bacteroidia, and genus Prevotella. It is a group of Gram-negative bacteria and constitutes a fundamental component of the human GM. It is widely present in human mucosal, respiratory, and intestinal ecosystems. It plays a key role in regulating the host’s metabolic health and maintaining immune balance and is considered a critical participant in the balance between health and disease (Tett et al., 2021). Chang et al. (2019) have already regarded it as a promising candidate for the next-generation probiotic. Prevotella is generally recognized as a bacterium associated with plant-based diets (De Filippis et al., 2016; Ruengsomwong et al., 2016). The intake of dietary fibers facilitates the colonization of Prevotella in the intestines, which not only helps maintain the balance of the GM but also enhances the host’s metabolic functions through various pathways (Shen et al., 2017). Firstly, Prevotella participates in the fermentation of polysaccharides (Fehlner-Peach et al., 2019), effectively breaking down dietary fibers into short-chain fatty acids (SCFAs) such as acetate and butyrate (Kumari et al., 2022) These SCFAs play a crucial role in regulating intestinal homeostasis, adipose tissue, and liver metabolism (Canfora et al., 2015). Acetate, one of the most common SCFAs, enters the circulatory system to influence whole-body energy metabolism and serves as a precursor for lipid synthesis, aiding in fat storage and liver fat metabolism regulation (Frost et al., 2014). Butyrate, as a primary energy source for intestinal epithelial cells, helps maintain the integrity of the gut barrier and reduces the translocation of harmful substances into the bloodstream (Morrison and Preston, 2016). Furthermore, SCFAs inhibit inflammation through multiple mechanisms, thereby reducing liver damage. For instance, they elevate anti-inflammatory Treg cell levels, lower metabolic endotoxemia, and decrease the expression of pro-inflammatory adipocytokines and chemokines (Al-Lahham et al., 2012). Research also indicates that SCFAs regulate gut hormones such as glucagon-like peptide-1, leptin, and peptide YY, which help maintain energy balance and prevent metabolic diseases such as obesity, dysregulated glucose and lipid metabolism, and NAFLD (De Vadder et al., 2014). Secondly, the succinates produced by Prevotella (Kovatcheva-Datchary et al., 2015), as intermediates in the tricarboxylic acid cycle, not only regulate intestinal gluconeogenesis to maintain host glucose homeostasis but also influence the host’s energy metabolism, aiding in the prevention of insulin resistance and associated metabolic diseases (De Vadder et al., 2016). Concurrently, Prevotella coexists with other bacterial phyla such as Actinobacteria, Firmicutes, Proteobacteria, and Archaea, forming a complex gut ecosystem that enhances carbohydrate fermentation (Kovatcheva-Datchary et al., 2015).

However, under certain pathological conditions, some Prevotella species may exhibit pathogenic characteristics. For example, Prevotella can activate Toll-like receptor 2, driving systemic Th-cell-mediated immune responses that may exacerbate inflammation (Larsen, 2017). Research shows that an overgrowth of Prevotella is associated with the progression of liver inflammation and fatty liver disease (Schnabl and Brenner, 2014). Furthermore, specific metabolic products of Prevotella, such as succinate, may exacerbate inflammation under certain pathological conditions and are associated with insulin resistance and hepatic fat deposition (Mills et al., 2016). This dual nature suggests that we must carefully assess GM before clinical application.

Our results suggest that Prevotella may serve as a potential therapeutic target in the liver, which aligns with previous research. For example, Boursier (Boursier et al., 2016) et al. found that the abundance of Prevotella in non-alcoholic fatty liver disease (NAFLD) patients tends to decrease with the severity of liver damage. Shen (Shen et al., 2017) et al. further confirmed that a reduction in Prevotella levels may exacerbate NAFLD. Zhang (Zhang et al., 2023) et al. conducted an MR study that confirmed Prevotella 7 as a protective factor for chronic hepatitis B (CHB), suggesting that Prevotella 7 may reduce the risk of CHB by modulating host inflammation and immune responses; Jiang (Jiang et al., 2022) et al., in a mouse model of primary sclerosing cholangitis (PSC) induced by 3,5-diethoxycarbonyl-1,4-dihydrocollidine (DDC), found that Copri enhances the FXR-related signaling pathway, leading to a significant improvement in bile stasis and liver fibrosis. It is evident that Prevotella may serve as a potential therapeutic strategy for various liver diseases. This protective mechanism may be attributed to Prevotella’s involvement in regulating intestinal immunity, enhancing the stability of the intestinal mucosal barrier, restricting the translocation of microbial metabolites, preventing the promotion of liver inflammation by metabolites, and participating in the regulation of inflammatory responses. Thereby exerting a protective effect and reducing the risk of liver diseases.

Although in this study we used MR as the research method to establish the causal relationship between GM and liver diseases, effectively eliminating the influence of confounding factors; The genetic variations of the GM were obtained from the largest GWAS meta-analysis, ensuring the strength of the instruments in the MR analysis; The utilization of MR-PRESSO and MR-Egger to eliminate horizontal pleiotropy ensured the authenticity and reliability of the study results. However, it is important to acknowledge certain limitations: 1、Although Mendelian Randomization is a powerful tool, it can only identify associations and cannot definitively determine causality. Therefore, single-cell analysis studies are necessary to further validate the causal relationship between gut microbiota and liver diseases. 2、Some Bacterial taxonomic groups were only analyzed at the order or family level, limiting further exploration. 3、The outcome data in the analysis were aggregated statistics without differentiation among different disease subtypes, precluding subgroup analysis. 4、Single-cell transcriptome sequencing and high-throughput RNA sequencing data come with certain technical limitations and interpretation challenges, such as managing data noise, identifying and annotating cell types, and data normalization. Further validation is crucial for accurately interpreting gene expression in single-cell analysis. In summary, our study results provide support for the potential causal influence of GM on liver diseases. We propose that Prevotella could be considered a liver-friendly microbial community, making treatments such as probiotics and FMT more targeted for liver disease management. While Prevotella is generally considered a beneficial bacterium, specific species and strains of Prevotella may also have varying effects on human health. Hence, there is a need for further research to explore the mechanisms by which specific species and strains of Prevotella impact liver diseases, and validation of these discoveries will rely on future RCTs.

$$f\left(x\right)=a_{0}+\sum_{n=1}^{\infty }\left(a_{n}\cos\frac{n\pi x}{L}+b_{n}\sin\frac{n\pi x}{L}\right)$$

## Data Availability

The datasets presented in this study can be found in online repositories. The names of the repository/repositories and accession number(s) can be found in the article/supplementary material.
