# Supplementary material for: Causal impact of gut microbiota on five liver diseases: insights from mendelian randomization and single-cell RNA sequencing
Source: Front Genet. 2024 Nov 11;15:1362139. doi: 10.3389/fgene.2024.1362139 (PMC11586359; doi:10.3389/fgene.2024.1362139)
Supplement: Supplementary file 1 [file DataSheet1.zip › Annex 1 _Data/MR results/Alcoholic liver disease/Alcoholic liver disease -figures/Alcoholic liver disease-Forest plot .pdf]

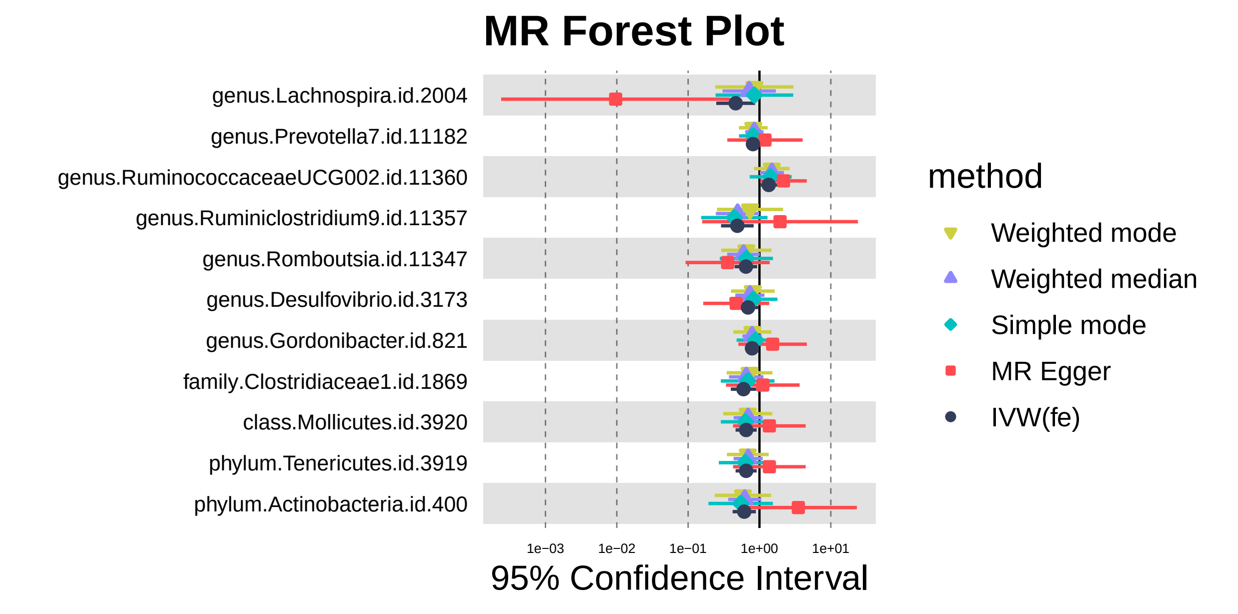
MR Forest Plot
genus.Lachnospira.id.2004
genus.Prevotella7.id.11182
genus.RuminococcaceaeUCG002.id.11360
genus.Ruminiclostridium9.id.11357
genus.Romboutsia.id.11347
genus.Desulfovibrio.id.3173
genus.Gordonibacter.id.821
family.Clostridiaceae1.id.1869
class.Mollicutes.id.3920
phylum.Tenericutes.id.3919
phylum.Actinobacteria.id.400
1e−03
1e−02
1e−01
1e+00
1e+01
95% Confidence Interval
method
Weighted mode
Weighted median
Simple mode
MR Egger
IVW(fe)
