# Supplementary material for: Causal impact of gut microbiota on five liver diseases: insights from mendelian randomization and single-cell RNA sequencing
Source: Front Genet. 2024 Nov 11;15:1362139. doi: 10.3389/fgene.2024.1362139 (PMC11586359; doi:10.3389/fgene.2024.1362139)
Supplement: Supplementary file 1 [file DataSheet1.zip › Annex 1 _Data/MR results/Alcoholic liver disease/Alcoholic liver disease -figures/LeaveOne_finn-b-ALCOLIVER_class.Alphaproteobacteria.id.2379.pdf]

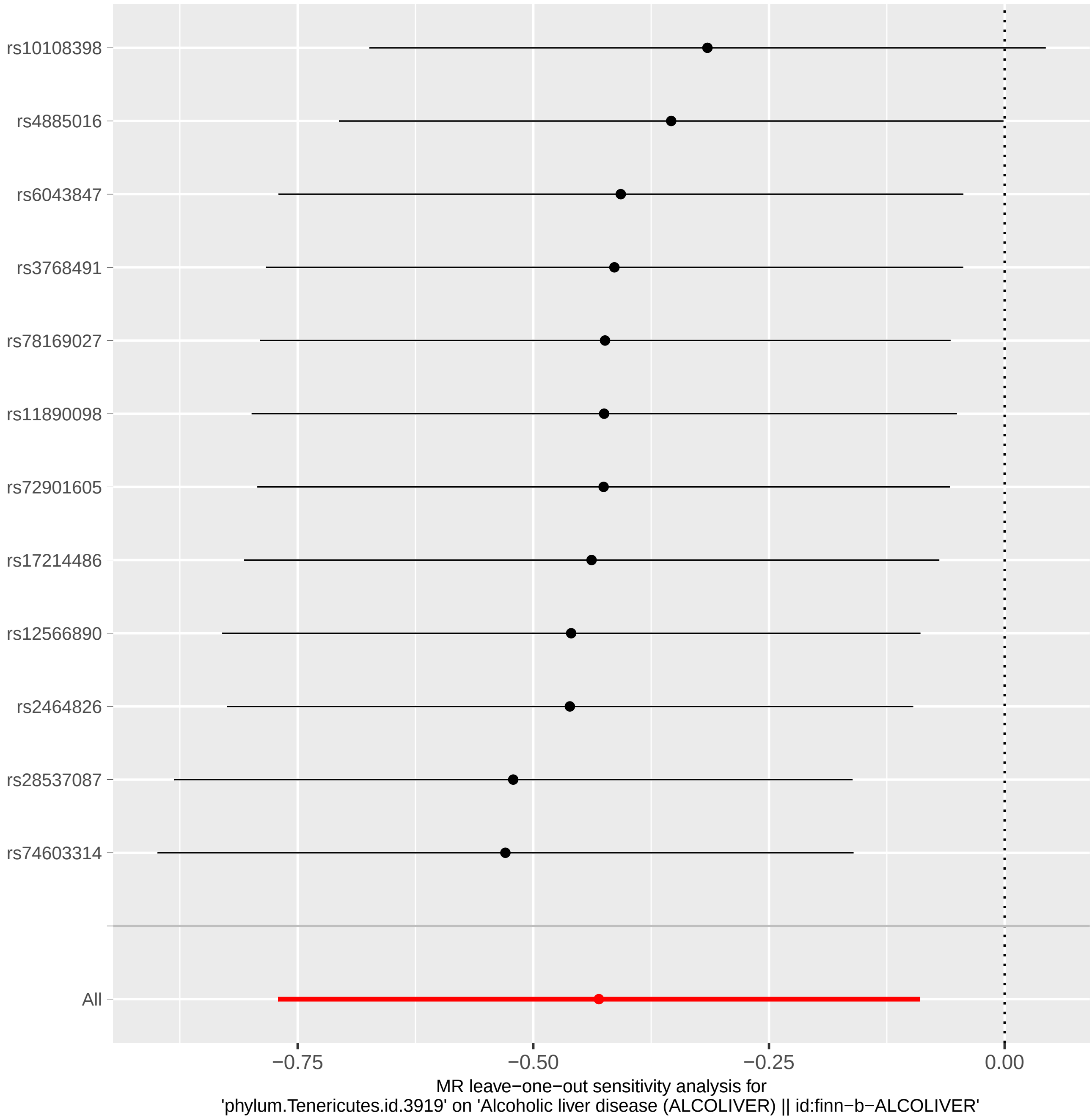
rs10108398
rs4885016
rs6043847
rs3768491
rs78169027
rs11890098
rs72901605
rs17214486
rs12566890
rs2464826
rs28537087
rs74603314
All
−0.75
−0.50
−0.25
0.00
MR leave−one−out sensitivity analysis for
'phylum.Tenericutes.id.3919' on 'Alcoholic liver disease (ALCOLIVER) || id:finn−b−ALCOLIVER'
