# Supplementary material for: Causal impact of gut microbiota on five liver diseases: insights from mendelian randomization and single-cell RNA sequencing
Source: Front Genet. 2024 Nov 11;15:1362139. doi: 10.3389/fgene.2024.1362139 (PMC11586359; doi:10.3389/fgene.2024.1362139)
Supplement: Supplementary file 1 [file DataSheet1.zip › Annex 1 _Data/MR results/Alcoholic liver disease/Alcoholic liver disease -figures/LeaveOne_finn-b-ALCOLIVER_class.Bacilli.id.1673.pdf]

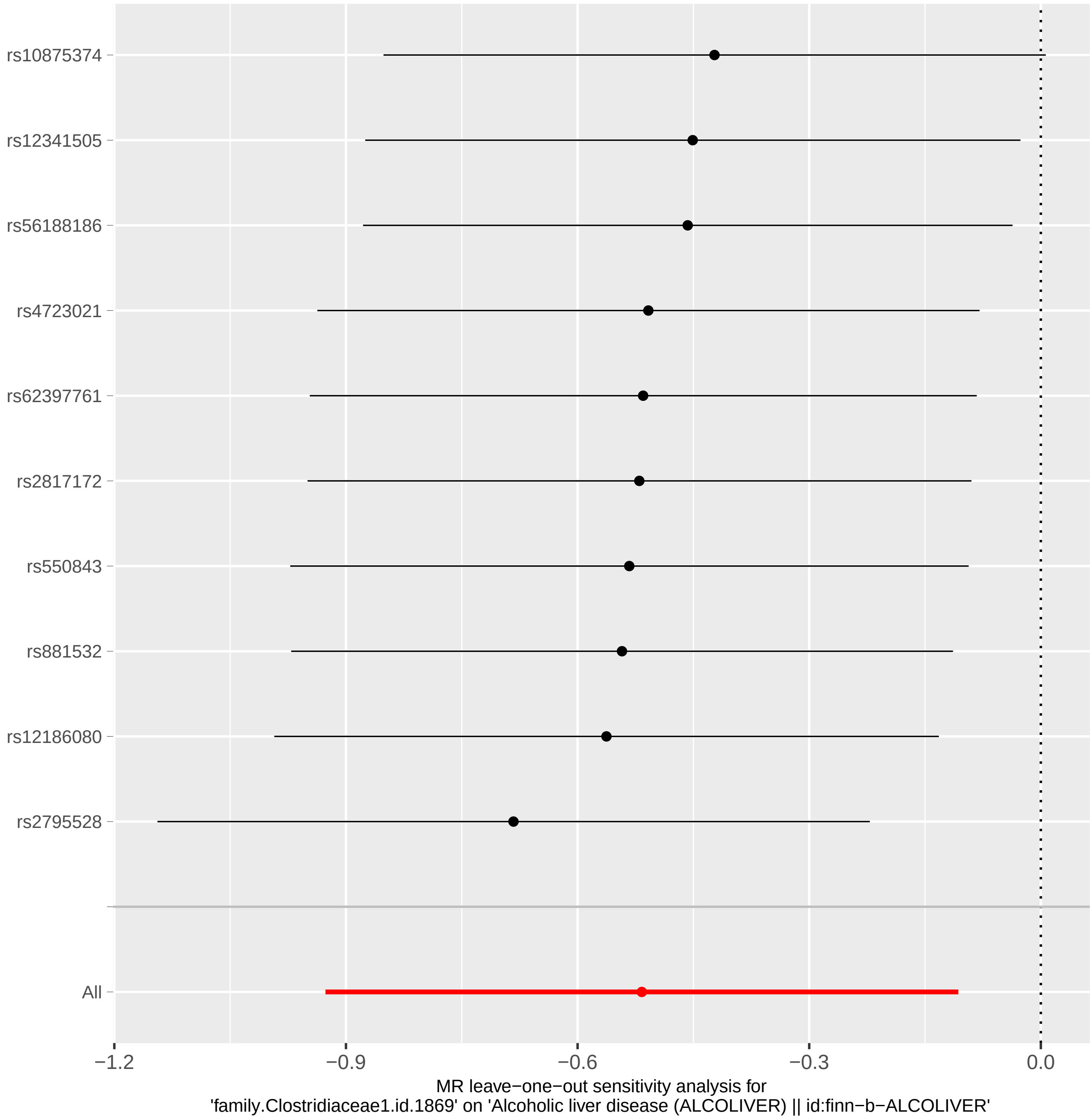

rs10875374
rs12341505
rs56188186
rs4723021
rs62397761
rs2817172
rs550843
rs881532
rs12186080
rs2795528
All
−1.2
−0.9
−0.6
−0.3
0.0
MR leave−one−out sensitivity analysis for
'family.Clostridiaceae1.id.1869' on 'Alcoholic liver disease (ALCOLIVER) || id:finn−b−ALCOLIVER'
