# Supplementary material for: Causal impact of gut microbiota on five liver diseases: insights from mendelian randomization and single-cell RNA sequencing
Source: Front Genet. 2024 Nov 11;15:1362139. doi: 10.3389/fgene.2024.1362139 (PMC11586359; doi:10.3389/fgene.2024.1362139)
Supplement: Supplementary file 1 [file DataSheet1.zip › Annex 1 _Data/MR results/Alcoholic liver disease/Alcoholic liver disease -figures/LeaveOne_finn-b-ALCOLIVER_class.Bacteroidia.id.912.pdf]

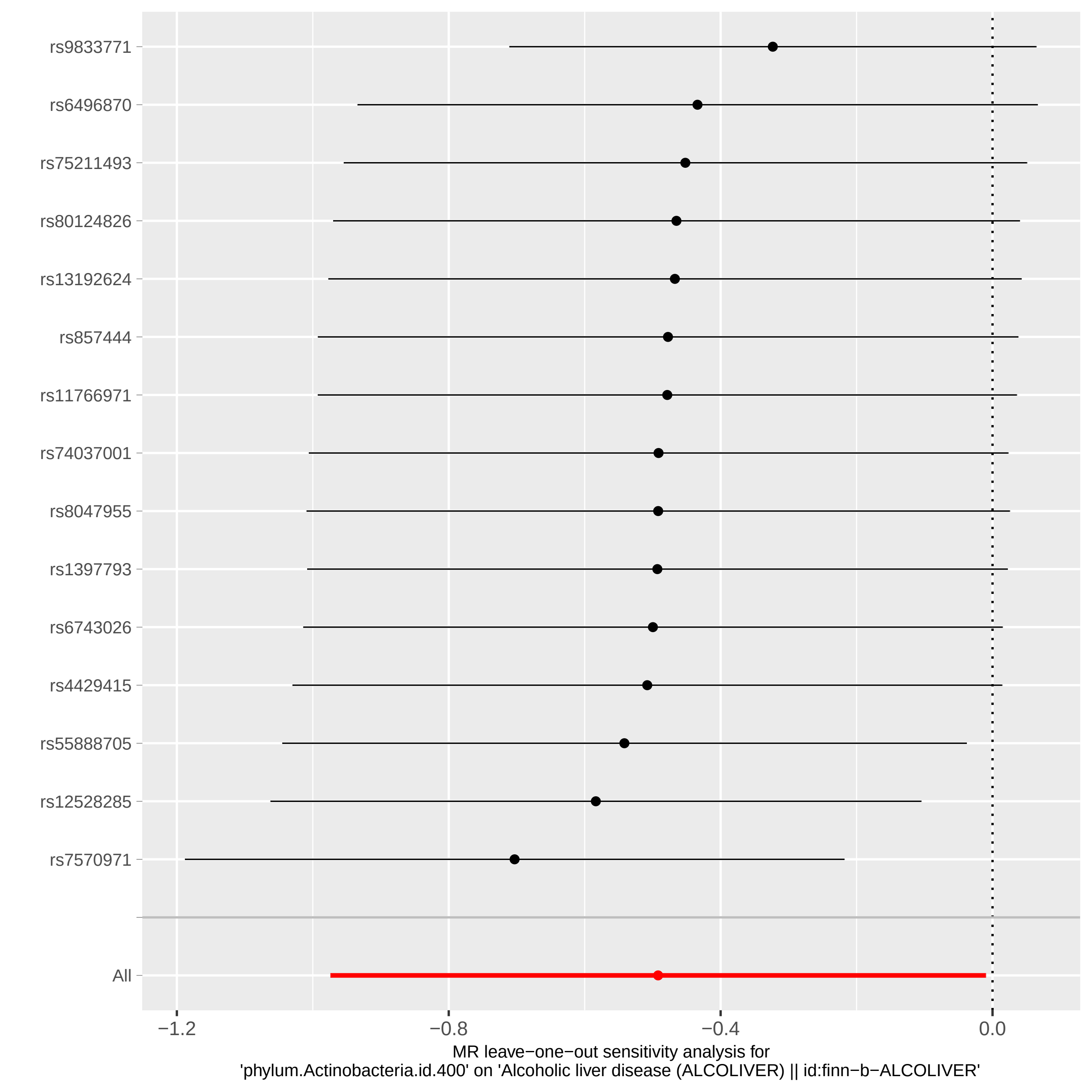
rs9833771
rs6496870
rs75211493
rs80124826
rs13192624
rs857444
rs11766971
rs74037001
rs8047955
rs1397793
rs6743026
rs4429415
rs55888705
rs12528285
rs7570971
All
−1.2
−0.8
−0.4
0.0
MR leave−one−out sensitivity analysis for
'phylum.Actinobacteria.id.400' on 'Alcoholic liver disease (ALCOLIVER) || id:finn−b−ALCOLIVER'
