# Supplementary material for: Causal impact of gut microbiota on five liver diseases: insights from mendelian randomization and single-cell RNA sequencing
Source: Front Genet. 2024 Nov 11;15:1362139. doi: 10.3389/fgene.2024.1362139 (PMC11586359; doi:10.3389/fgene.2024.1362139)
Supplement: Supplementary file 1 [file DataSheet1.zip › Annex 1 _Data/MR results/Alcoholic liver disease/Alcoholic liver disease -figures/LeaveOne_finn-b-ALCOLIVER_class.Coriobacteriia.id.809.pdf]

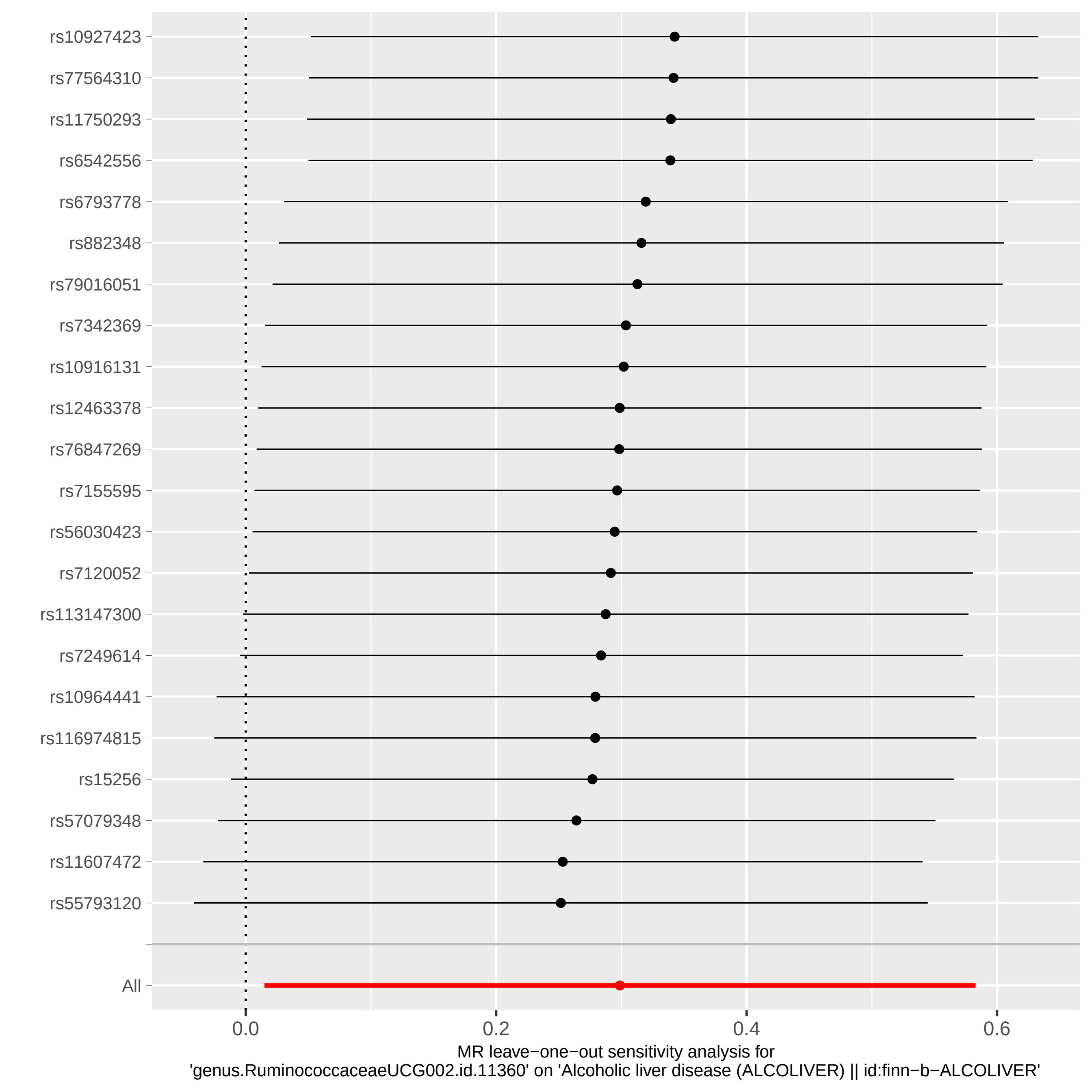
rs10927423
rs77564310
rs11750293
rs6542556
rs6793778
rs882348
rs79016051
rs7342369
rs10916131
rs12463378
rs76847269
rs7155595
rs56030423
rs7120052
rs113147300
rs7249614
rs10964441
rs116974815
rs15256
rs57079348
rs11607472
rs55793120
All
0.0
0.2
0.4
0.6
MR leave−one−out sensitivity analysis for
'genus.RuminococcaceaeUCG002.id.11360' on 'Alcoholic liver disease (ALCOLIVER) || id:finn−b−ALCOLIVER'
