# Supplementary material for: Causal impact of gut microbiota on five liver diseases: insights from mendelian randomization and single-cell RNA sequencing
Source: Front Genet. 2024 Nov 11;15:1362139. doi: 10.3389/fgene.2024.1362139 (PMC11586359; doi:10.3389/fgene.2024.1362139)
Supplement: Supplementary file 1 [file DataSheet1.zip › Annex 1 _Data/MR results/Alcoholic liver disease/Alcoholic liver disease -figures/LeaveOne_finn-b-ALCOLIVER_class.Deltaproteobacteria.id.3087.pdf]

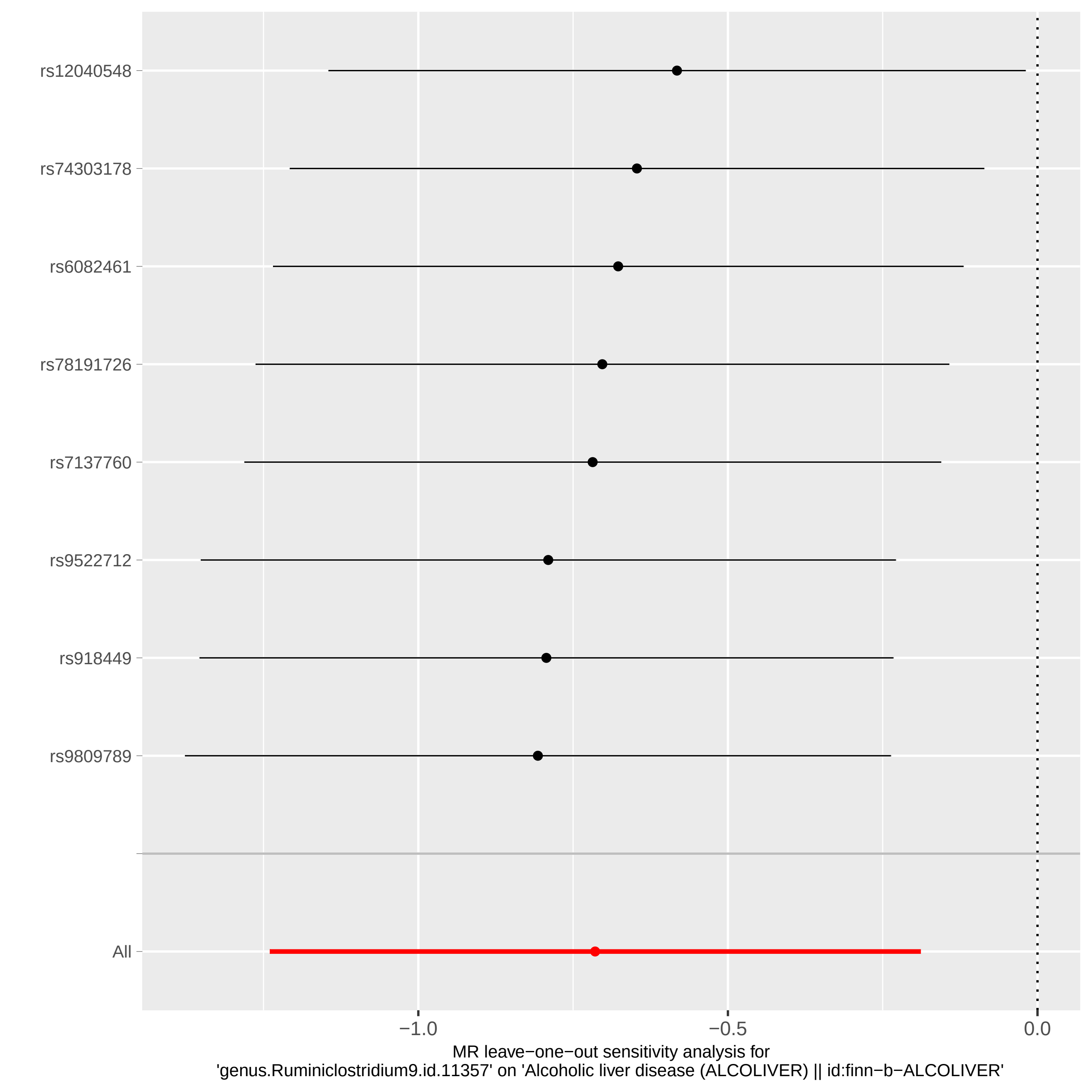

rs12040548
rs74303178
rs6082461
rs78191726
rs7137760
rs9522712
rs918449
rs9809789
All
−1.0
−0.5
0.0
MR leave−one−out sensitivity analysis for
'genus.Ruminiclostridium9.id.11357' on 'Alcoholic liver disease (ALCOLIVER) || id:finn−b−ALCOLIVER'
