# Supplementary material for: Causal impact of gut microbiota on five liver diseases: insights from mendelian randomization and single-cell RNA sequencing
Source: Front Genet. 2024 Nov 11;15:1362139. doi: 10.3389/fgene.2024.1362139 (PMC11586359; doi:10.3389/fgene.2024.1362139)
Supplement: Supplementary file 1 [file DataSheet1.zip › Annex 1 _Data/MR results/Alcoholic liver disease/Alcoholic liver disease -figures/ScatterPlot_finn-b-ALCOLIVER_class.Betaproteobacteria.id.2867.pdf]

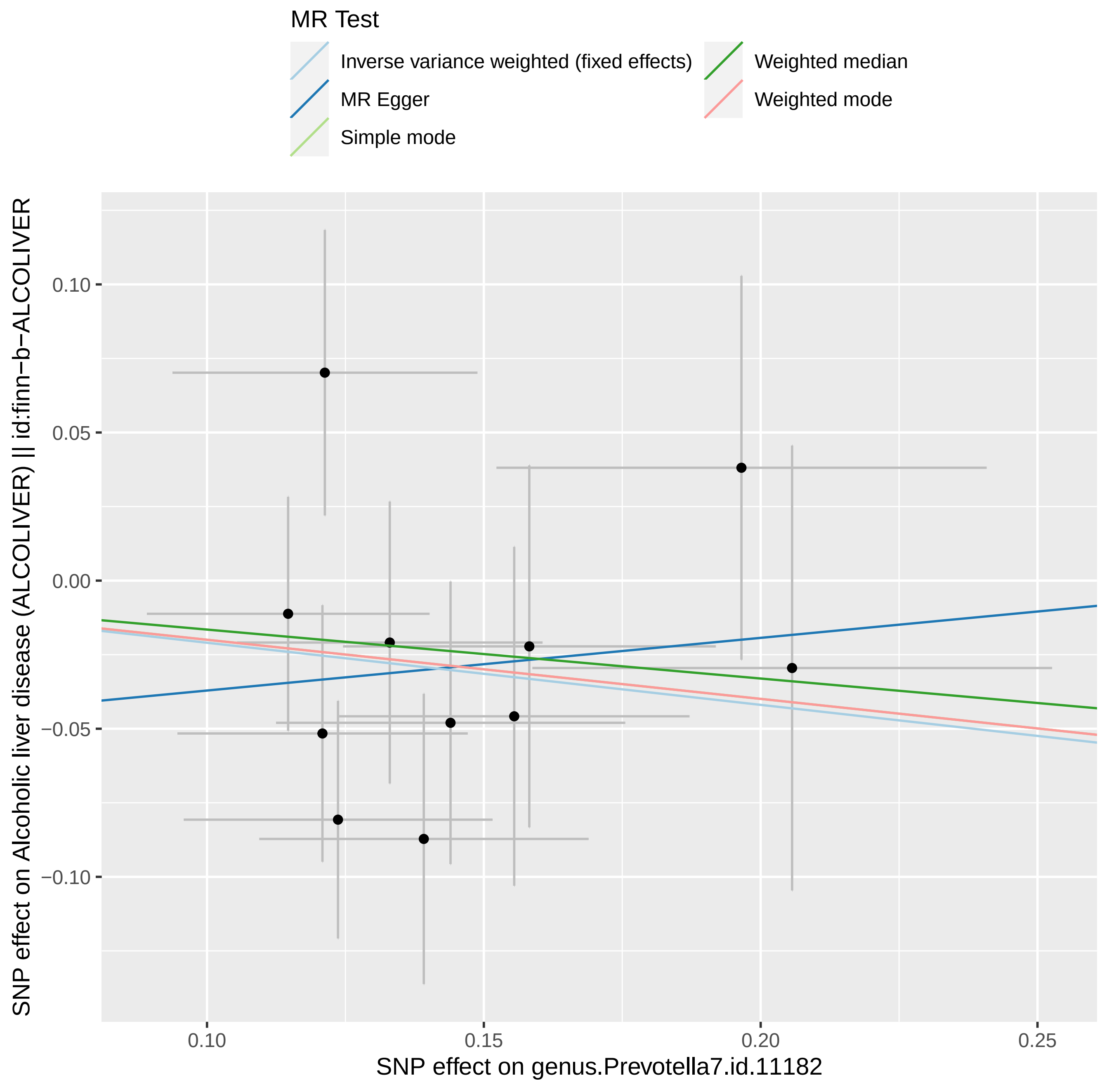

MR Test
Inverse variance weighted (fixed effects)
MR Egger
Simple mode
Weighted median
Weighted mode
0.10
0.05
0.00
−0.05
−0.10
0.10
0.15
0.20
0.25
SNP effect on Alcoholic liver disease (ALCOLIVER) || id:finn−b−ALCOLIVER
SNP effect on genus.Prevotella7.id.11182
