# Supplementary material for: Causal impact of gut microbiota on five liver diseases: insights from mendelian randomization and single-cell RNA sequencing
Source: Front Genet. 2024 Nov 11;15:1362139. doi: 10.3389/fgene.2024.1362139 (PMC11586359; doi:10.3389/fgene.2024.1362139)
Supplement: Supplementary file 1 [file DataSheet1.zip › Annex 1 _Data/MR results/Benign neoplasm/Benign neoplasm-figures/Benign neoplasm-Forest plot.pdf]

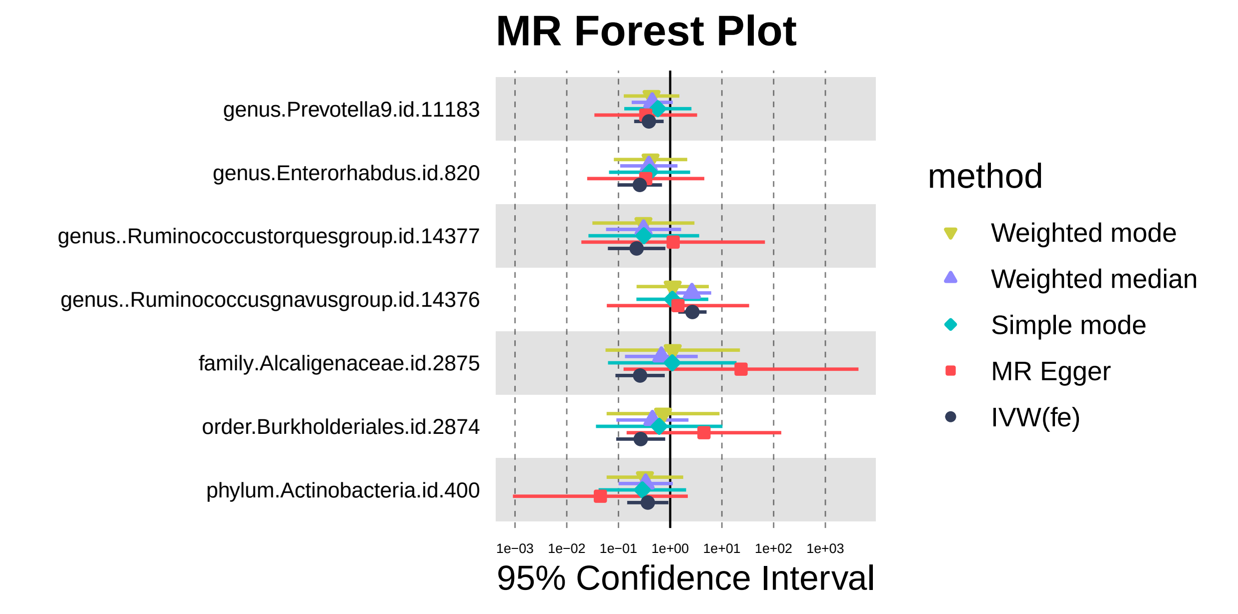
MR Forest Plot
genus.Prevotella9.id.11183
genus.Enterorhabdus.id.820
genus..Ruminococcustorquesgroup.id.14377
genus..Ruminococcusgnavusgroup.id.14376
family.Alcaligenaceae.id.2875
order.Burkholderiales.id.2874
phylum.Actinobacteria.id.400
1e−03
1e−02
1e−01
1e+00
1e+01
1e+02
1e+03
95% Confidence Interval
method
Weighted mode
Weighted median
Simple mode
MR Egger
IVW(fe)
