# Supplementary material for: Causal impact of gut microbiota on five liver diseases: insights from mendelian randomization and single-cell RNA sequencing
Source: Front Genet. 2024 Nov 11;15:1362139. doi: 10.3389/fgene.2024.1362139 (PMC11586359; doi:10.3389/fgene.2024.1362139)
Supplement: Supplementary file 1 [file DataSheet1.zip › Annex 1 _Data/MR results/Benign neoplasm/Benign neoplasm-figures/LeaveOne_finn-b-CD2_BENIGN_LIVER_class.Alphaproteobacteria.id.2379.pdf]

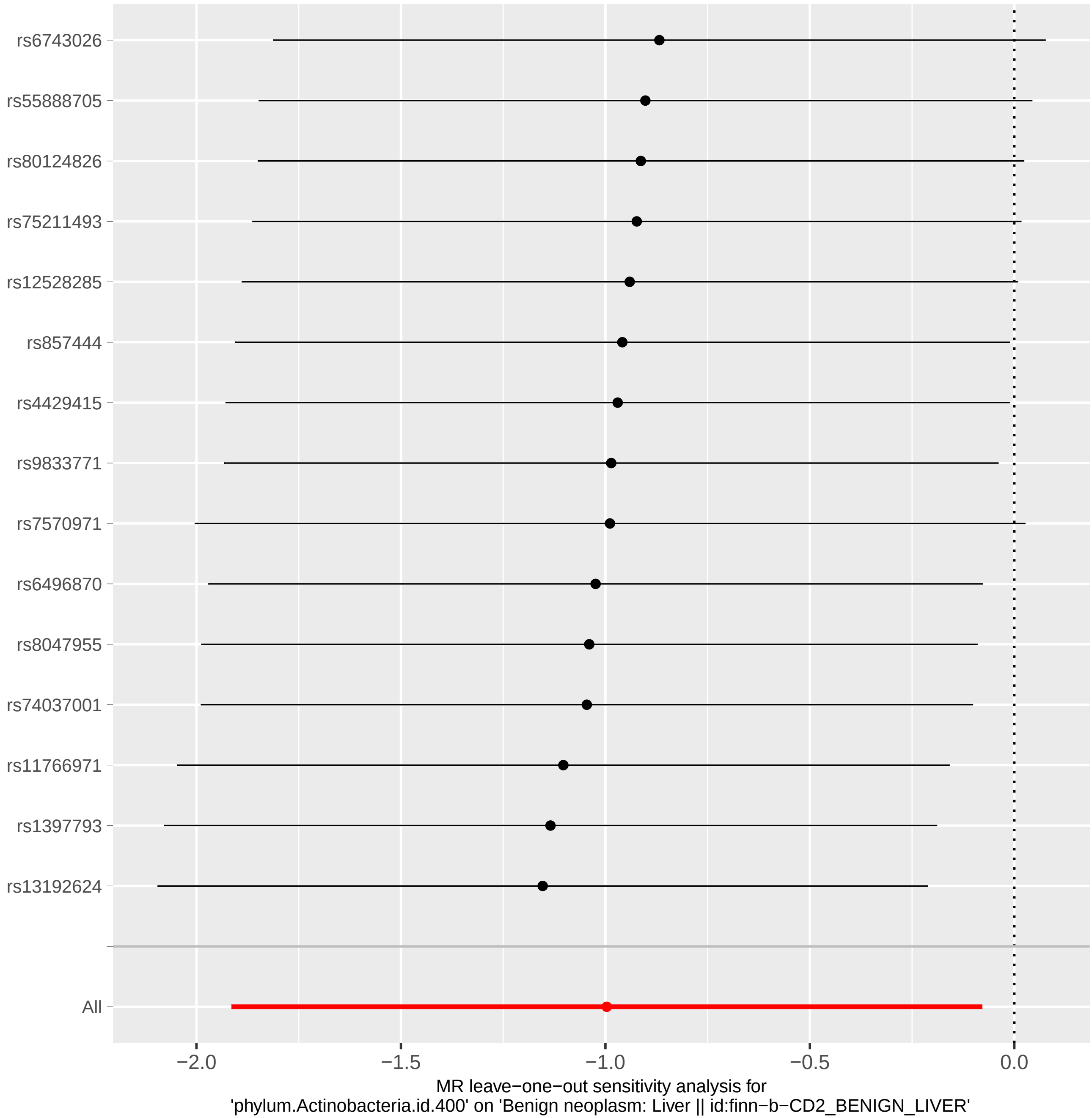
rs6743026
rs55888705
rs80124826
rs75211493
rs12528285
rs857444
rs4429415
rs9833771
rs7570971
rs6496870
rs8047955
rs74037001
rs11766971
rs1397793
rs13192624
All
−2.0
−1.5
−1.0
−0.5
0.0
MR leave−one−out sensitivity analysis for
'phylum.Actinobacteria.id.400' on 'Benign neoplasm: Liver || id:finn−b−CD2_BENIGN_LIVER'
