# Supplementary material for: Causal impact of gut microbiota on five liver diseases: insights from mendelian randomization and single-cell RNA sequencing
Source: Front Genet. 2024 Nov 11;15:1362139. doi: 10.3389/fgene.2024.1362139 (PMC11586359; doi:10.3389/fgene.2024.1362139)
Supplement: Supplementary file 1 [file DataSheet1.zip › Annex 1 _Data/MR results/Benign neoplasm/Benign neoplasm-figures/LeaveOne_finn-b-CD2_BENIGN_LIVER_class.Bacilli.id.1673.pdf]

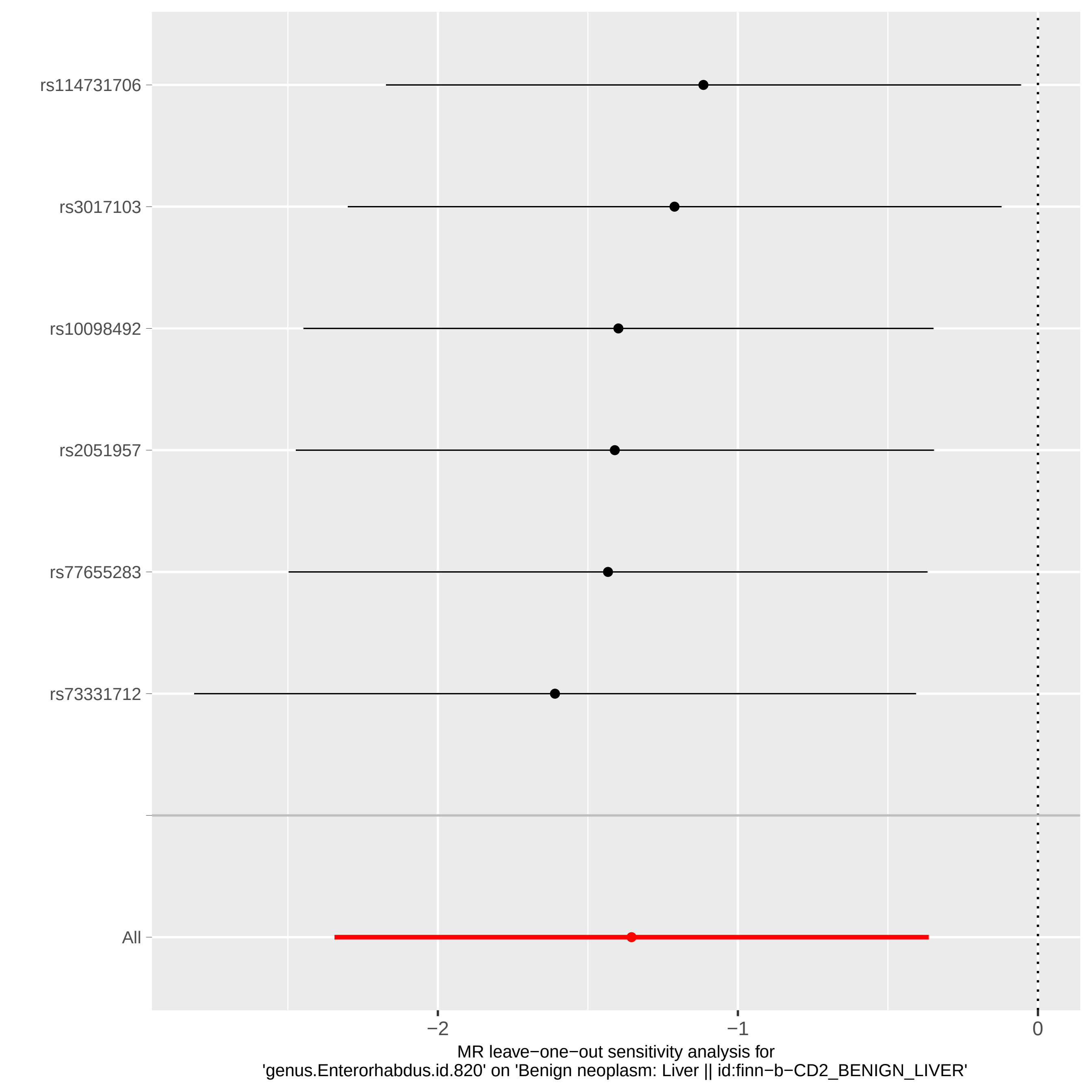
rs114731706
rs3017103
rs10098492
rs2051957
rs77655283
rs73331712
All
−2
−1
0
MR leave−one−out sensitivity analysis for
'genus.Enterorhabdus.id.820' on 'Benign neoplasm: Liver || id:finn−b−CD2_BENIGN_LIVER'
