# Supplementary material for: Causal impact of gut microbiota on five liver diseases: insights from mendelian randomization and single-cell RNA sequencing
Source: Front Genet. 2024 Nov 11;15:1362139. doi: 10.3389/fgene.2024.1362139 (PMC11586359; doi:10.3389/fgene.2024.1362139)
Supplement: Supplementary file 1 [file DataSheet1.zip › Annex 1 _Data/MR results/Benign neoplasm/Benign neoplasm-figures/LeaveOne_finn-b-CD2_BENIGN_LIVER_class.Betaproteobacteria.id.2867.pdf]

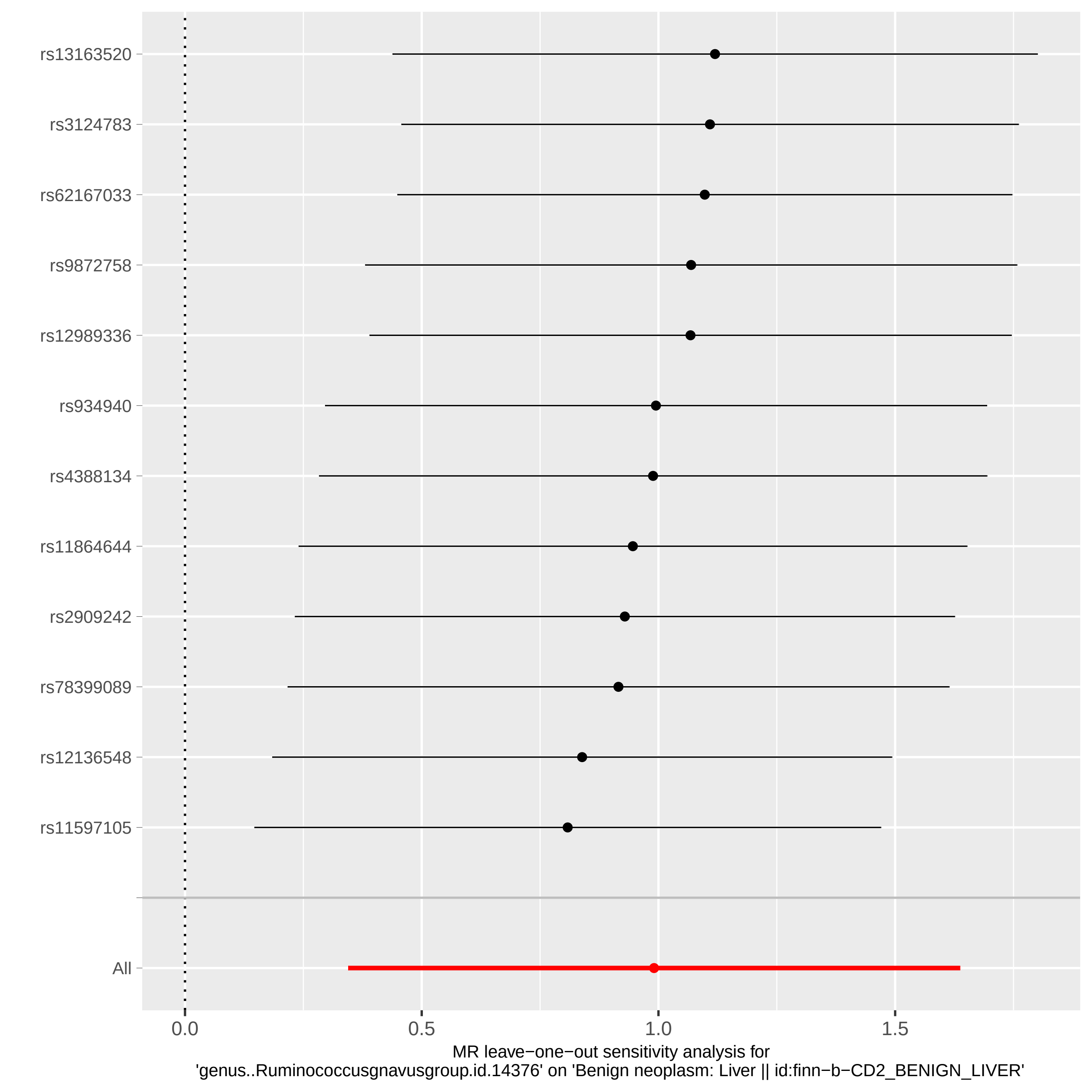
rs13163520
rs3124783
rs62167033
rs9872758
rs12989336
rs934940
rs4388134
rs11864644
rs2909242
rs78399089
rs12136548
rs11597105
All
0.0
0.5
1.0
1.5
MR leave−one−out sensitivity analysis for
'genus..Ruminococcusgnavusgroup.id.14376' on 'Benign neoplasm: Liver || id:finn−b−CD2_BENIGN_LIVER'
