# Supplementary material for: Causal impact of gut microbiota on five liver diseases: insights from mendelian randomization and single-cell RNA sequencing
Source: Front Genet. 2024 Nov 11;15:1362139. doi: 10.3389/fgene.2024.1362139 (PMC11586359; doi:10.3389/fgene.2024.1362139)
Supplement: Supplementary file 1 [file DataSheet1.zip › Annex 1 _Data/MR results/Benign neoplasm/Benign neoplasm-figures/ScatterPlot_finn-b-CD2_BENIGN_LIVER_class.Bacilli.id.1673.pdf]

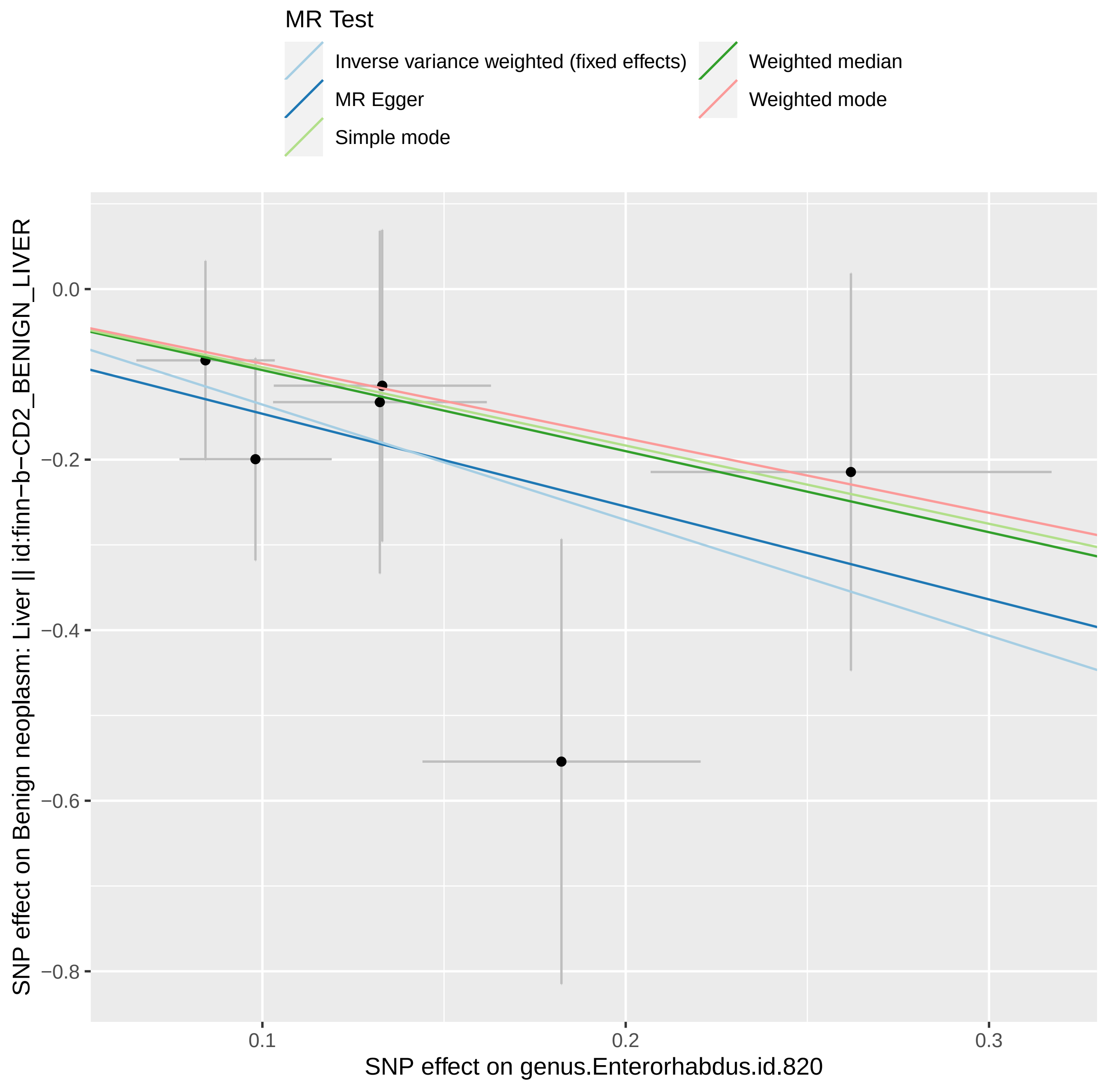
MR Test
Inverse variance weighted (fixed effects)
MR Egger
Simple mode
Weighted median
Weighted mode
SNP effect on Benign neoplasm: Liver || id:finn−b−CD2_BENIGN_LIVER
0.0
−0.2
−0.4
−0.6
−0.8
0.1
0.2
0.3
SNP effect on genus.Enterorhabdus.id.820
