# Supplementary material for: Causal impact of gut microbiota on five liver diseases: insights from mendelian randomization and single-cell RNA sequencing
Source: Front Genet. 2024 Nov 11;15:1362139. doi: 10.3389/fgene.2024.1362139 (PMC11586359; doi:10.3389/fgene.2024.1362139)
Supplement: Supplementary file 1 [file DataSheet1.zip › Annex 1 _Data/MR results/Cirrhosis/Cirrhosis-figure/LeaveOne_ebi-a-GCST90018826_class.Betaproteobacteria.id.2867.pdf]

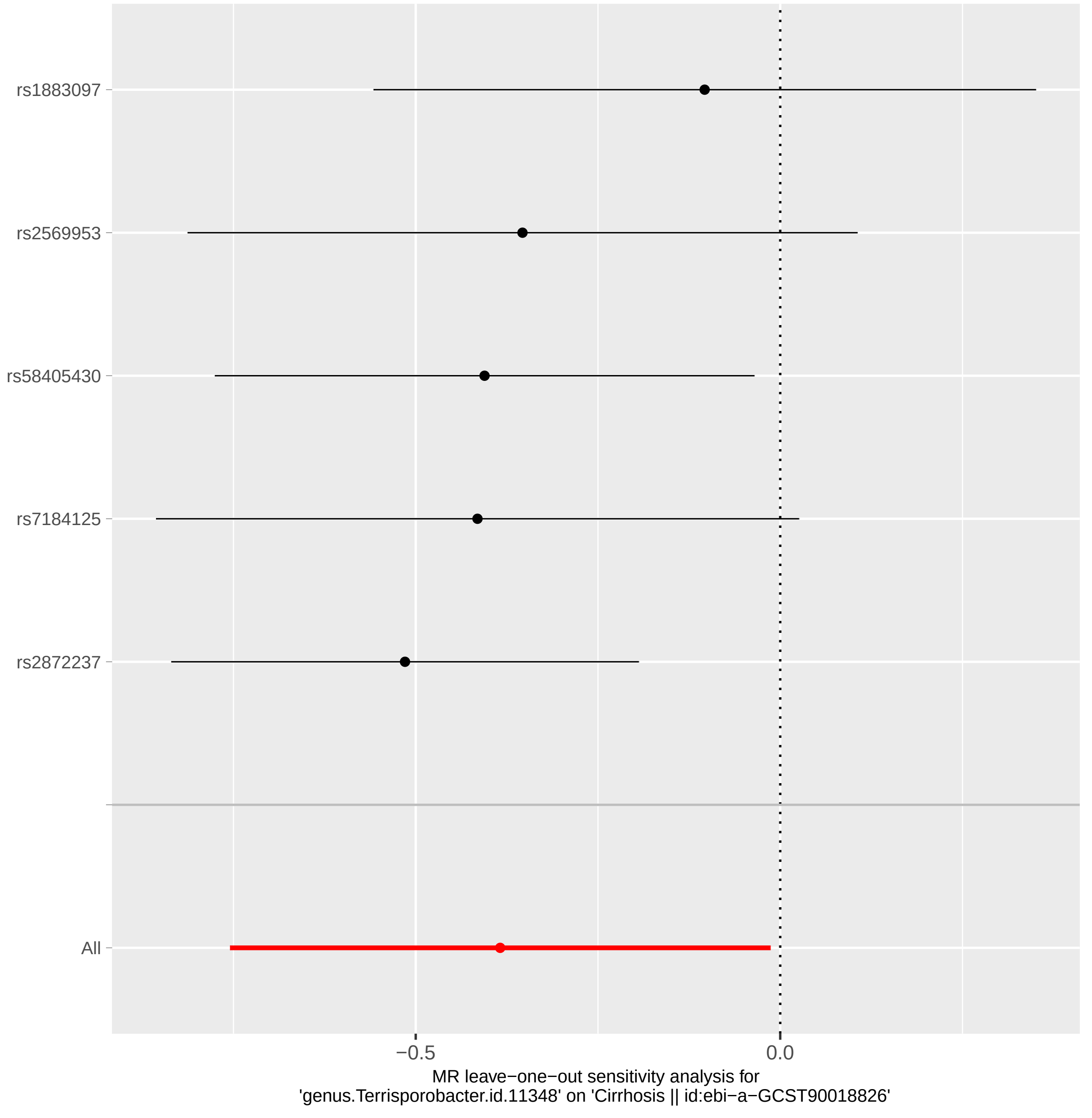

rs1883097
rs2569953
rs58405430
rs7184125
rs2872237
All
−0.5
0.0
MR leave−one−out sensitivity analysis for
'genus.Terrisporobacter.id.11348' on 'Cirrhosis || id:ebi−a−GCST90018826'
