# Supplementary material for: Causal impact of gut microbiota on five liver diseases: insights from mendelian randomization and single-cell RNA sequencing
Source: Front Genet. 2024 Nov 11;15:1362139. doi: 10.3389/fgene.2024.1362139 (PMC11586359; doi:10.3389/fgene.2024.1362139)
Supplement: Supplementary file 1 [file DataSheet1.zip › Annex 1 _Data/MR results/Cirrhosis/Cirrhosis-figure/LeaveOne_ebi-a-GCST90018826_class.Clostridia.id.1859.pdf]

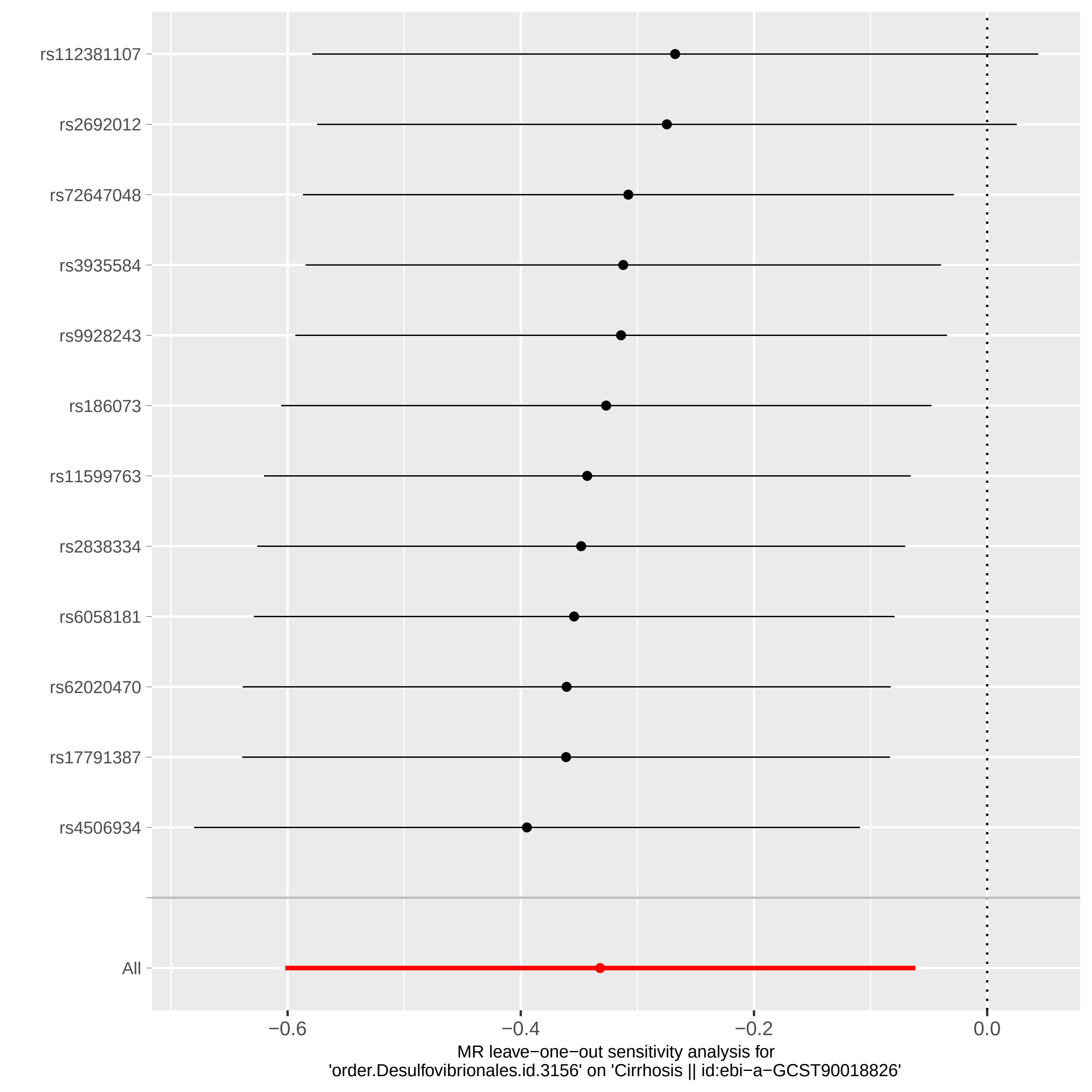
rs112381107
rs2692012
rs72647048
rs3935584
rs9928243
rs186073
rs11599763
rs2838334
rs6058181
rs62020470
rs17791387
rs4506934
All
−0.6
−0.4
−0.2
0.0
MR leave−one−out sensitivity analysis for
'order.Desulfovibrionales.id.3156' on 'Cirrhosis || id:ebi−a−GCST90018826'
