# Supplementary material for: Causal impact of gut microbiota on five liver diseases: insights from mendelian randomization and single-cell RNA sequencing
Source: Front Genet. 2024 Nov 11;15:1362139. doi: 10.3389/fgene.2024.1362139 (PMC11586359; doi:10.3389/fgene.2024.1362139)
Supplement: Supplementary file 1 [file DataSheet1.zip › Annex 1 _Data/MR results/Hepatic failure, not elsewhere classified/Hepatic failure, not elsewhere classified-figures/LeaveOne_finn-b-K11_HEPFAIL_class.Alphaproteobacteria.id.2379.pdf]

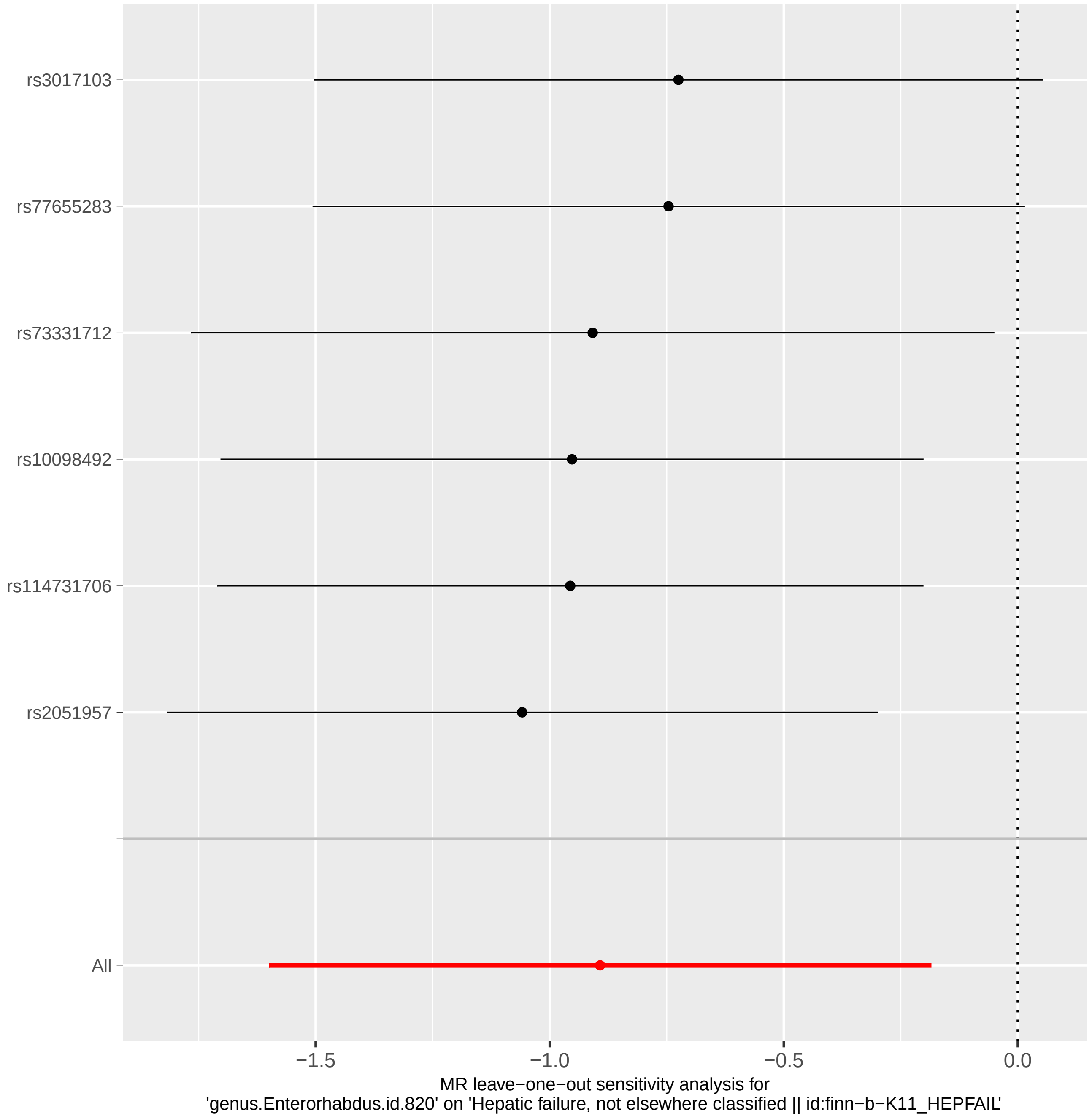

rs3017103
rs77655283
rs73331712
rs10098492
rs114731706
rs2051957
All
−1.5
−1.0
−0.5
0.0
MR leave−one−out sensitivity analysis for
'genus.Enterorhabdus.id.820' on 'Hepatic failure, not elsewhere classified || id:finn−b−K11_HEPFAIL'
