# Supplementary material for: Causal impact of gut microbiota on five liver diseases: insights from mendelian randomization and single-cell RNA sequencing
Source: Front Genet. 2024 Nov 11;15:1362139. doi: 10.3389/fgene.2024.1362139 (PMC11586359; doi:10.3389/fgene.2024.1362139)
Supplement: Supplementary file 1 [file DataSheet1.zip › Annex 1 _Data/MR results/Hepatic failure, not elsewhere classified/Hepatic failure, not elsewhere classified-figures/LeaveOne_finn-b-K11_HEPFAIL_class.Betaproteobacteria.id.2867.pdf]

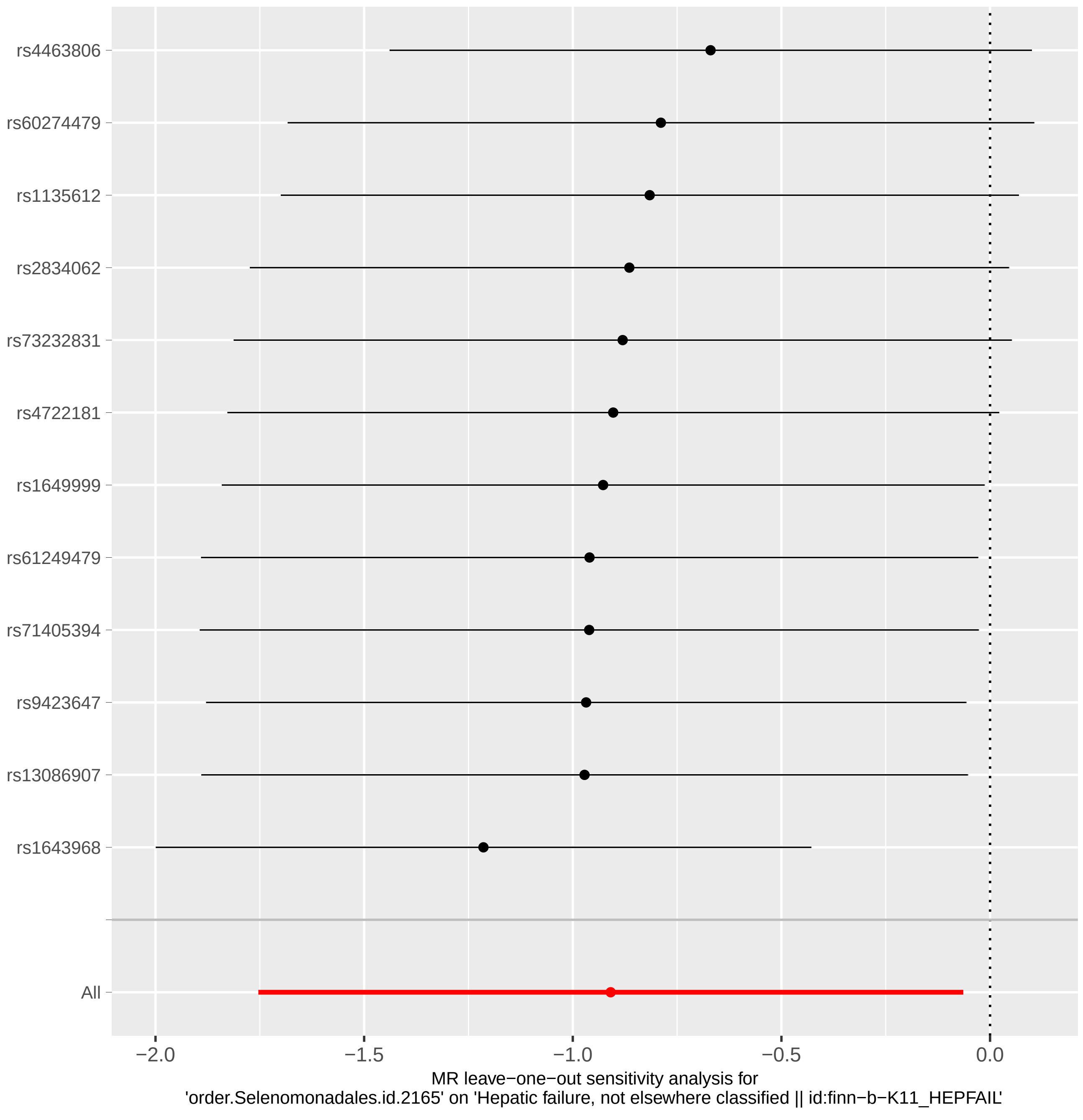
rs4463806
rs60274479
rs1135612
rs2834062
rs73232831
rs4722181
rs1649999
rs61249479
rs71405394
rs9423647
rs13086907
rs1643968
All
−2.0
−1.5
−1.0
−0.5
0.0
MR leave−one−out sensitivity analysis for
'order.Selenomonadales.id.2165' on 'Hepatic failure, not elsewhere classified || id:finn−b−K11_HEPFAIL'
