# Supplementary material for: Causal impact of gut microbiota on five liver diseases: insights from mendelian randomization and single-cell RNA sequencing
Source: Front Genet. 2024 Nov 11;15:1362139. doi: 10.3389/fgene.2024.1362139 (PMC11586359; doi:10.3389/fgene.2024.1362139)
Supplement: Supplementary file 1 [file DataSheet1.zip › Annex 1 _Data/MR results/Hepatic failure, not elsewhere classified/Hepatic failure, not elsewhere classified-figures/ScatterPlot_finn-b-K11_HEPFAIL_class.Bacteroidia.id.912.pdf]

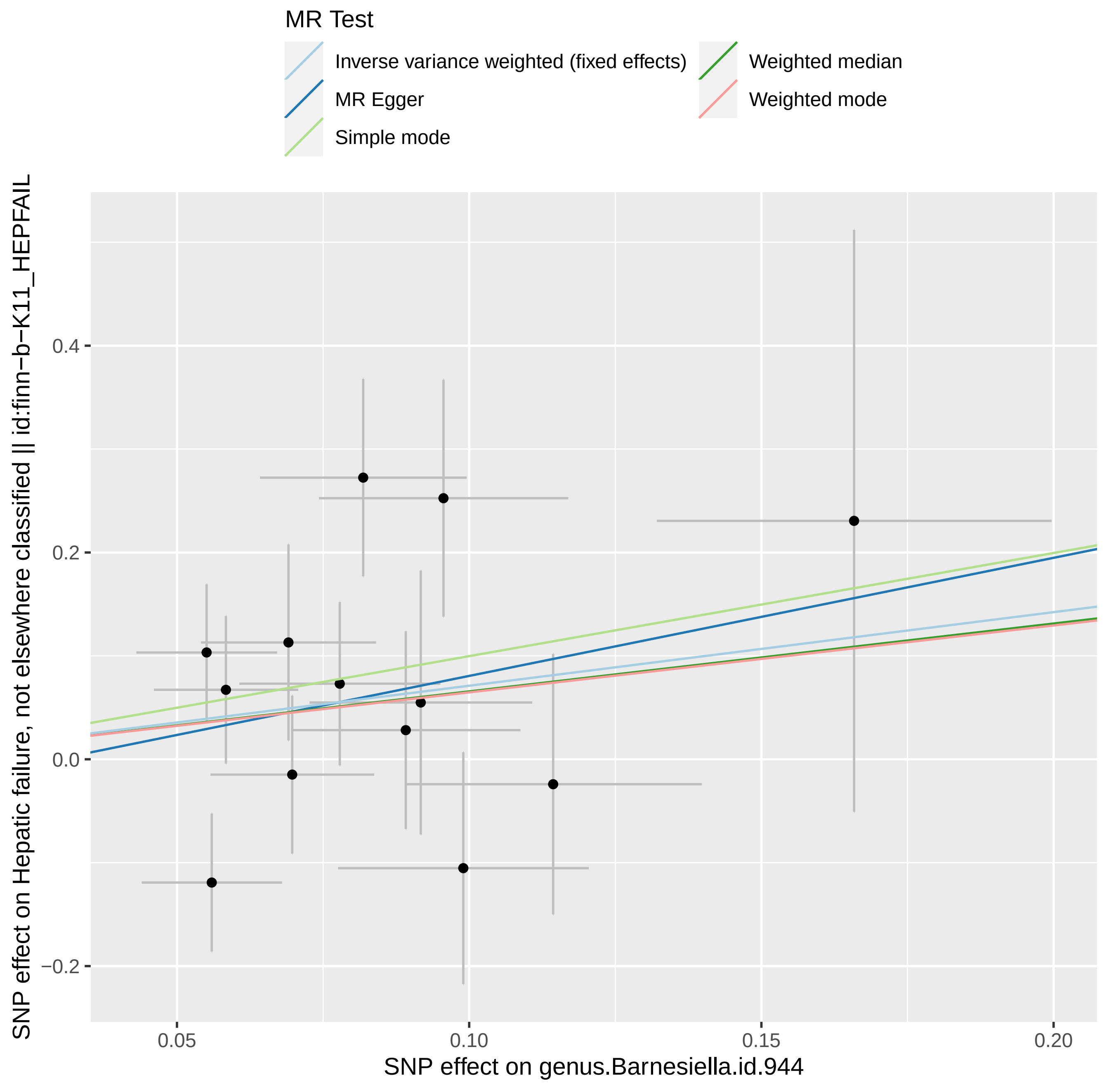
MR Test
Inverse variance weighted (fixed effects)
MR Egger
Simple mode
Weighted median
Weighted mode
SNP effect on Hepatic failure, not elsewhere classified || id:finn−b−K11_HEPFAIL
0.4
0.2
0.0
−0.2
0.05
0.10
0.15
0.20
SNP effect on genus.Barnesiella.id.944
