# Supplementary material for: Causal impact of gut microbiota on five liver diseases: insights from mendelian randomization and single-cell RNA sequencing
Source: Front Genet. 2024 Nov 11;15:1362139. doi: 10.3389/fgene.2024.1362139 (PMC11586359; doi:10.3389/fgene.2024.1362139)
Supplement: Supplementary file 1 [file DataSheet1.zip › Annex 1 _Data/MR results/Malignant neoplasm of liver, primary/Malignant neoplasm of liver, primary-figures/LeaveOne_GCST90041812_class.Actinobacteria.id.419.pdf]

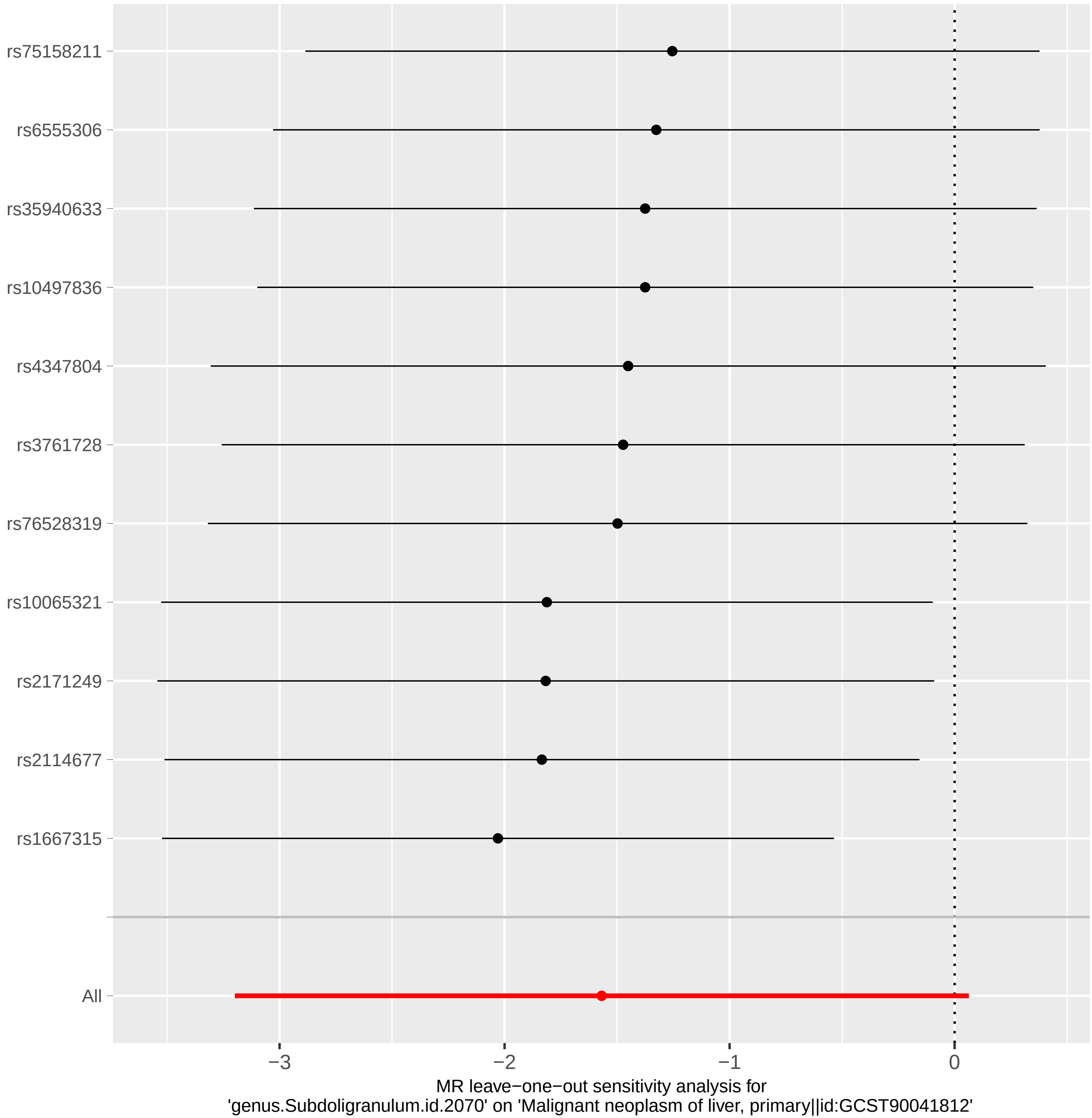

rs75158211
rs6555306
rs35940633
rs10497836
rs4347804
rs3761728
rs76528319
rs10065321
rs2171249
rs2114677
rs1667315
All
−3
−2
−1
0
MR leave−one−out sensitivity analysis for
'genus.Subdoligranulum.id.2070' on 'Malignant neoplasm of liver, primary||id:GCST90041812'
