# Supplementary material for: Causal impact of gut microbiota on five liver diseases: insights from mendelian randomization and single-cell RNA sequencing
Source: Front Genet. 2024 Nov 11;15:1362139. doi: 10.3389/fgene.2024.1362139 (PMC11586359; doi:10.3389/fgene.2024.1362139)
Supplement: Supplementary file 1 [file DataSheet1.zip › Annex 1 _Data/MR results/Malignant neoplasm of liver, primary/Malignant neoplasm of liver, primary-figures/LeaveOne_GCST90041812_class.Betaproteobacteria.id.2867.pdf]

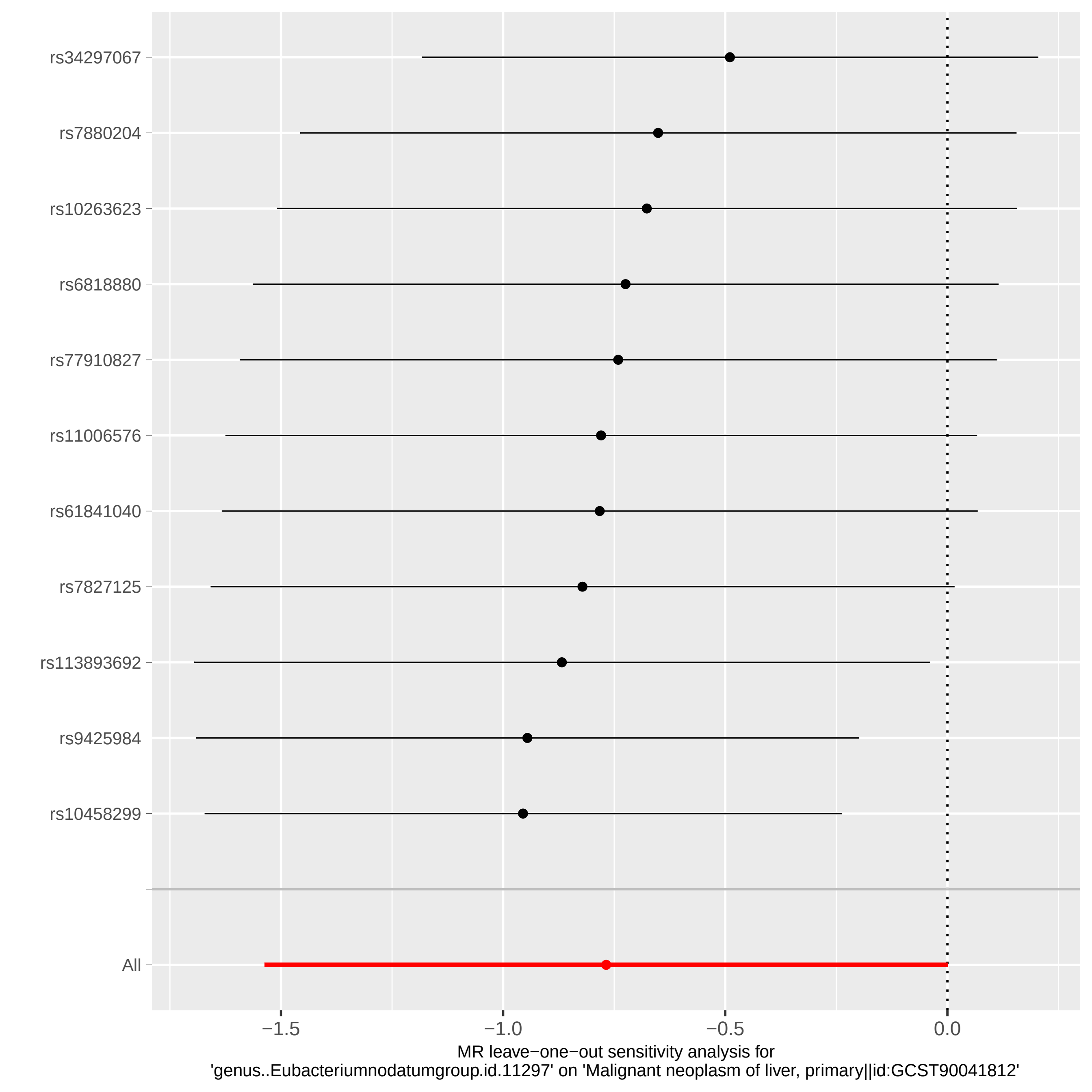
rs34297067
rs7880204
rs10263623
rs6818880
rs77910827
rs11006576
rs61841040
rs7827125
rs113893692
rs9425984
rs10458299
All
−1.5
−1.0
−0.5
0.0
MR leave−one−out sensitivity analysis for
'genus..Eubacteriumnodatumgroup.id.11297' on 'Malignant neoplasm of liver, primary||id:GCST90041812'
