# Supplementary material for: Causal impact of gut microbiota on five liver diseases: insights from mendelian randomization and single-cell RNA sequencing
Source: Front Genet. 2024 Nov 11;15:1362139. doi: 10.3389/fgene.2024.1362139 (PMC11586359; doi:10.3389/fgene.2024.1362139)
Supplement: Supplementary file 1 [file DataSheet1.zip › Annex 1 _Data/MR results/Malignant neoplasm of liver, primary/Malignant neoplasm of liver, primary-figures/LeaveOne_GCST90041812_class.Coriobacteriia.id.809.pdf]

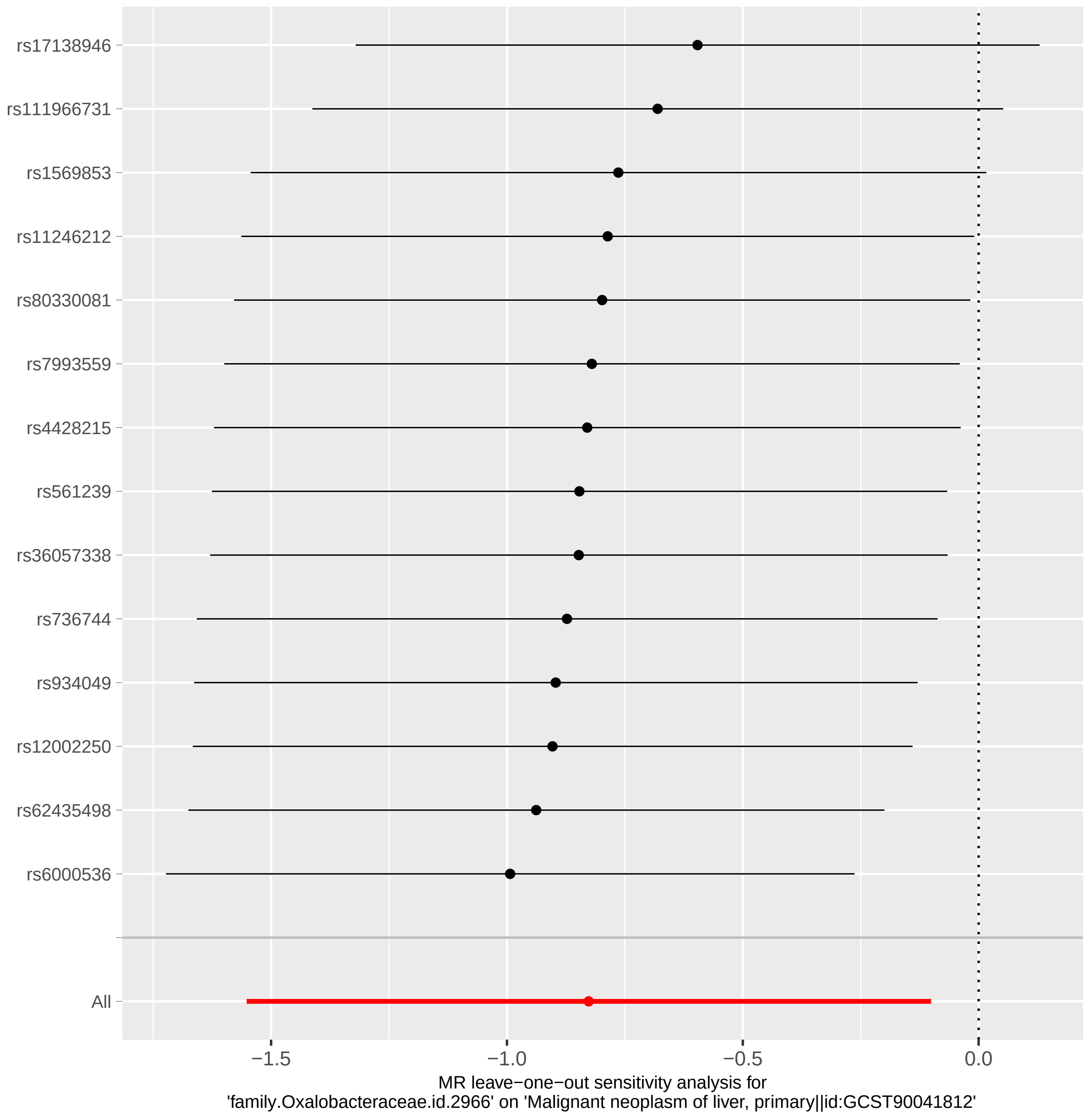
rs17138946
rs111966731
rs1569853
rs11246212
rs80330081
rs7993559
rs4428215
rs561239
rs36057338
rs736744
rs934049
rs12002250
rs62435498
rs6000536
All
−1.5
−1.0
−0.5
0.0
MR leave−one−out sensitivity analysis for
'family.Oxalobacteraceae.id.2966' on 'Malignant neoplasm of liver, primary||id:GCST90041812'
