# Supplementary material for: Causal impact of gut microbiota on five liver diseases: insights from mendelian randomization and single-cell RNA sequencing
Source: Front Genet. 2024 Nov 11;15:1362139. doi: 10.3389/fgene.2024.1362139 (PMC11586359; doi:10.3389/fgene.2024.1362139)
Supplement: Supplementary file 1 [file DataSheet1.zip › Annex 1 _Data/MR results/Malignant neoplasm of liver, primary/Malignant neoplasm of liver, primary-figures/LeaveOne_GCST90041812_class.Deltaproteobacteria.id.3087.pdf]

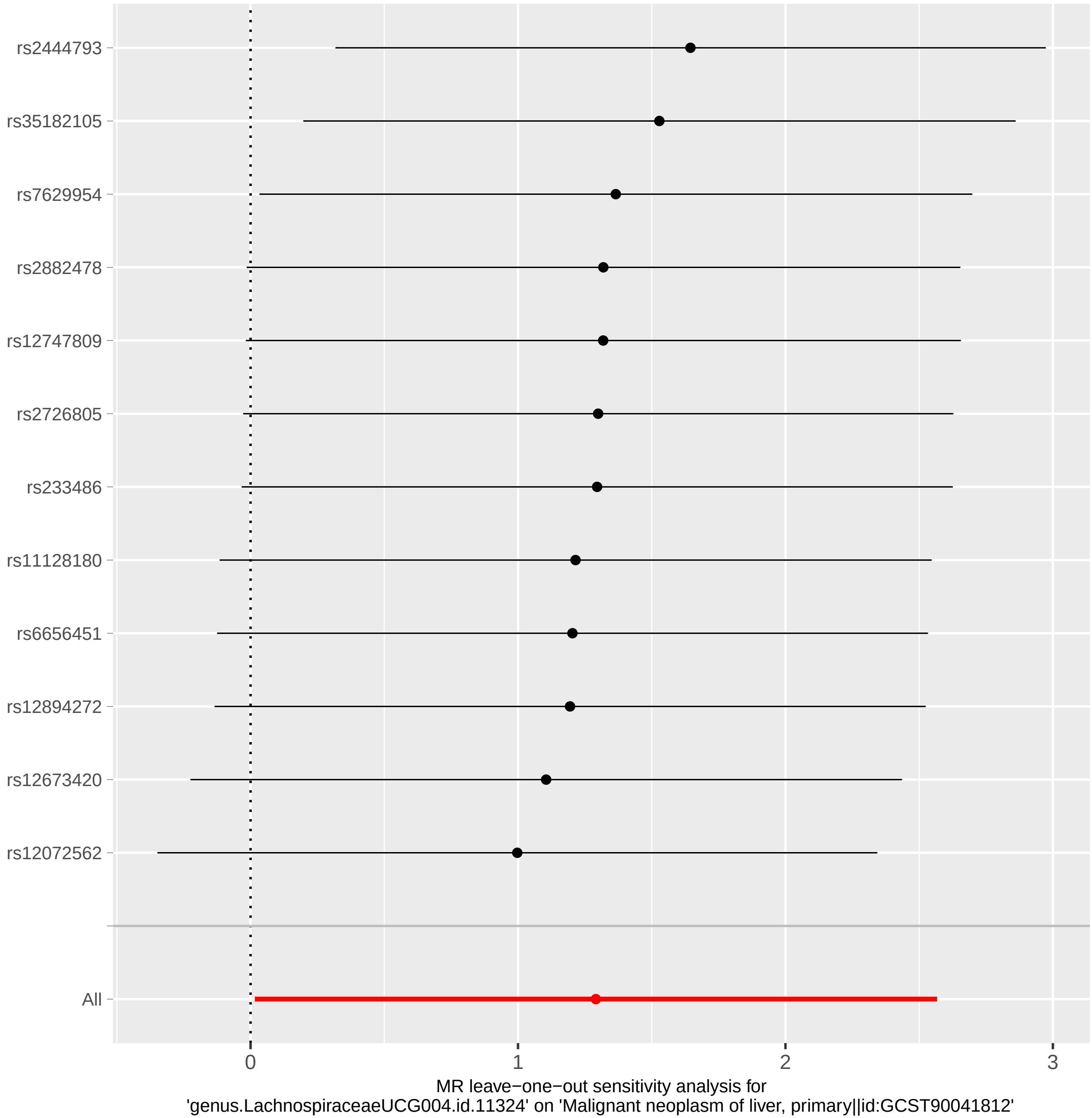

rs2444793
rs35182105
rs7629954
rs2882478
rs12747809
rs2726805
rs233486
rs11128180
rs6656451
rs12894272
rs12673420
rs12072562
All
0
1
2
3
MR leave−one−out sensitivity analysis for
'genus.LachnospiraceaeUCG004.id.11324' on 'Malignant neoplasm of liver, primary||id:GCST90041812'
