# Supplementary material for: Causal impact of gut microbiota on five liver diseases: insights from mendelian randomization and single-cell RNA sequencing
Source: Front Genet. 2024 Nov 11;15:1362139. doi: 10.3389/fgene.2024.1362139 (PMC11586359; doi:10.3389/fgene.2024.1362139)
Supplement: Supplementary file 1 [file DataSheet1.zip › Annex 1 _Data/MR results/Malignant neoplasm of liver, primary/Malignant neoplasm of liver, primary-figures/LeaveOne_GCST90041812_class.Erysipelotrichia.id.2147.pdf]

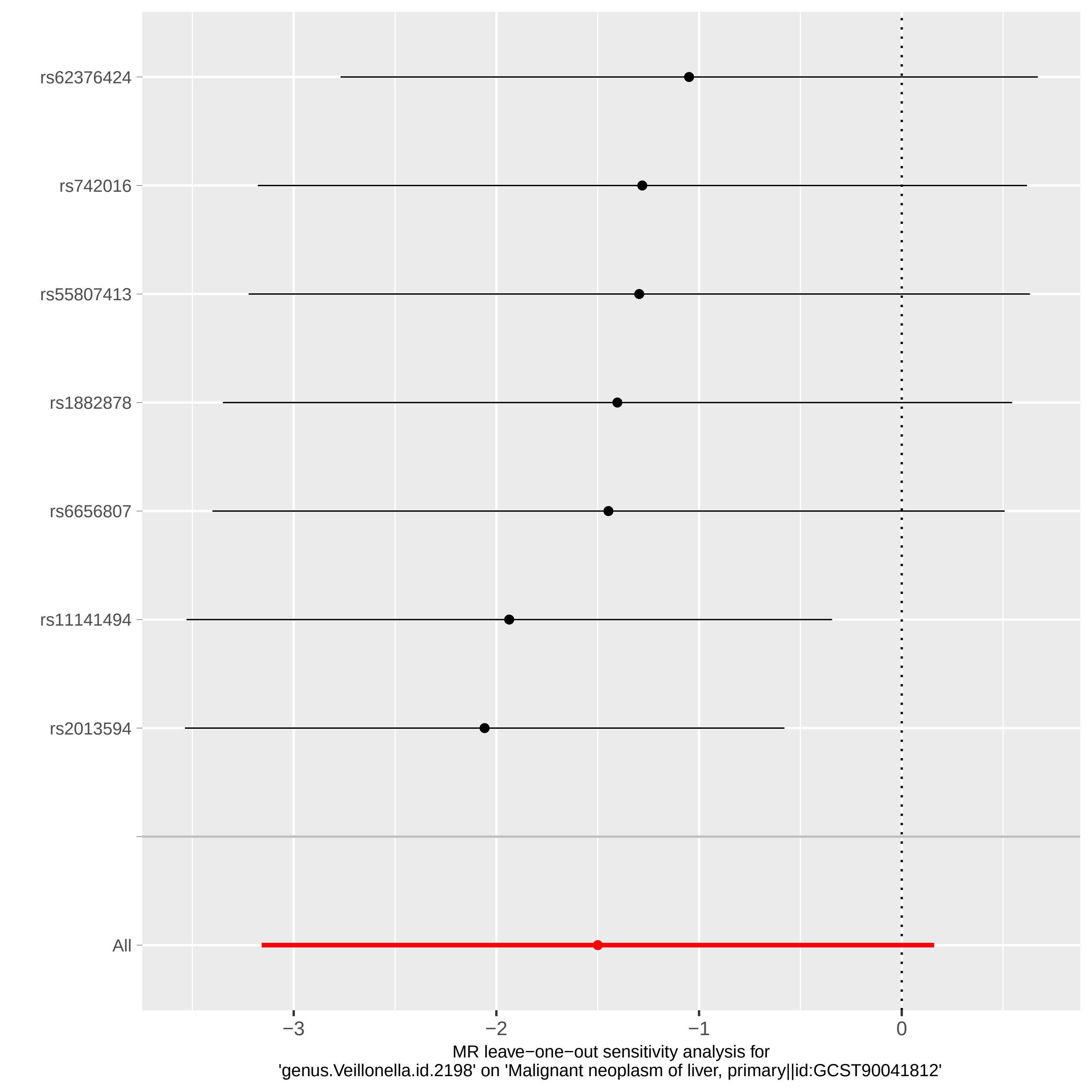

rs62376424
rs742016
rs55807413
rs1882878
rs6656807
rs11141494
rs2013594
All
−3
−2
−1
0
MR leave−one−out sensitivity analysis for
'genus.Veillonella.id.2198' on 'Malignant neoplasm of liver, primary||id:GCST90041812'
