# Supplementary material for: Causal impact of gut microbiota on five liver diseases: insights from mendelian randomization and single-cell RNA sequencing
Source: Front Genet. 2024 Nov 11;15:1362139. doi: 10.3389/fgene.2024.1362139 (PMC11586359; doi:10.3389/fgene.2024.1362139)
Supplement: Supplementary file 1 [file DataSheet1.zip › Annex 1 _Data/MR results/Malignant neoplasm of liver, primary/Malignant neoplasm of liver, primary-figures/Malignant neoplasm of liver, primary-Forest.pdf]

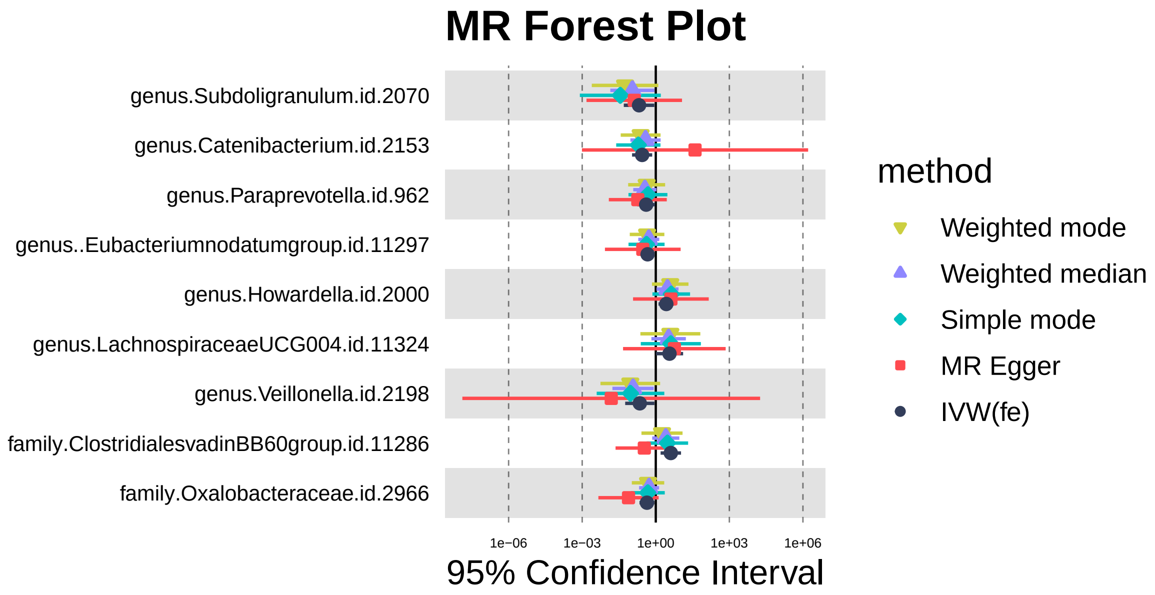
MR Forest Plot
genus.Subdoligranulum.id.2070
genus.Catenibacterium.id.2153
genus.Paraprevotella.id.962
genus..Eubacteriumnodatumgroup.id.11297
genus.Howardella.id.2000
genus.LachnospiraceaeUCG004.id.11324
genus.Veillonella.id.2198
family.ClostridialesvadinBB60group.id.11286
family.Oxalobacteraceae.id.2966
1e−06
1e−03
1e+00
1e+03
1e+06
95% Confidence Interval
method
Weighted mode
Weighted median
Simple mode
MR Egger
IVW(fe)
