# Supplementary material for: Causal impact of gut microbiota on five liver diseases: insights from mendelian randomization and single-cell RNA sequencing
Source: Front Genet. 2024 Nov 11;15:1362139. doi: 10.3389/fgene.2024.1362139 (PMC11586359; doi:10.3389/fgene.2024.1362139)
Supplement: Supplementary file 1 [file DataSheet1.zip › Annex 1 _Data/MR results/Malignant neoplasm of liver, primary/Malignant neoplasm of liver, primary-figures/ScatterPlot_GCST90041812_class.Erysipelotrichia.id.2147.pdf]

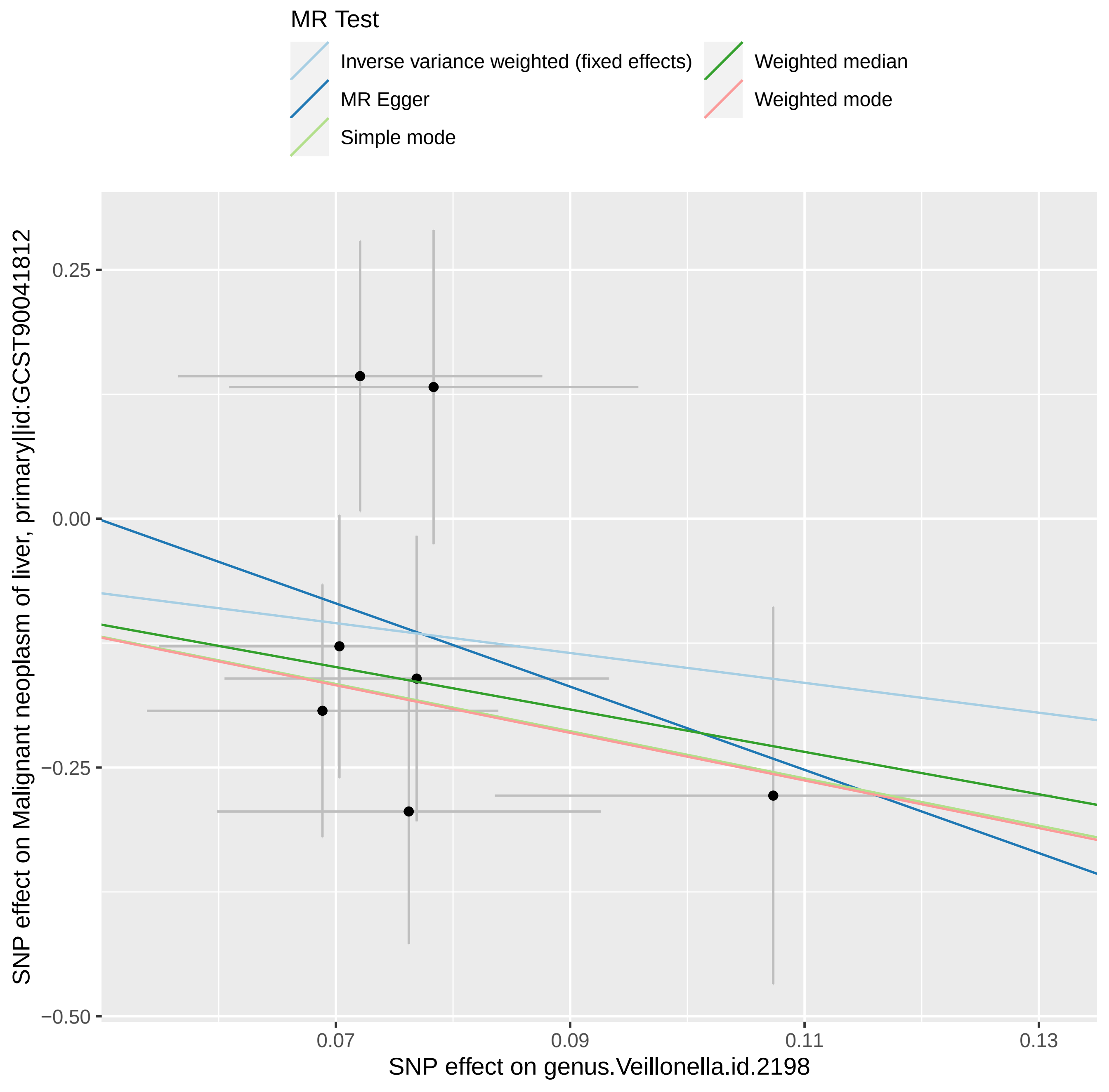

MR Test
Inverse variance weighted (fixed effects)
MR Egger
Simple mode
Weighted median
Weighted mode
0.25
0.00
−0.25
−0.50
0.07
0.09
0.11
0.13
SNP effect on genus.Veillonella.id.2198
SNP effect on Malignant neoplasm of liver, primary||id:GCST90041812
