# Supplementary figures and images for: Causal impact of gut microbiota on five liver diseases: insights from mendelian randomization and single-cell RNA sequencing
Source: Front Genet. 2024 Nov 11;15:1362139. doi: 10.3389/fgene.2024.1362139 (PMC11586359; doi:10.3389/fgene.2024.1362139)

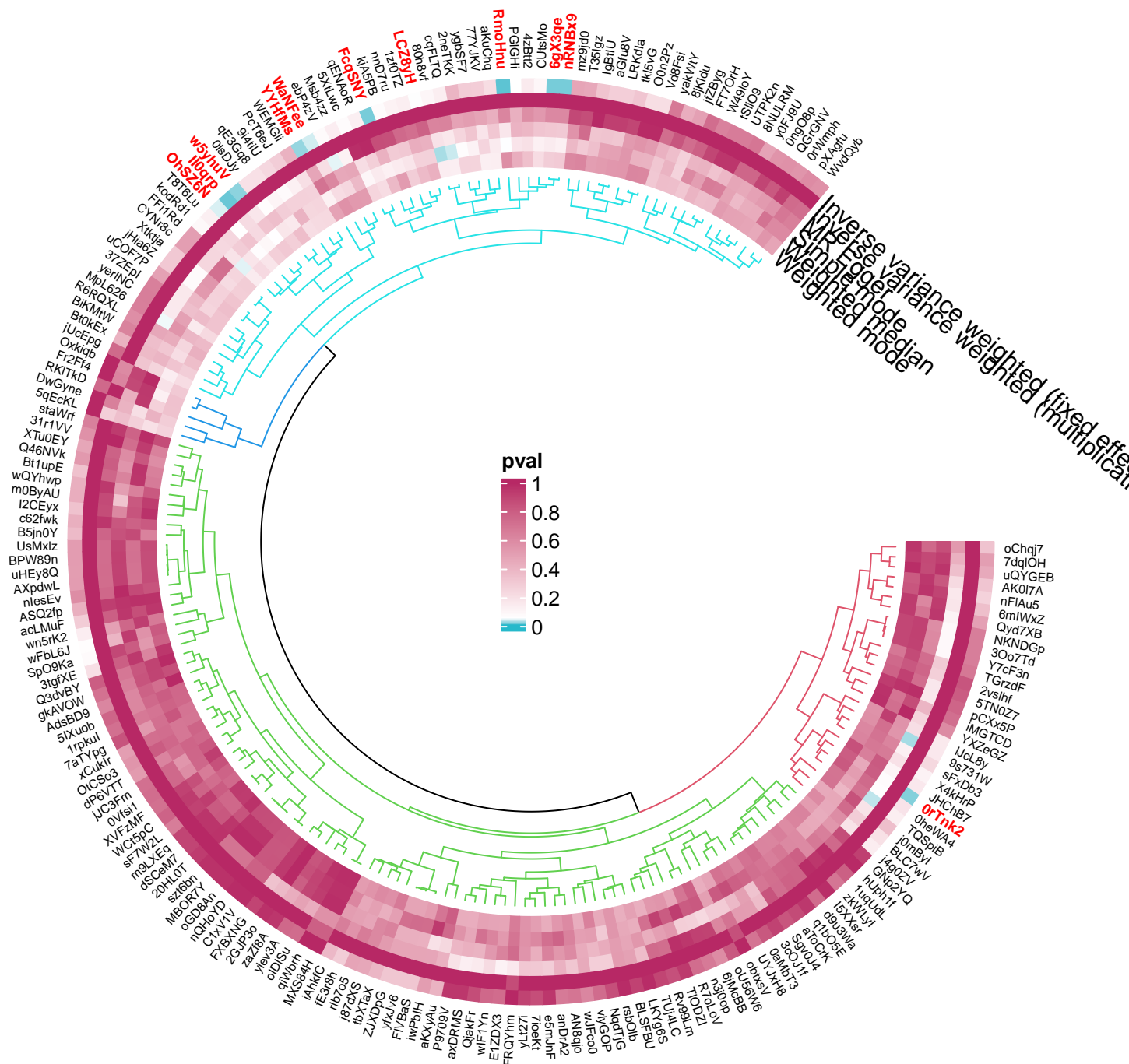

Supplement: Supplementary file 1 [file DataSheet1.zip › Annex 1 _Data/MR results/Alcoholic liver disease/Alcoholic liver disease -figures/Alcoholic liver disease -circos.pdf]

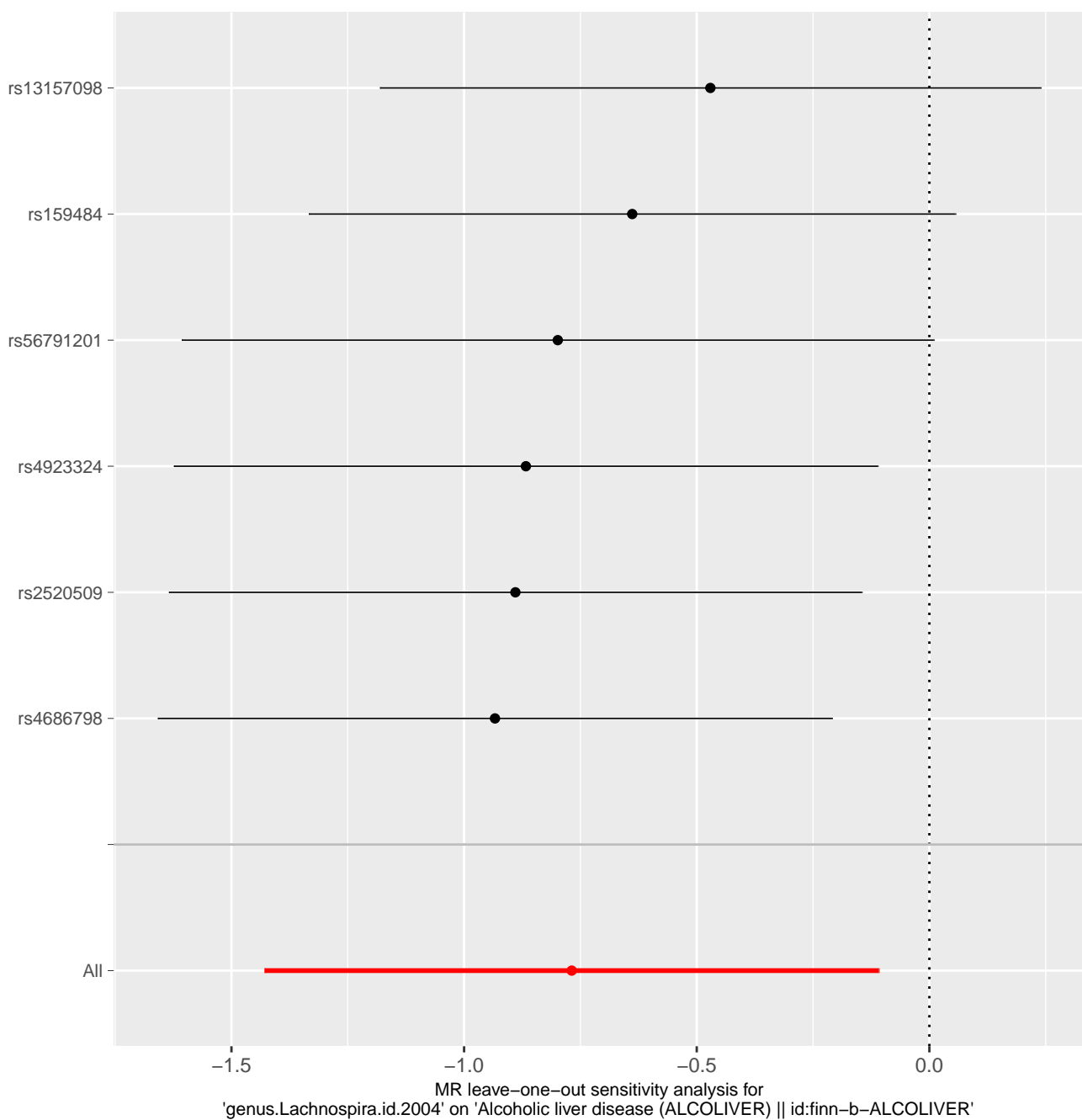

Supplement: Supplementary file 1 [file DataSheet1.zip › Annex 1 _Data/MR results/Alcoholic liver disease/Alcoholic liver disease -figures/LeaveOne_finn-b-ALCOLIVER_class.Actinobacteria.id.419.pdf]

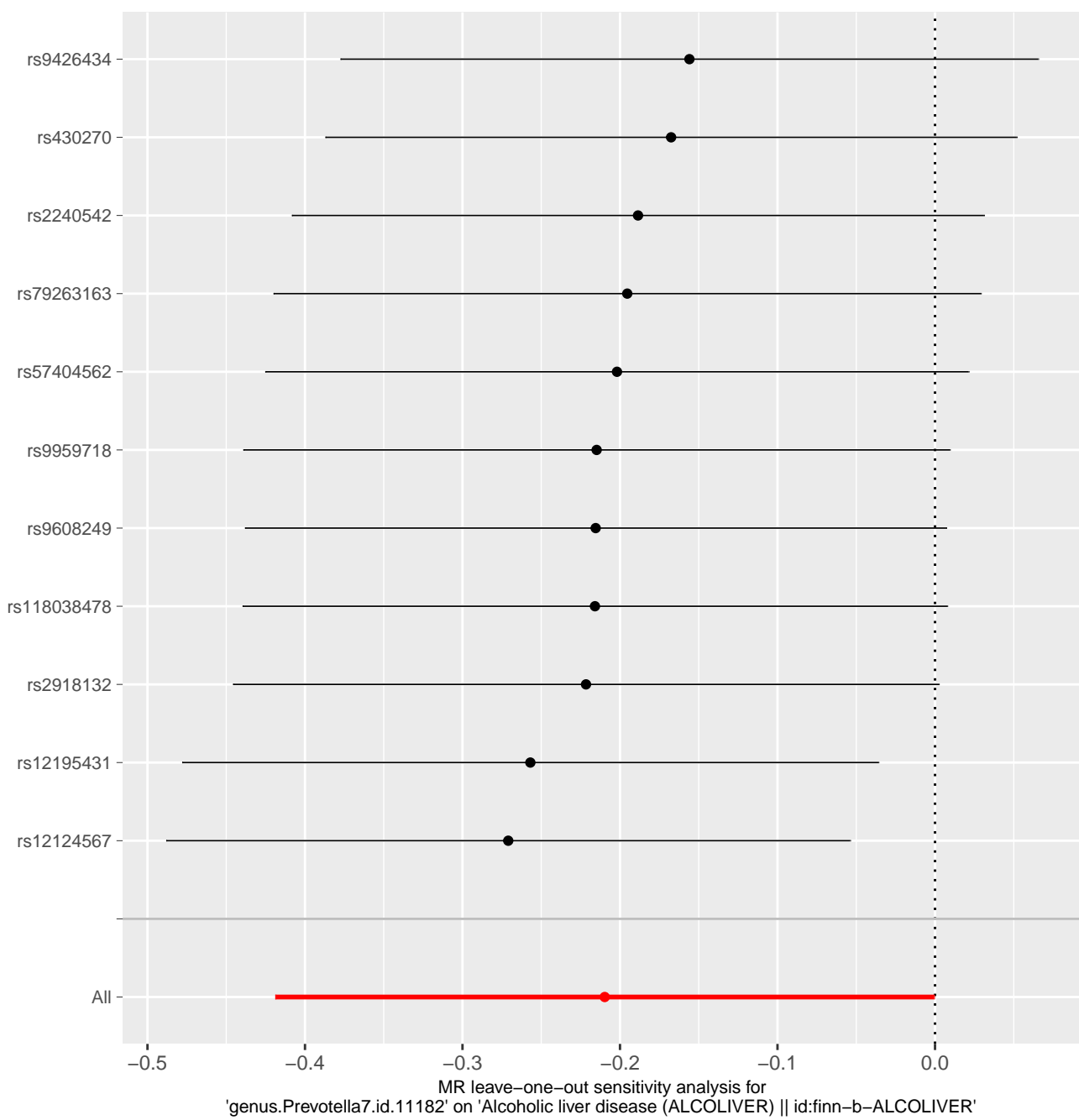

Supplement: Supplementary file 1 [file DataSheet1.zip › Annex 1 _Data/MR results/Alcoholic liver disease/Alcoholic liver disease -figures/LeaveOne_finn-b-ALCOLIVER_class.Betaproteobacteria.id.2867.pdf]

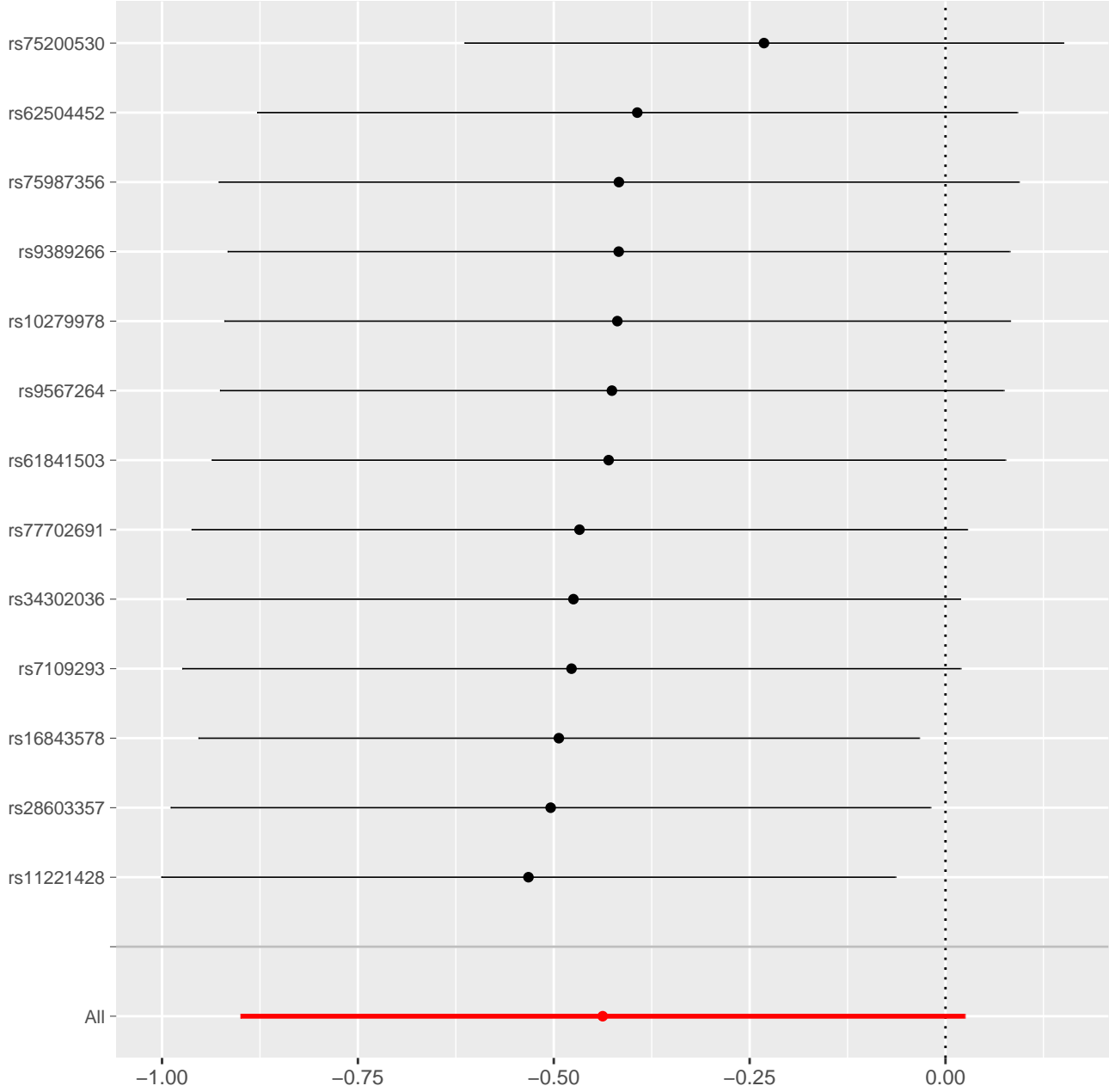

Supplement: Supplementary file 1 [file DataSheet1.zip › Annex 1 _Data/MR results/Alcoholic liver disease/Alcoholic liver disease -figures/LeaveOne_finn-b-ALCOLIVER_class.Erysipelotrichia.id.2147.pdf]

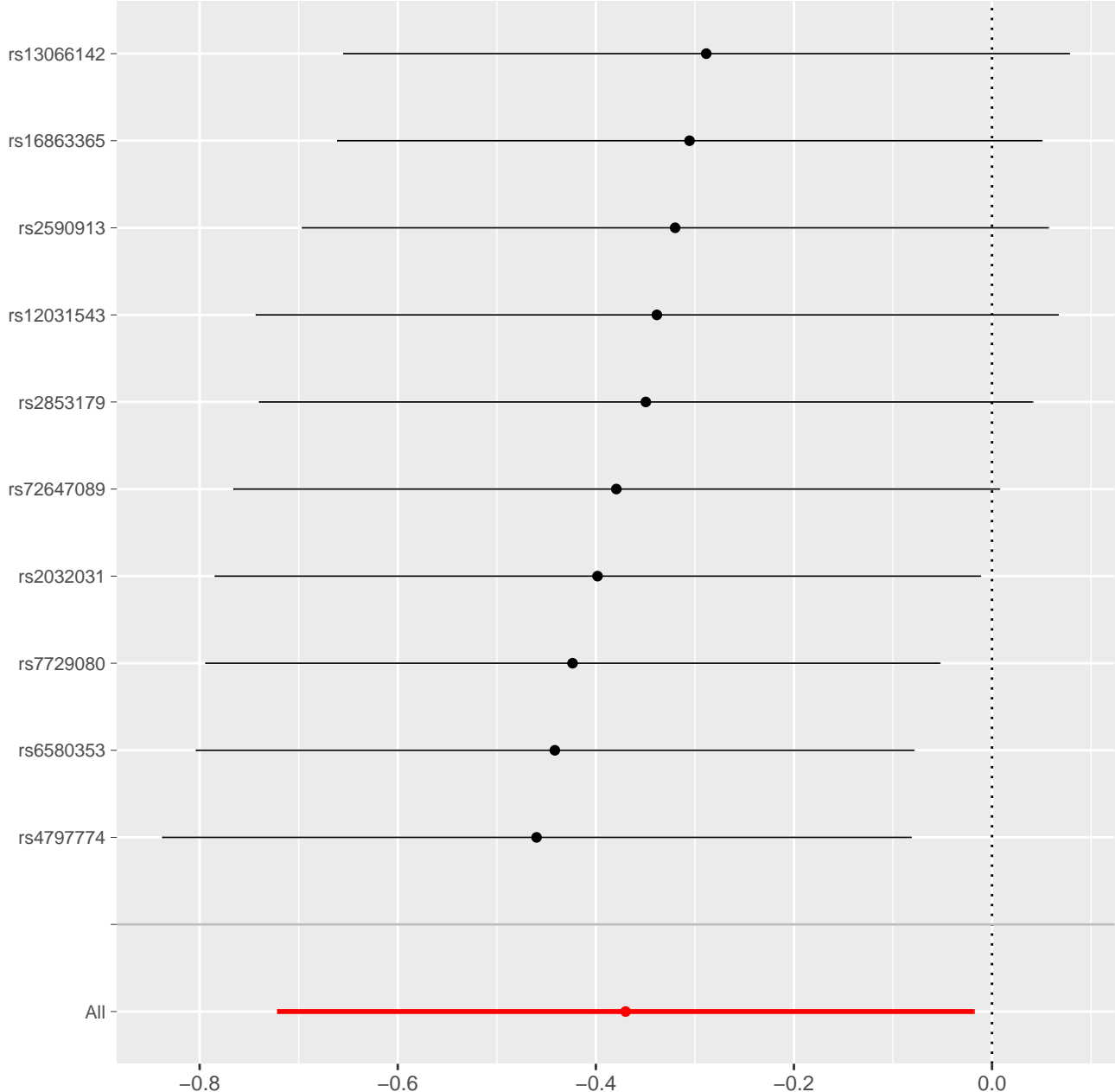

Supplement: Supplementary file 1 [file DataSheet1.zip › Annex 1 _Data/MR results/Alcoholic liver disease/Alcoholic liver disease -figures/LeaveOne_finn-b-ALCOLIVER_class.Gammaproteobacteria.id.3303.pdf]

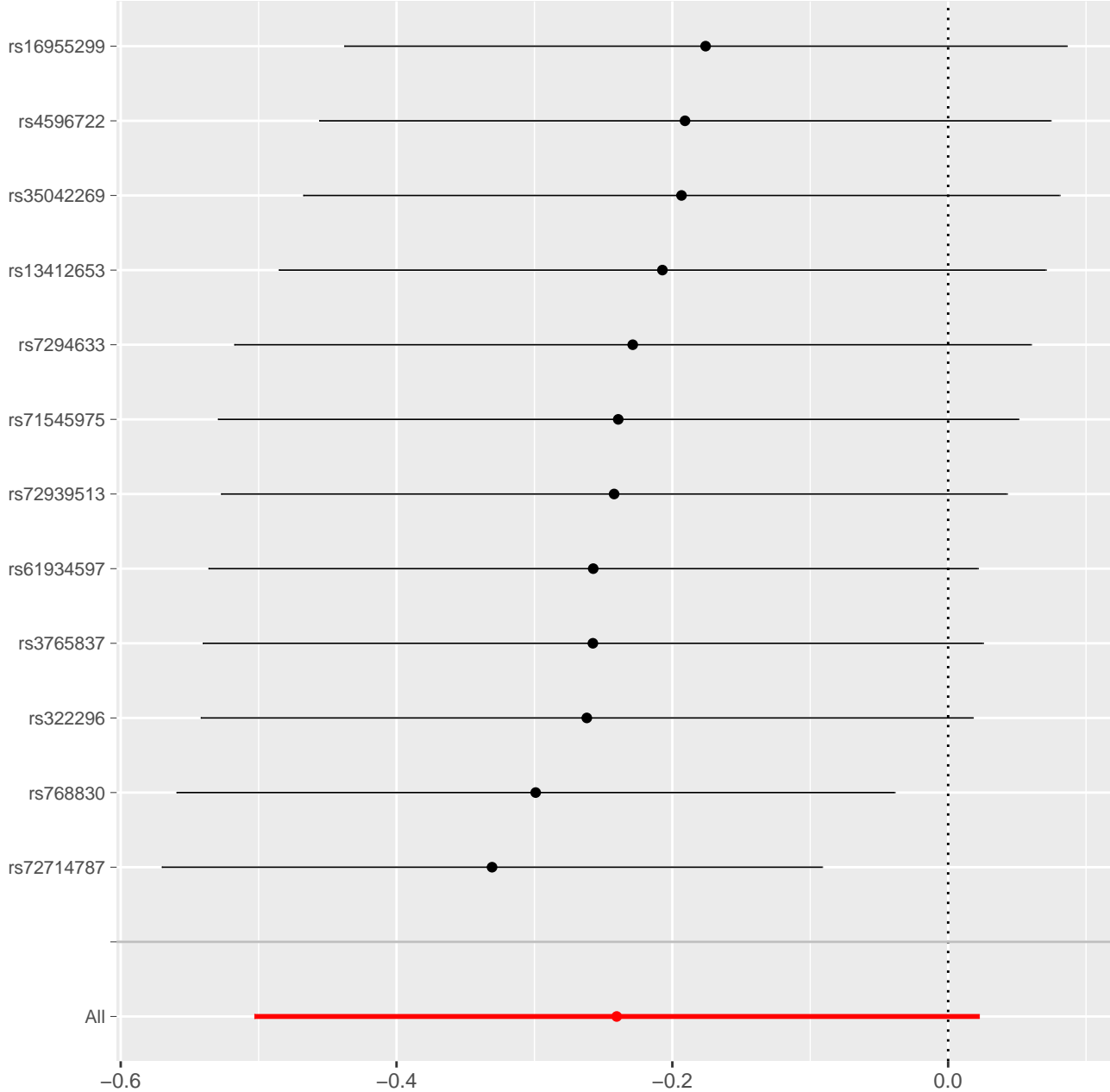

Supplement: Supplementary file 1 [file DataSheet1.zip › Annex 1 _Data/MR results/Alcoholic liver disease/Alcoholic liver disease -figures/LeaveOne_finn-b-ALCOLIVER_class.Lentisphaeria.id.2250.pdf]

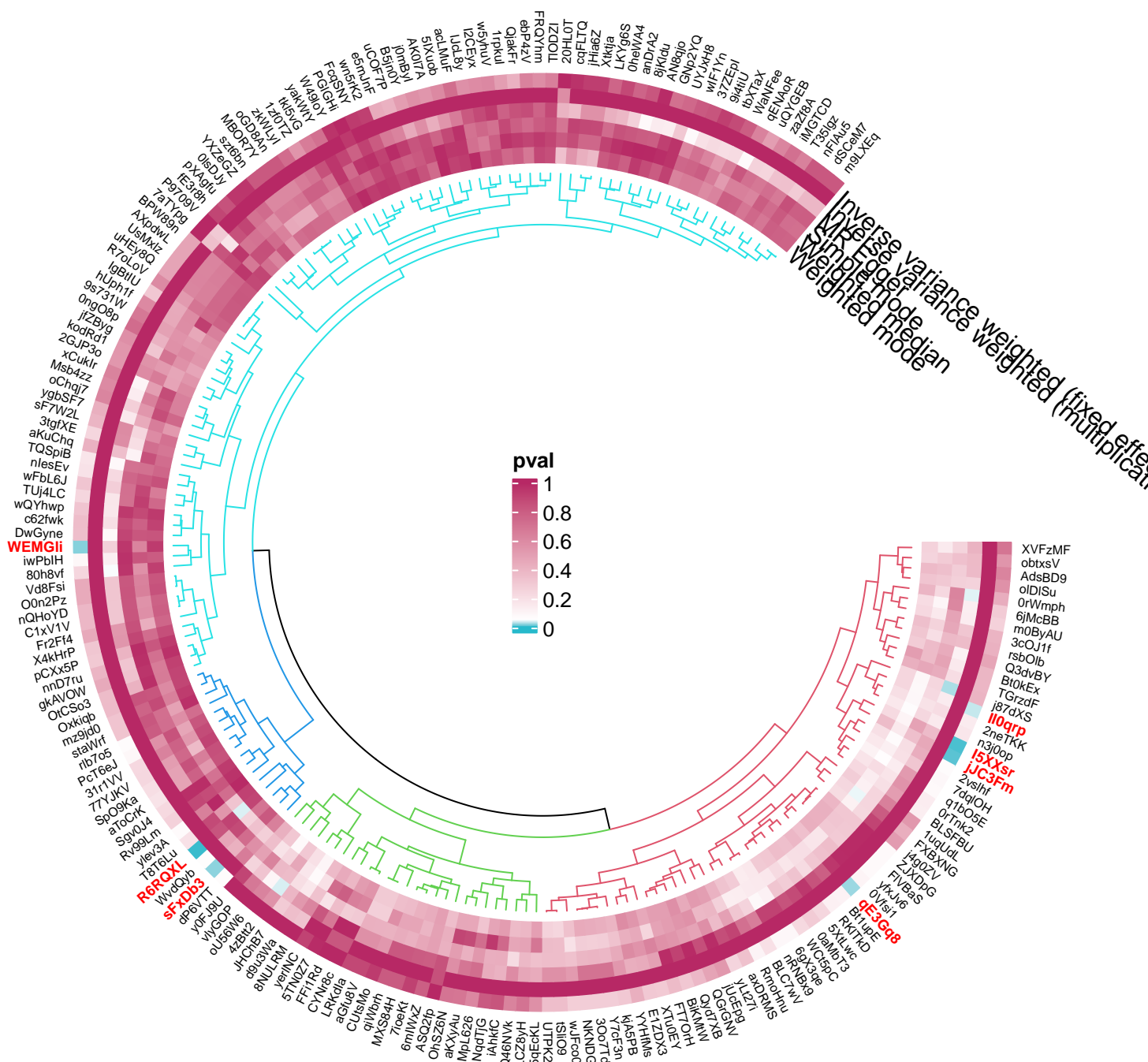

Supplement: Supplementary file 1 [file DataSheet1.zip › Annex 1 _Data/MR results/Benign neoplasm/Benign neoplasm-figures/Benign neoplasm-circos.pdf]

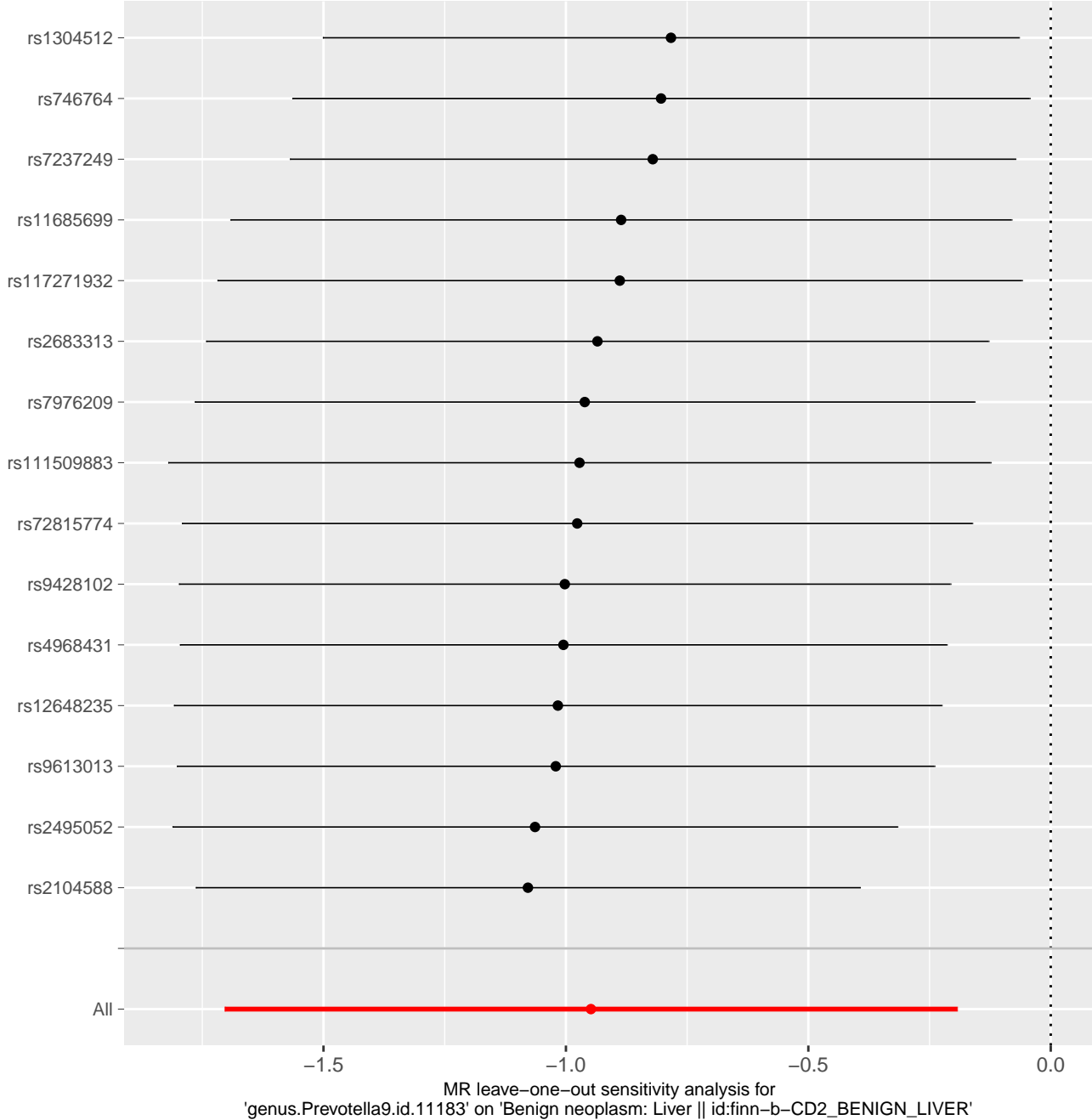

Supplement: Supplementary file 1 [file DataSheet1.zip › Annex 1 _Data/MR results/Benign neoplasm/Benign neoplasm-figures/LeaveOne_finn-b-CD2_BENIGN_LIVER_class.Actinobacteria.id.419.pdf]

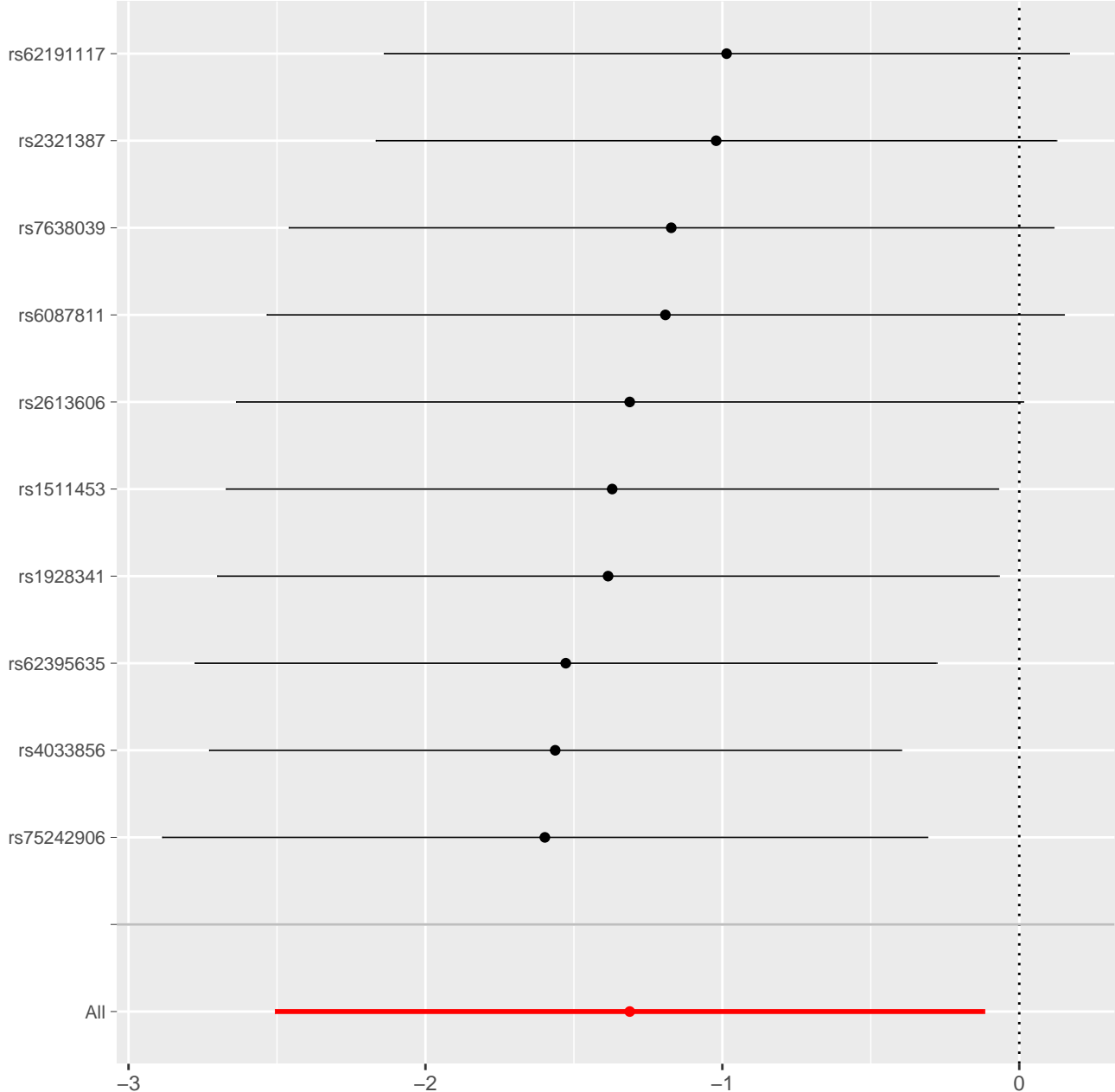

Supplement: Supplementary file 1 [file DataSheet1.zip › Annex 1 _Data/MR results/Benign neoplasm/Benign neoplasm-figures/LeaveOne_finn-b-CD2_BENIGN_LIVER_class.Clostridia.id.1859.pdf]

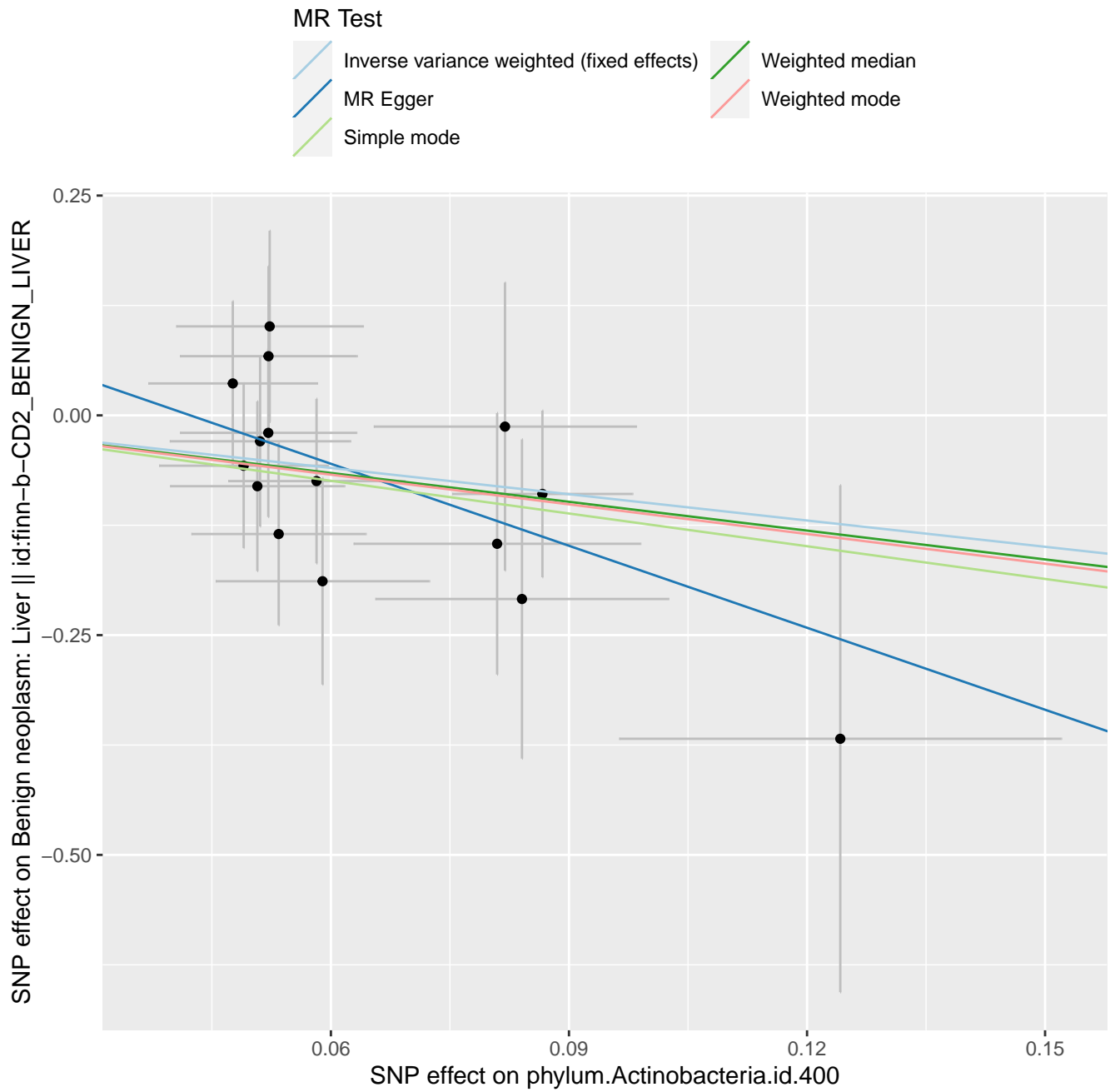

Supplement: Supplementary file 1 [file DataSheet1.zip › Annex 1 _Data/MR results/Benign neoplasm/Benign neoplasm-figures/ScatterPlot_finn-b-CD2_BENIGN_LIVER_class.Alphaproteobacteria.id.2379.pdf]

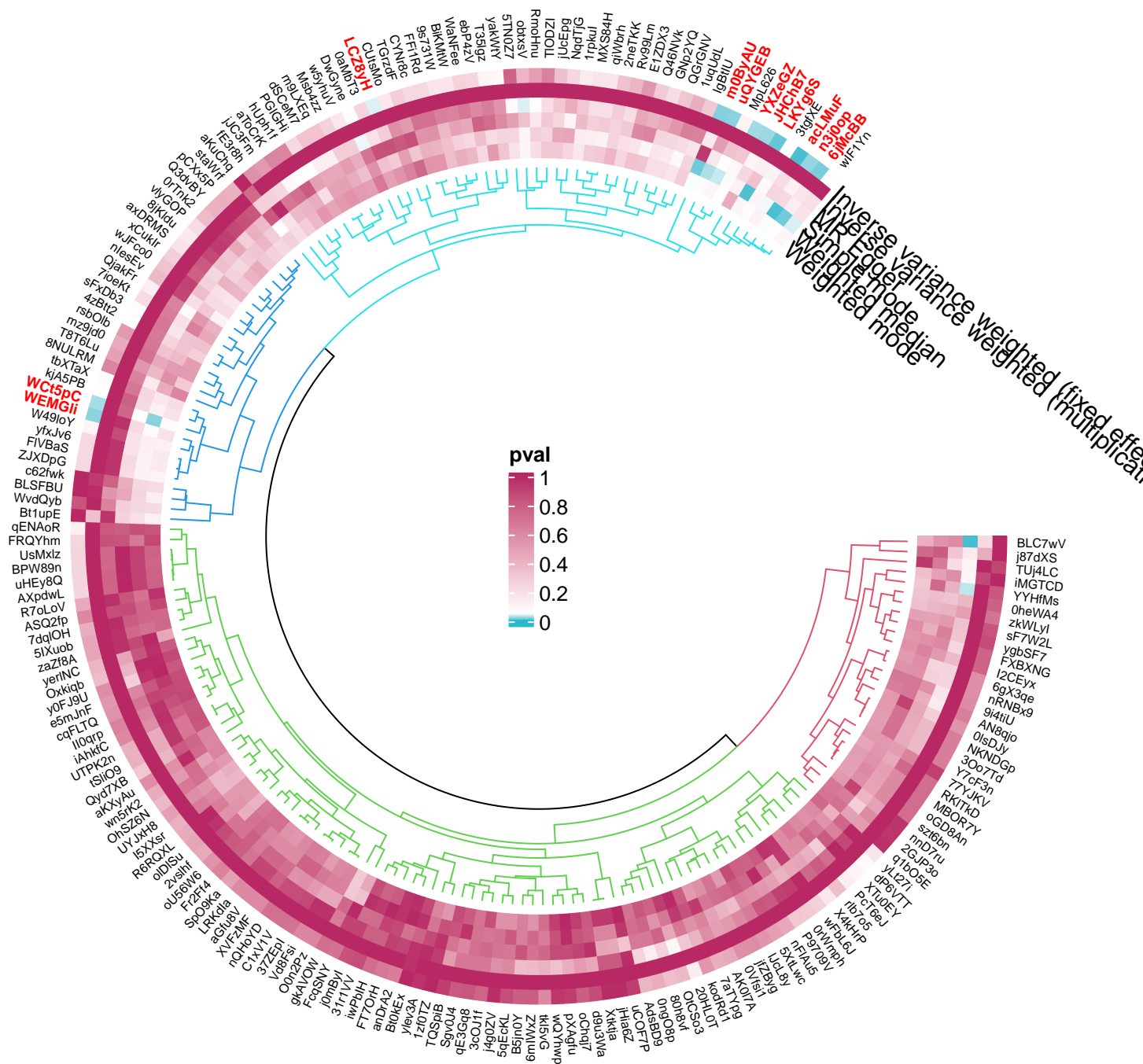

Supplement: Supplementary file 1 [file DataSheet1.zip › Annex 1 _Data/MR results/Cirrhosis/Cirrhosis-figure/Cirrhosis-circos.pdf]

# MR Forest Plot

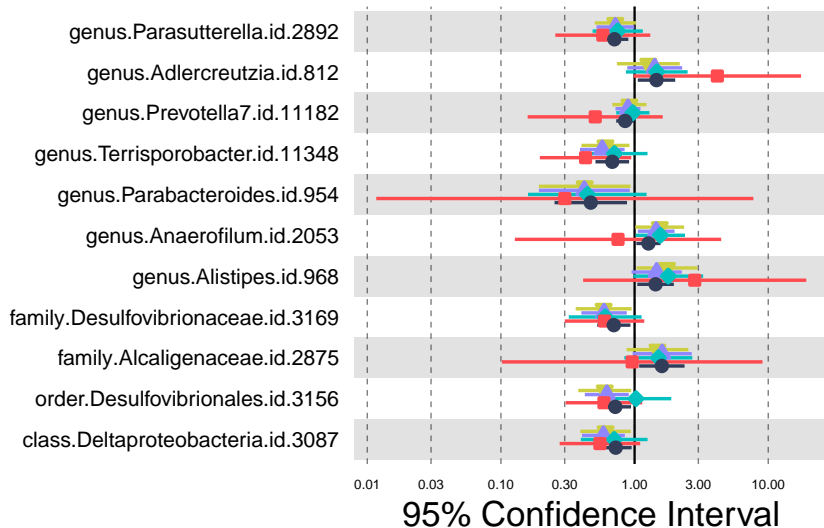

## method

- Weighted mode
- Weighted median
- Simple mode
- MR Egger
- IVW(fe)

Supplement: Supplementary file 1 [file DataSheet1.zip › Annex 1 _Data/MR results/Cirrhosis/Cirrhosis-figure/Cirrhosis-Forest plot.pdf]

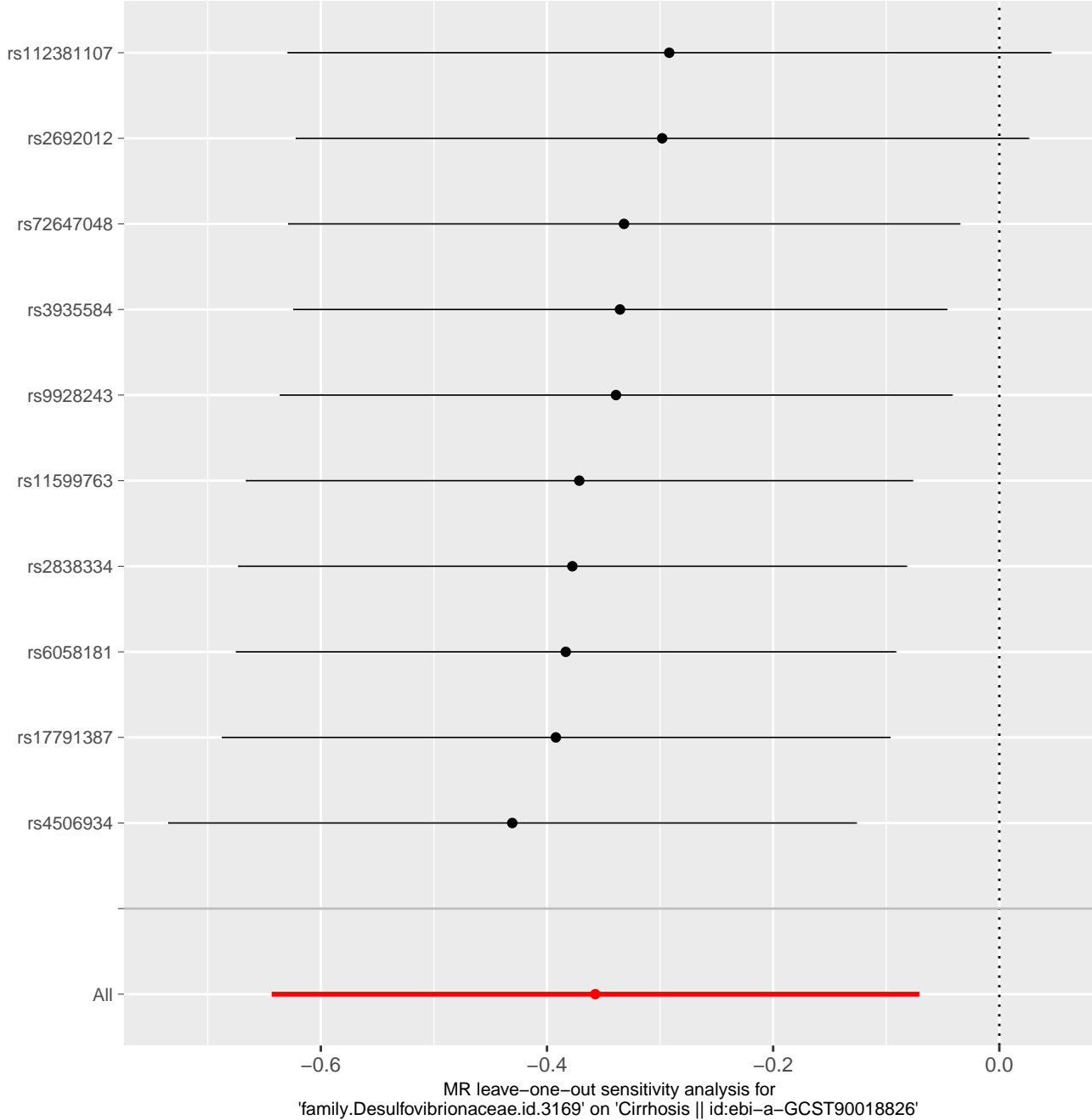

Supplement: Supplementary file 1 [file DataSheet1.zip › Annex 1 _Data/MR results/Cirrhosis/Cirrhosis-figure/LeaveOne_ebi-a-GCST90018826_class.Actinobacteria.id.419.pdf]

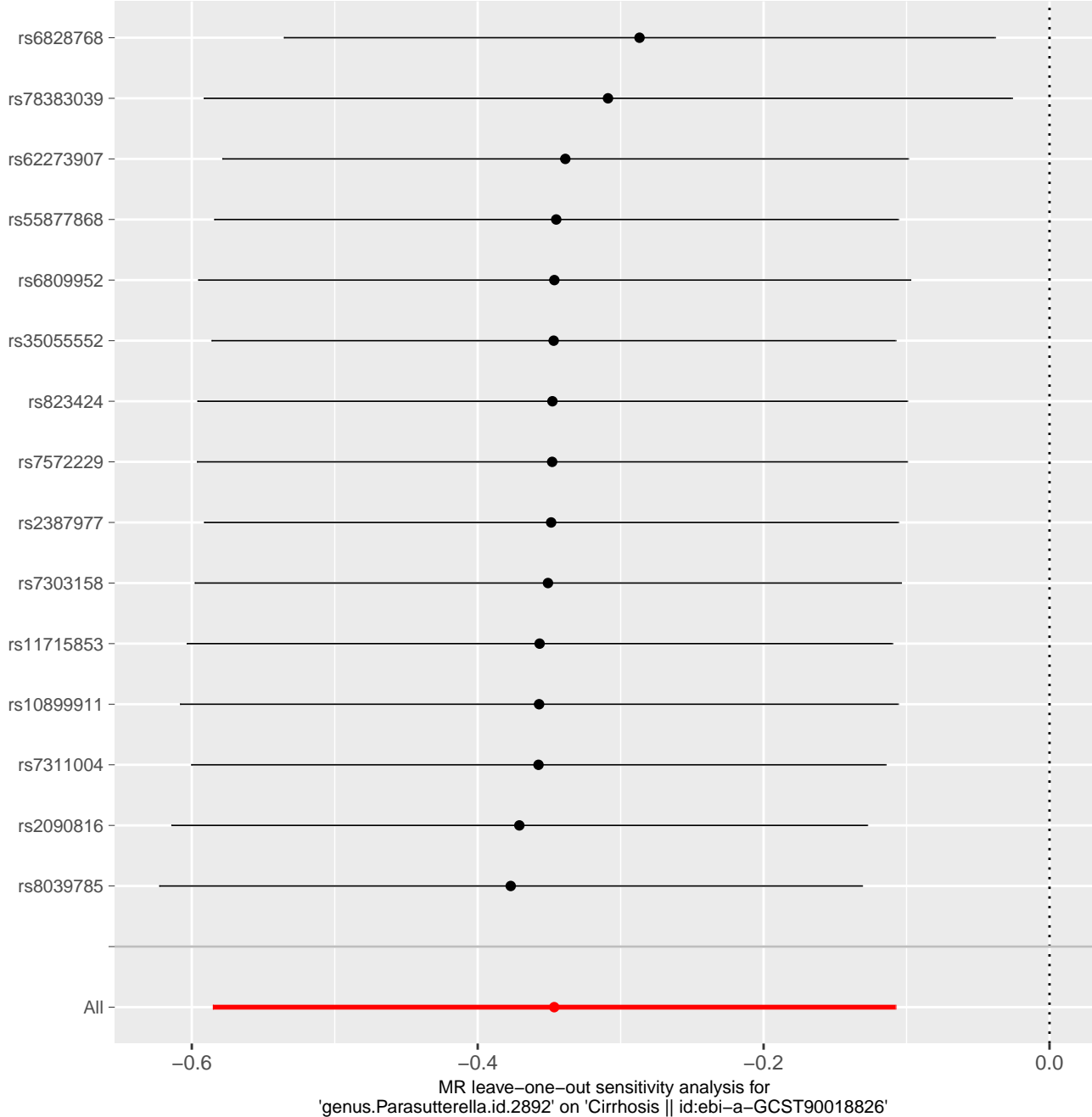

Supplement: Supplementary file 1 [file DataSheet1.zip › Annex 1 _Data/MR results/Cirrhosis/Cirrhosis-figure/LeaveOne_ebi-a-GCST90018826_class.Alphaproteobacteria.id.2379.pdf]

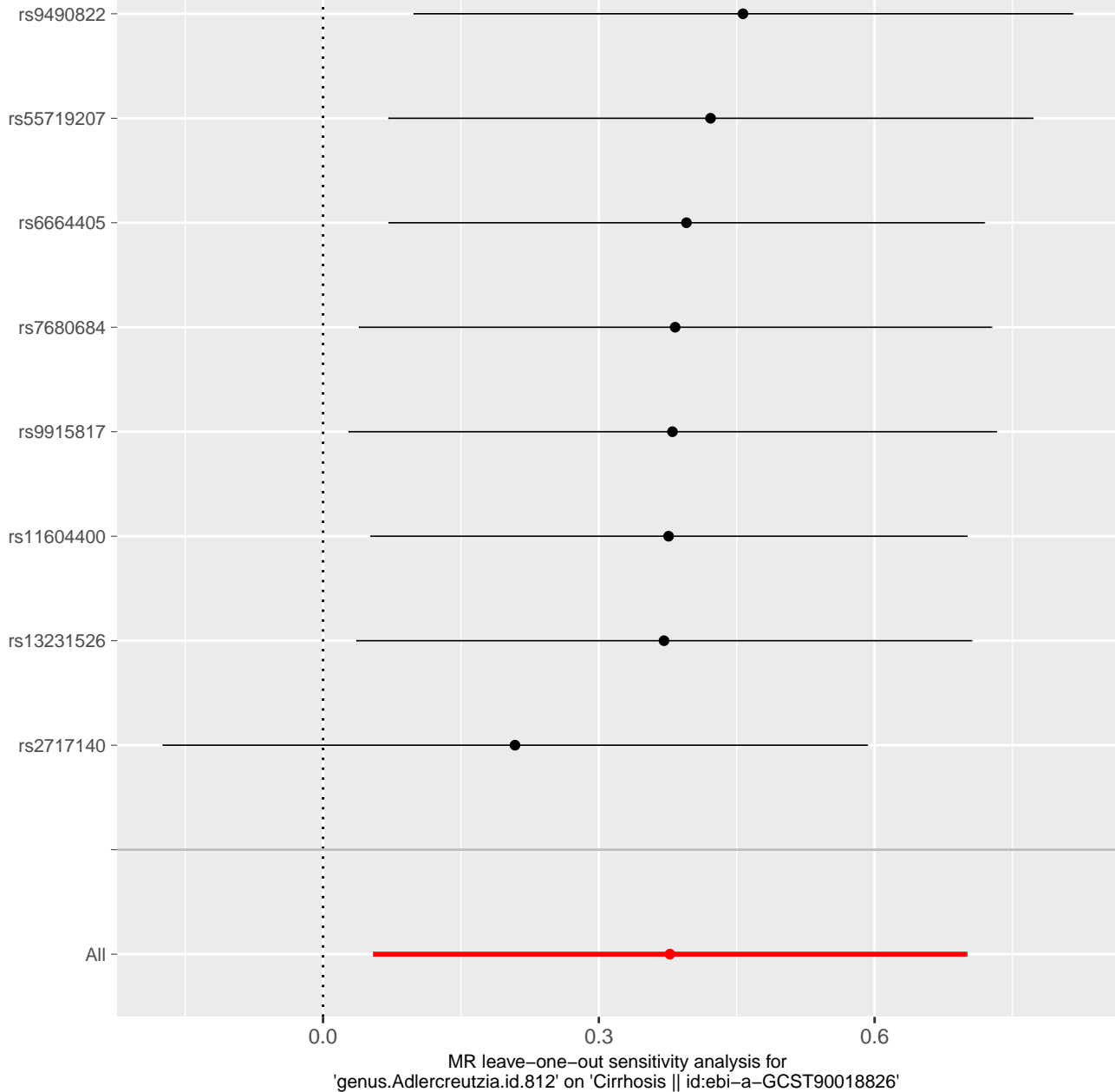

Supplement: Supplementary file 1 [file DataSheet1.zip › Annex 1 _Data/MR results/Cirrhosis/Cirrhosis-figure/LeaveOne_ebi-a-GCST90018826_class.Bacilli.id.1673.pdf]

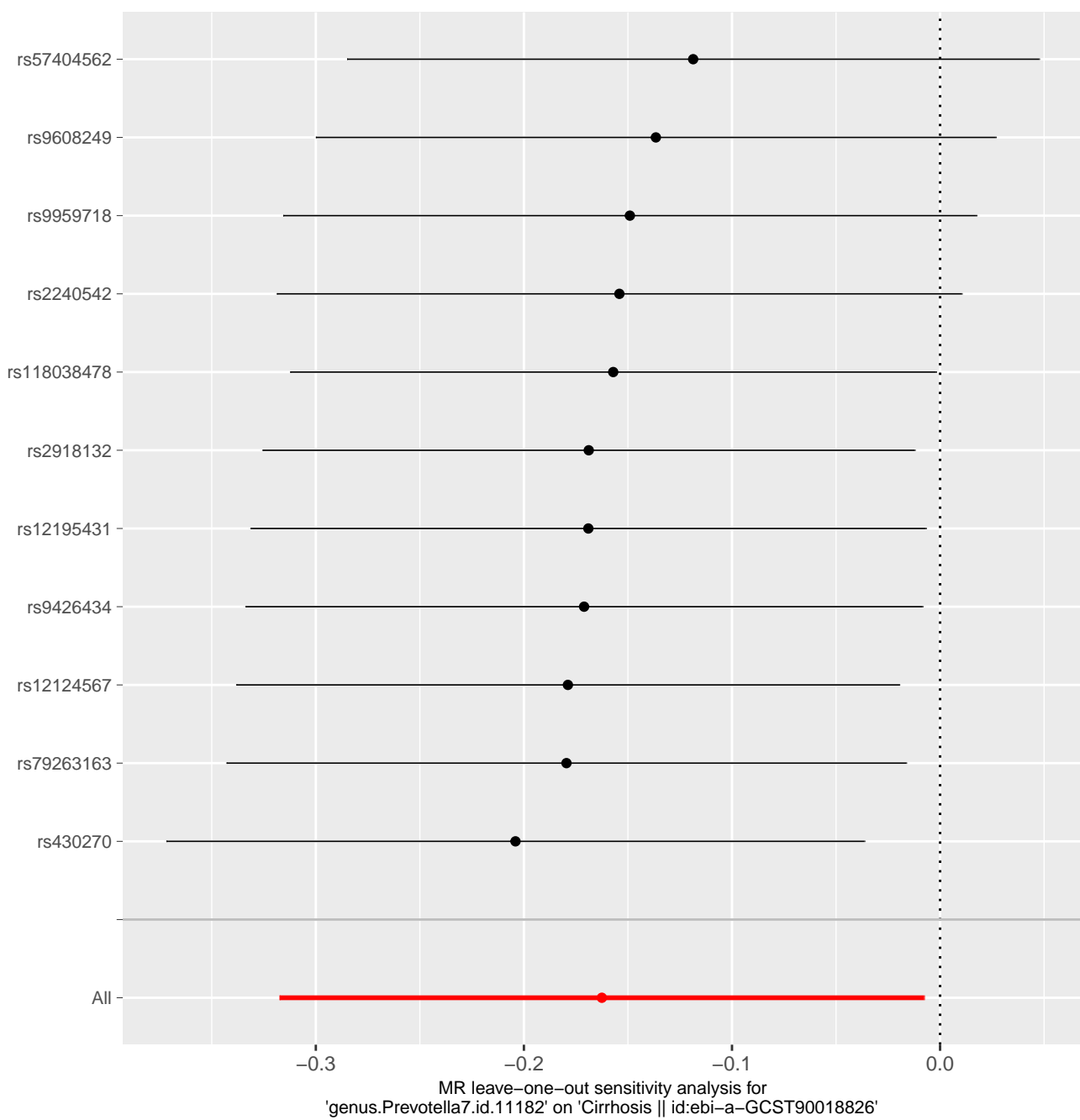

Supplement: Supplementary file 1 [file DataSheet1.zip › Annex 1 _Data/MR results/Cirrhosis/Cirrhosis-figure/LeaveOne_ebi-a-GCST90018826_class.Bacteroidia.id.912.pdf]

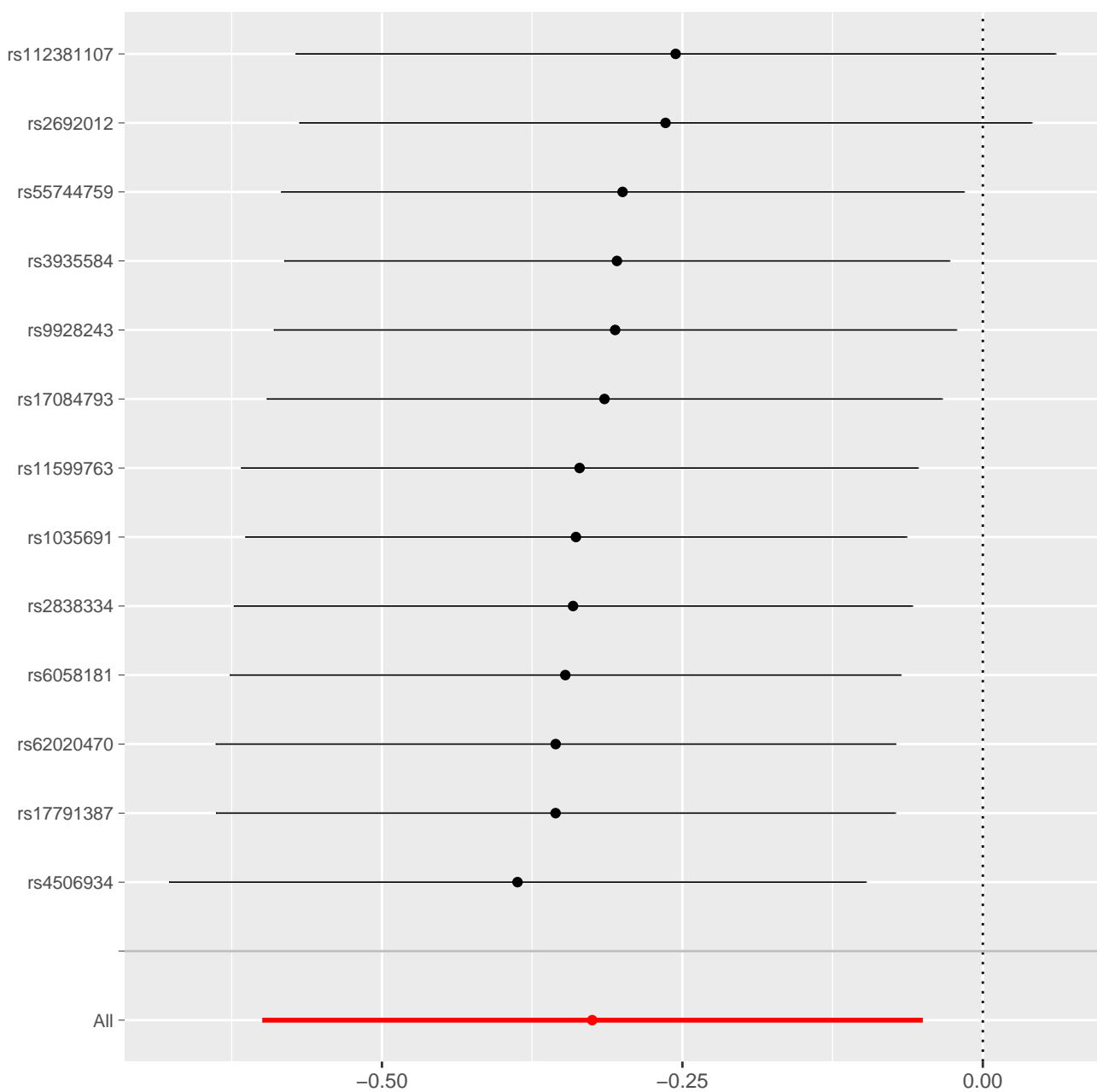

Supplement: Supplementary file 1 [file DataSheet1.zip › Annex 1 _Data/MR results/Cirrhosis/Cirrhosis-figure/LeaveOne_ebi-a-GCST90018826_class.Coriobacteriia.id.809.pdf]

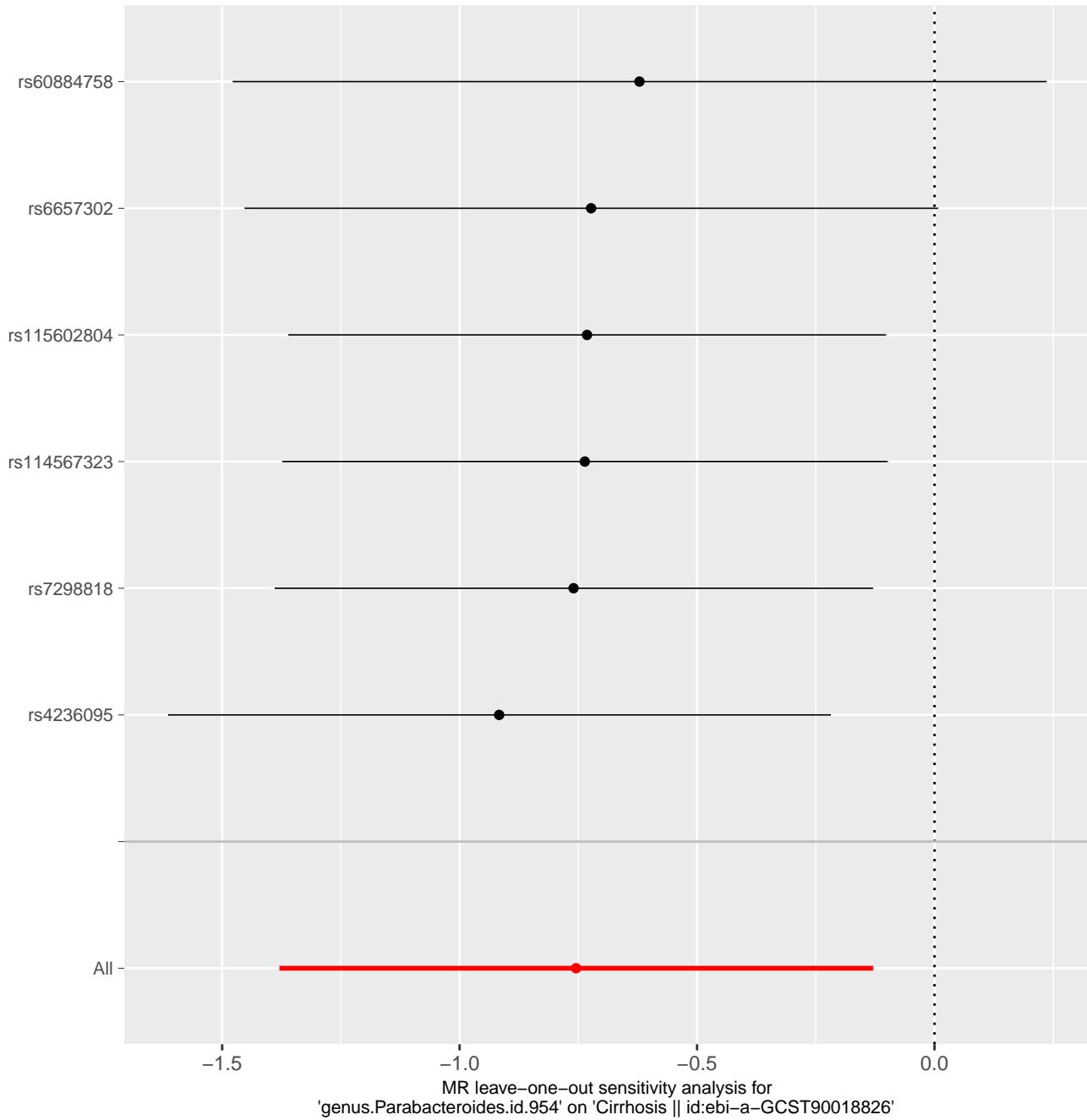

Supplement: Supplementary file 1 [file DataSheet1.zip › Annex 1 _Data/MR results/Cirrhosis/Cirrhosis-figure/LeaveOne_ebi-a-GCST90018826_class.Deltaproteobacteria.id.3087.pdf]

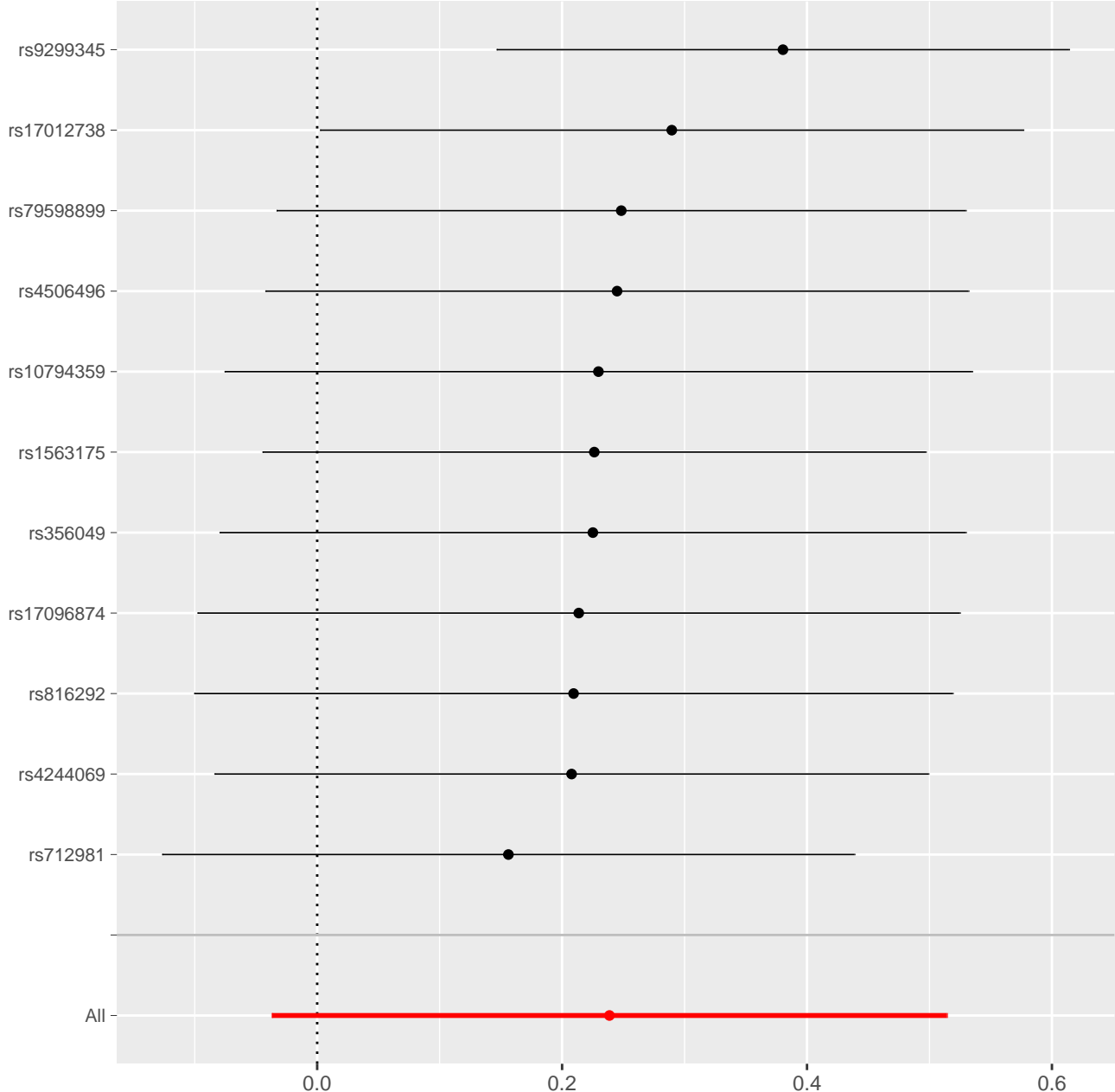

MR leave-one-out sensitivity analysis for  
'genus.Anaerofilum.id.2053' on 'Cirrhosis || id:ebi-a-GCST90018826'

Supplement: Supplementary file 1 [file DataSheet1.zip › Annex 1 _Data/MR results/Cirrhosis/Cirrhosis-figure/LeaveOne_ebi-a-GCST90018826_class.Erysipelotrichia.id.2147.pdf]

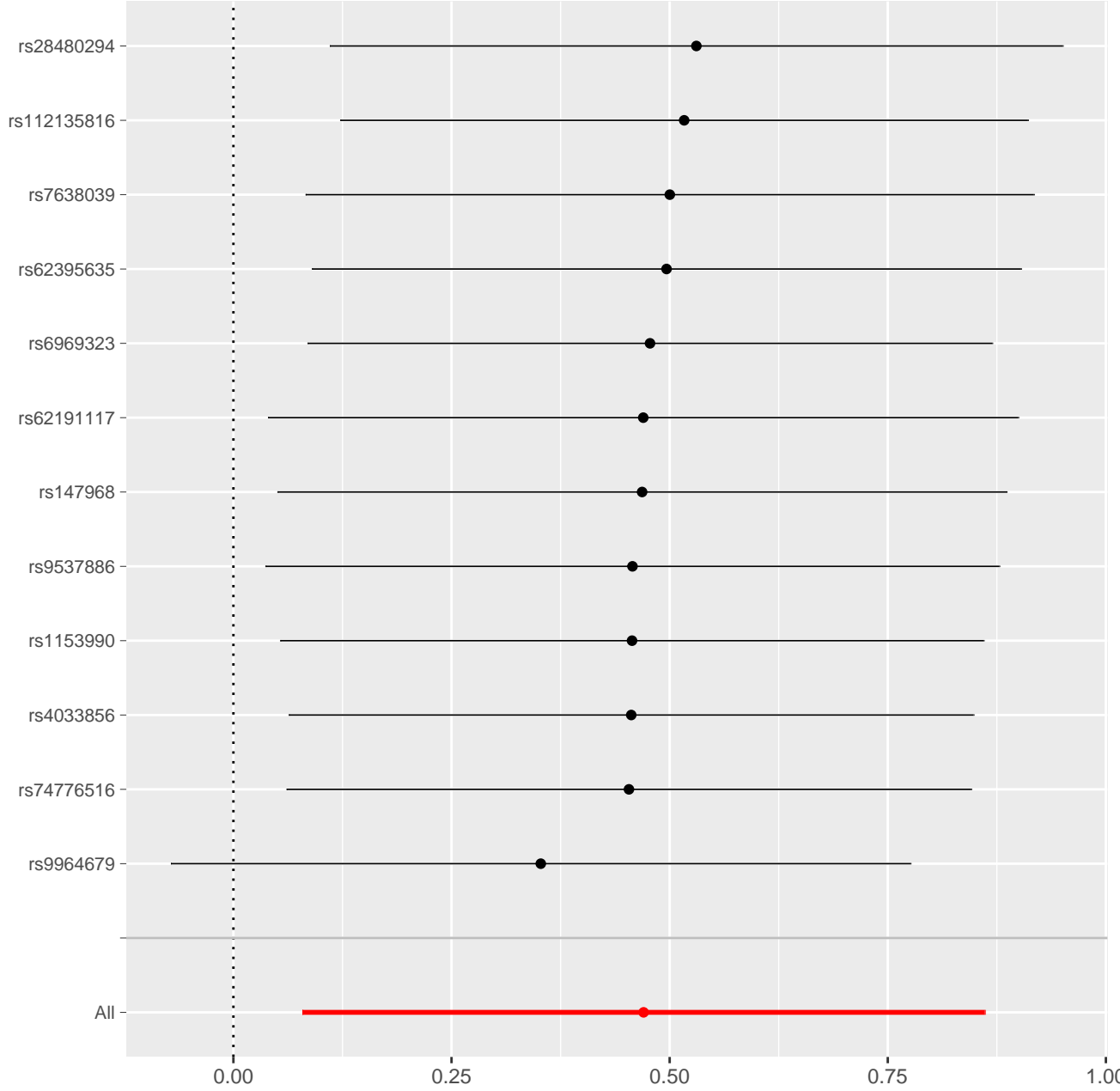

MR leave-one-out sensitivity analysis for  
'family.Alcaligenaceae.id.2875' on 'Cirrhosis || id:ebi-a-GCST90018826'

Supplement: Supplementary file 1 [file DataSheet1.zip › Annex 1 _Data/MR results/Cirrhosis/Cirrhosis-figure/LeaveOne_ebi-a-GCST90018826_class.Gammaproteobacteria.id.3303.pdf]

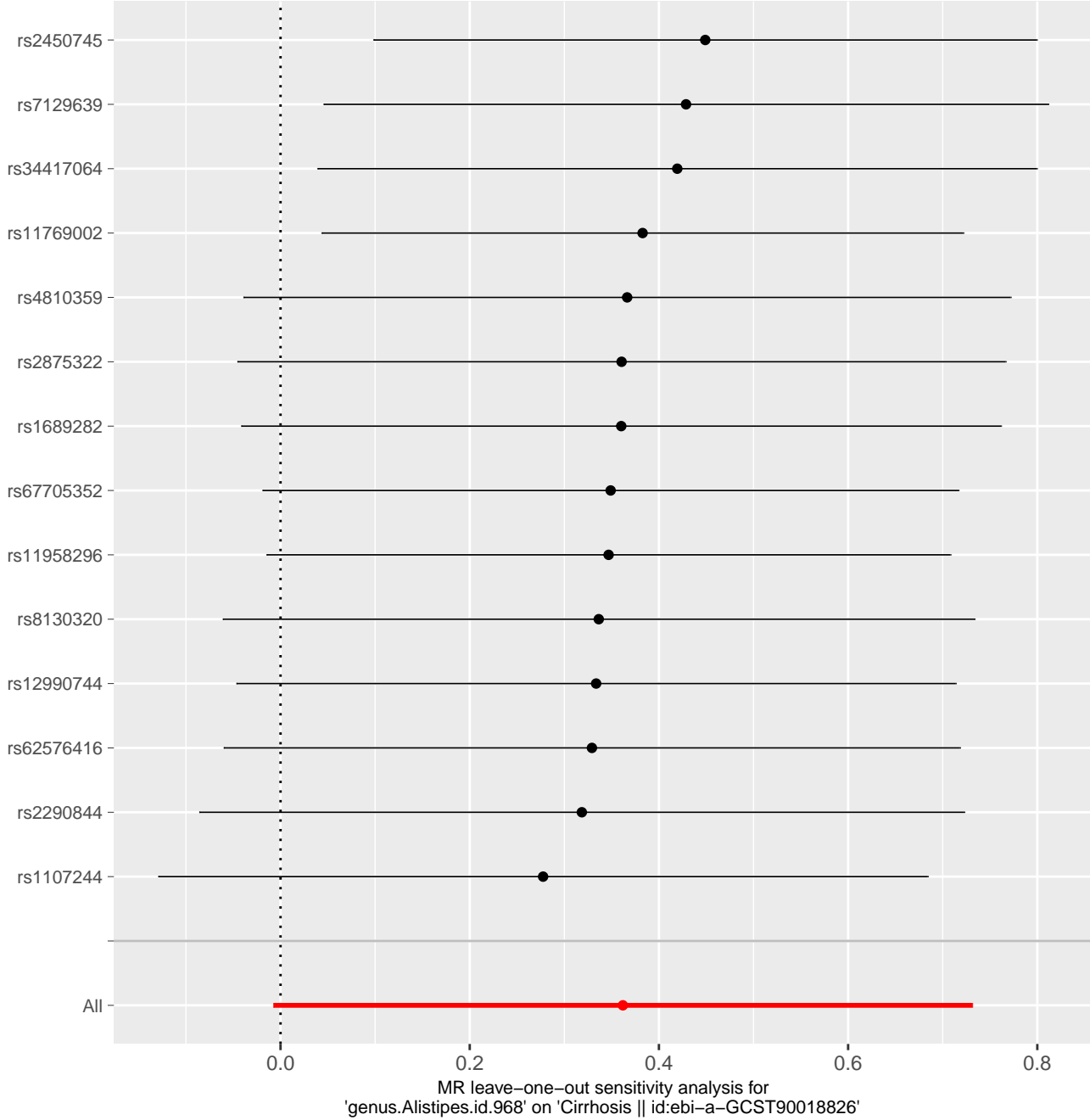

Supplement: Supplementary file 1 [file DataSheet1.zip › Annex 1 _Data/MR results/Cirrhosis/Cirrhosis-figure/LeaveOne_ebi-a-GCST90018826_class.Lentisphaeria.id.2250.pdf]

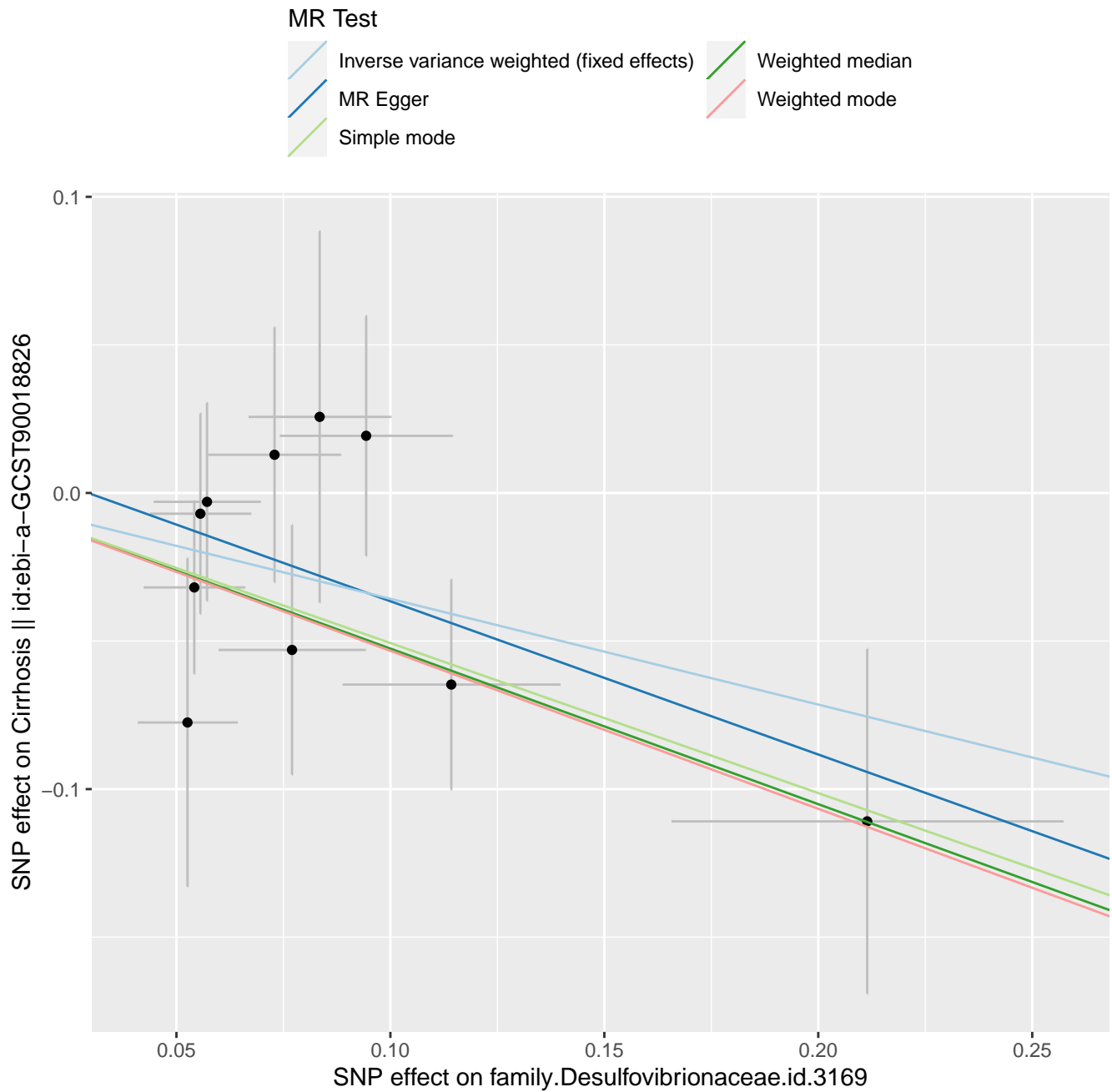

Supplement: Supplementary file 1 [file DataSheet1.zip › Annex 1 _Data/MR results/Cirrhosis/Cirrhosis-figure/ScatterPlot_ebi-a-GCST90018826_class.Actinobacteria.id.419.pdf]

- Inverse variance weighted (fixed effects)
- MR Egger
- Simple mode
- Weighted median
- Weighted mode

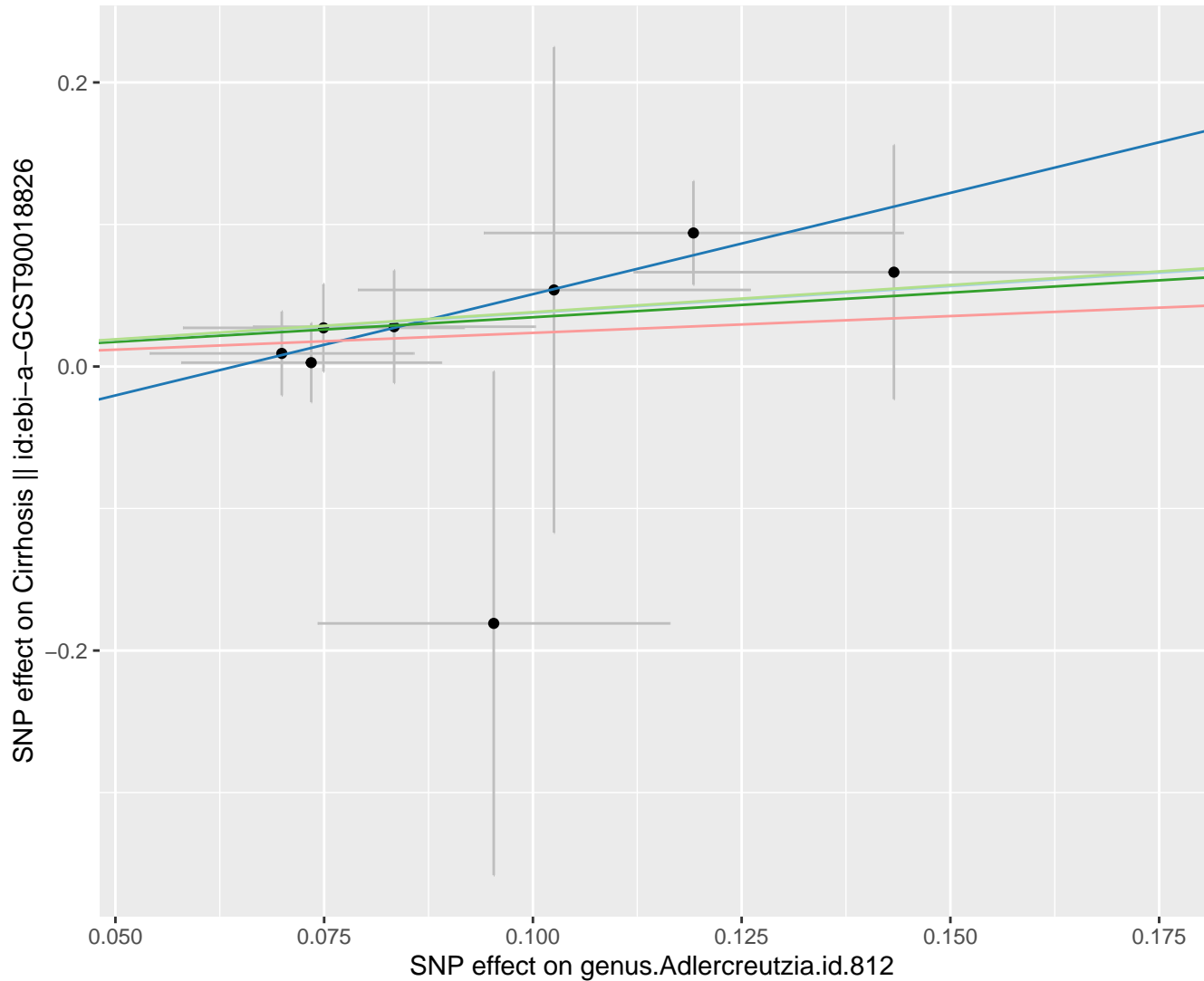

Supplement: Supplementary file 1 [file DataSheet1.zip › Annex 1 _Data/MR results/Cirrhosis/Cirrhosis-figure/ScatterPlot_ebi-a-GCST90018826_class.Bacilli.id.1673.pdf]

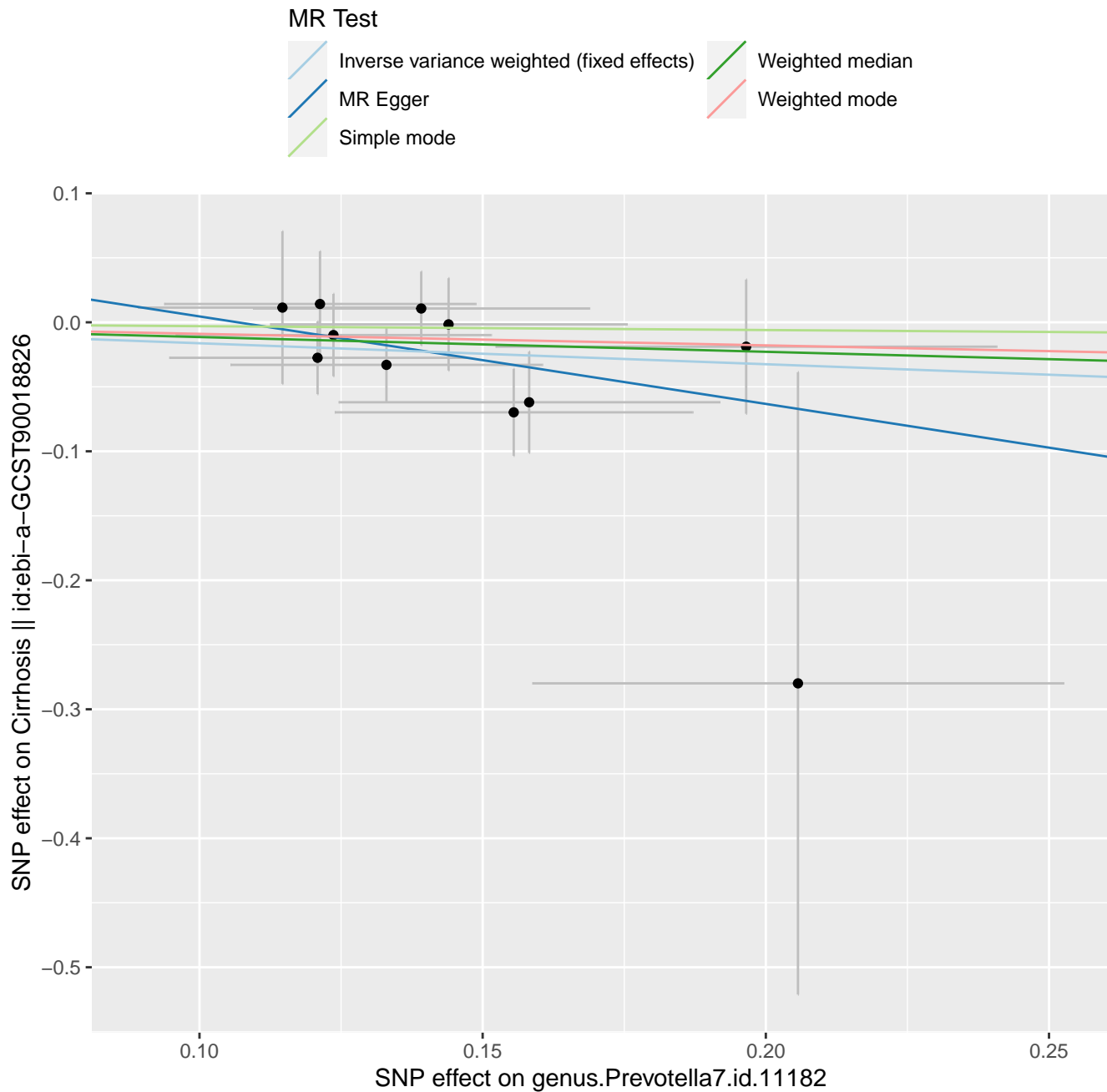

Supplement: Supplementary file 1 [file DataSheet1.zip › Annex 1 _Data/MR results/Cirrhosis/Cirrhosis-figure/ScatterPlot_ebi-a-GCST90018826_class.Bacteroidia.id.912.pdf]

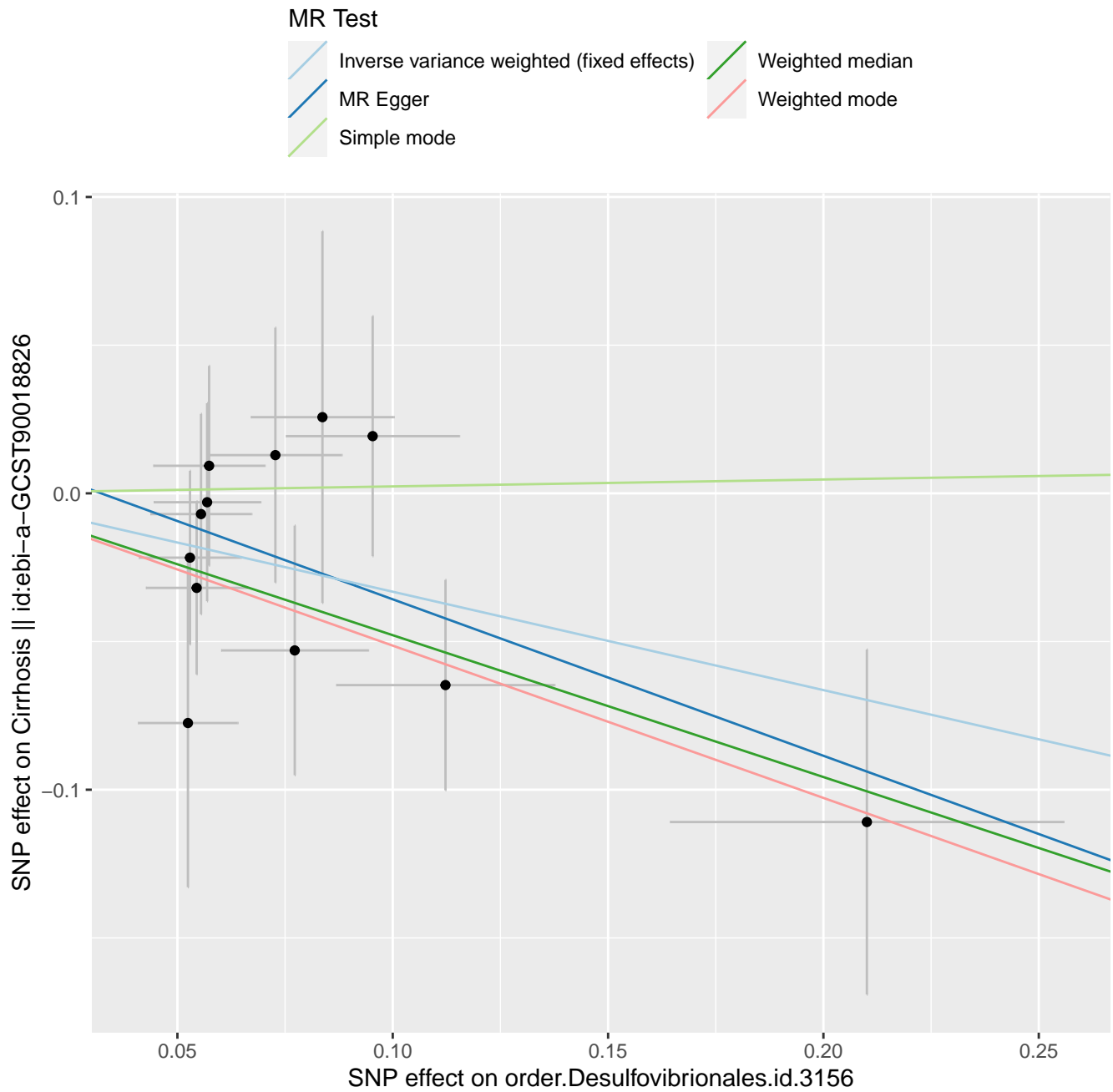

Supplement: Supplementary file 1 [file DataSheet1.zip › Annex 1 _Data/MR results/Cirrhosis/Cirrhosis-figure/ScatterPlot_ebi-a-GCST90018826_class.Clostridia.id.1859.pdf]

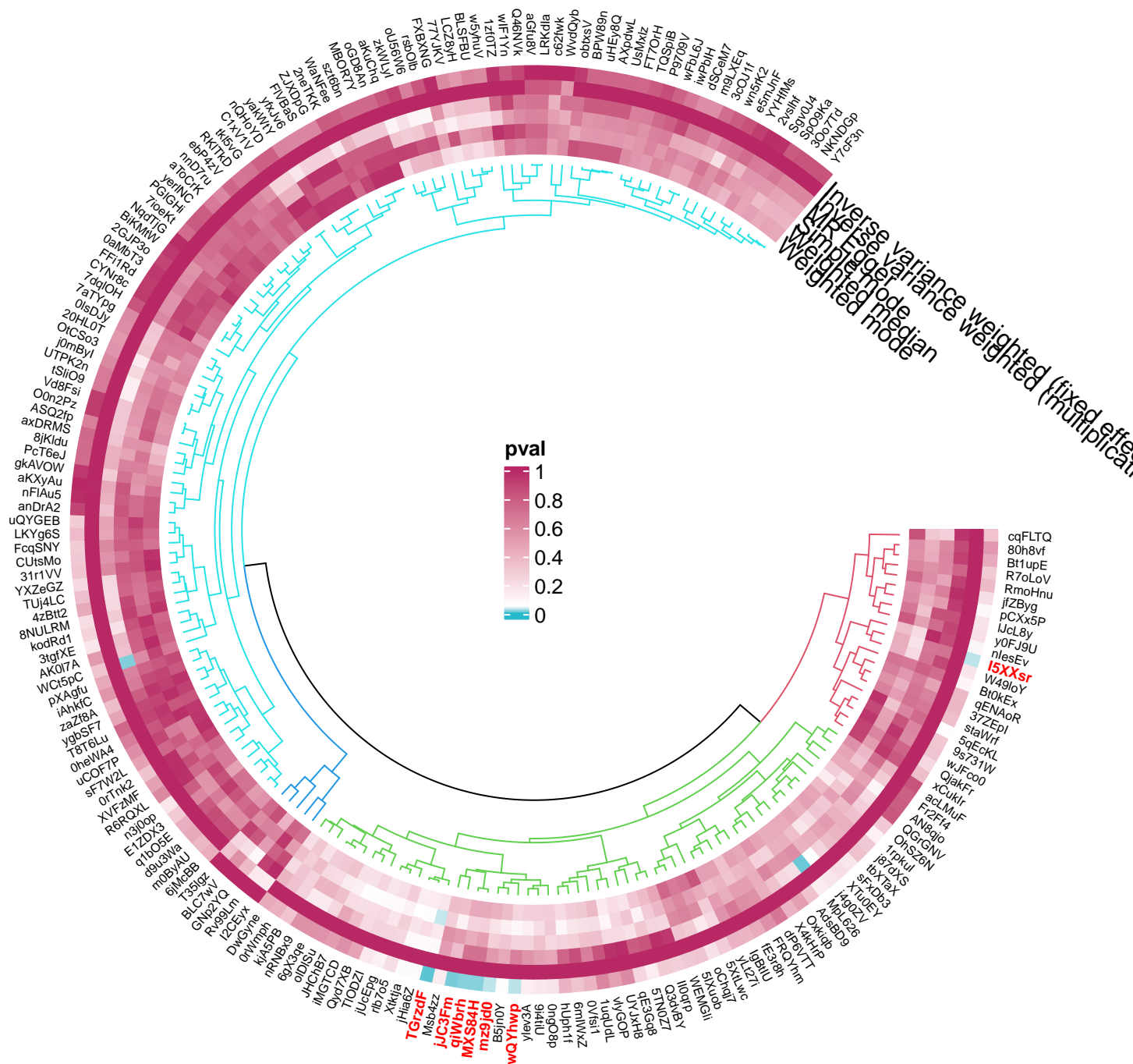

Supplement: Supplementary file 1 [file DataSheet1.zip › Annex 1 _Data/MR results/Hepatic failure, not elsewhere classified/Hepatic failure, not elsewhere classified-figures/Hepatic failure, not elsewhere classified-circos.pdf]

# MR Forest Plot

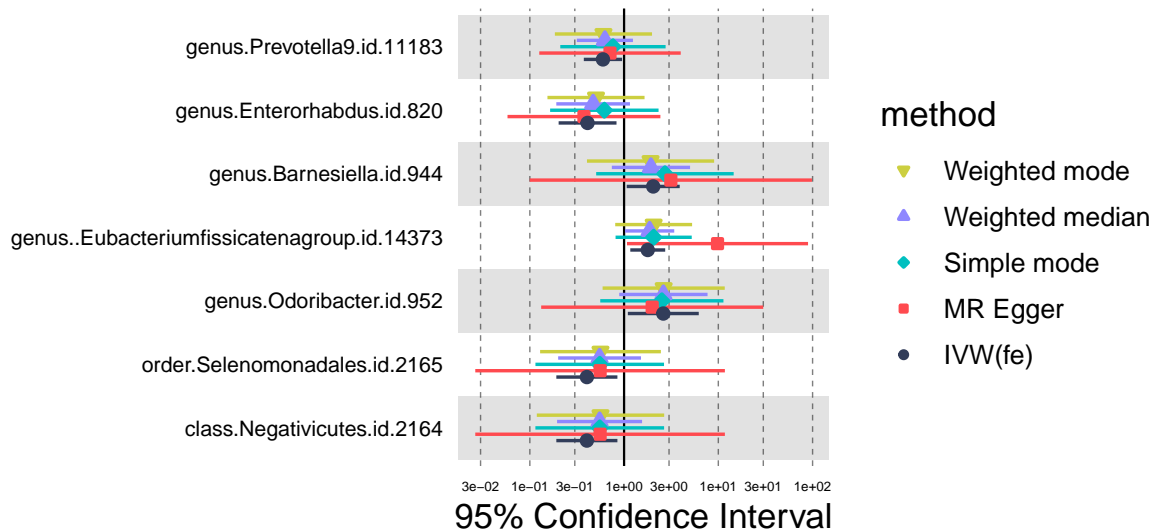

Supplement: Supplementary file 1 [file DataSheet1.zip › Annex 1 _Data/MR results/Hepatic failure, not elsewhere classified/Hepatic failure, not elsewhere classified-figures/Hepatic failure, not elsewhere classified-Forest plot.pdf]

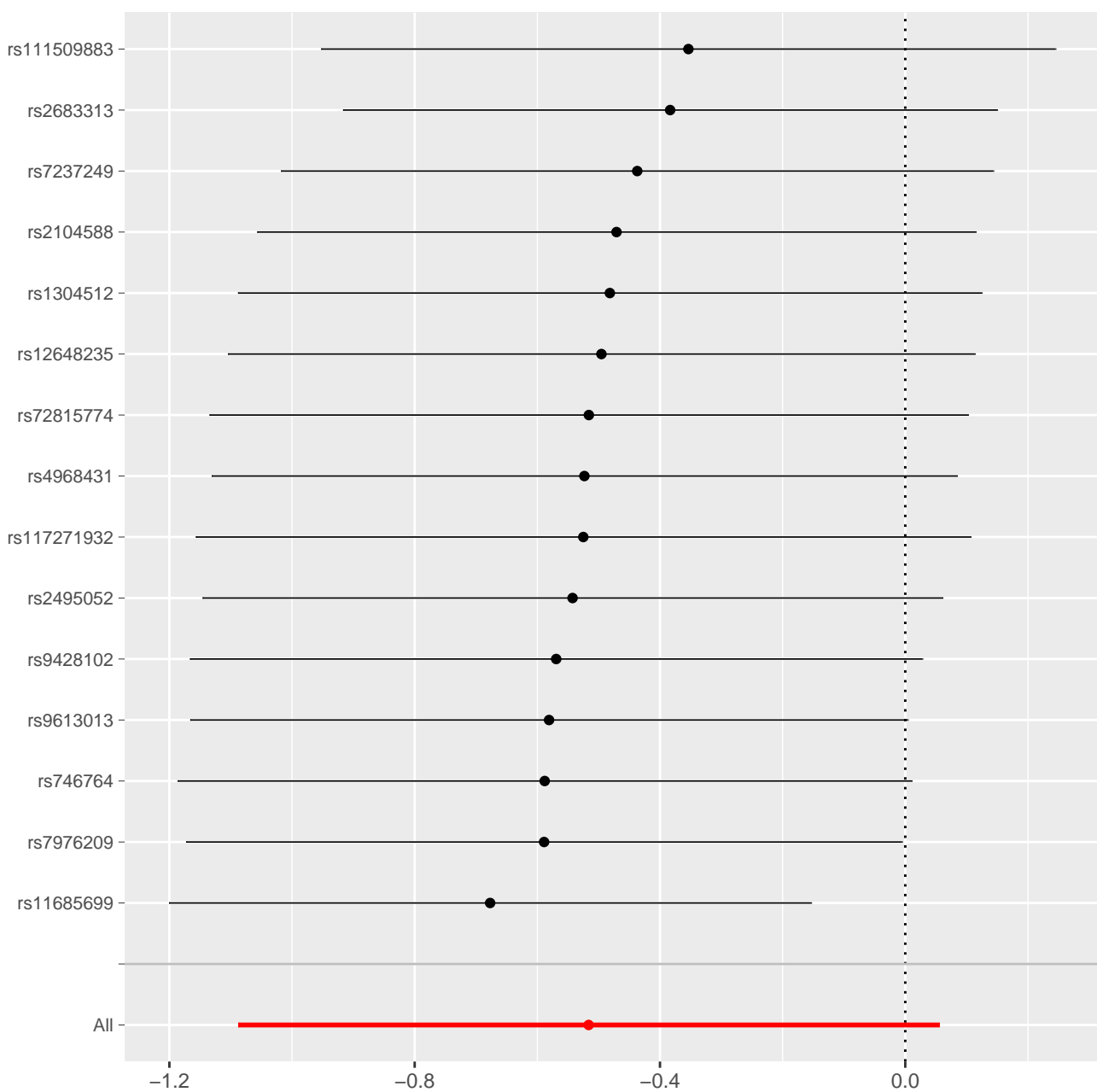

Supplement: Supplementary file 1 [file DataSheet1.zip › Annex 1 _Data/MR results/Hepatic failure, not elsewhere classified/Hepatic failure, not elsewhere classified-figures/LeaveOne_finn-b-K11_HEPFAIL_class.Actinobacteria.id.419.pdf]

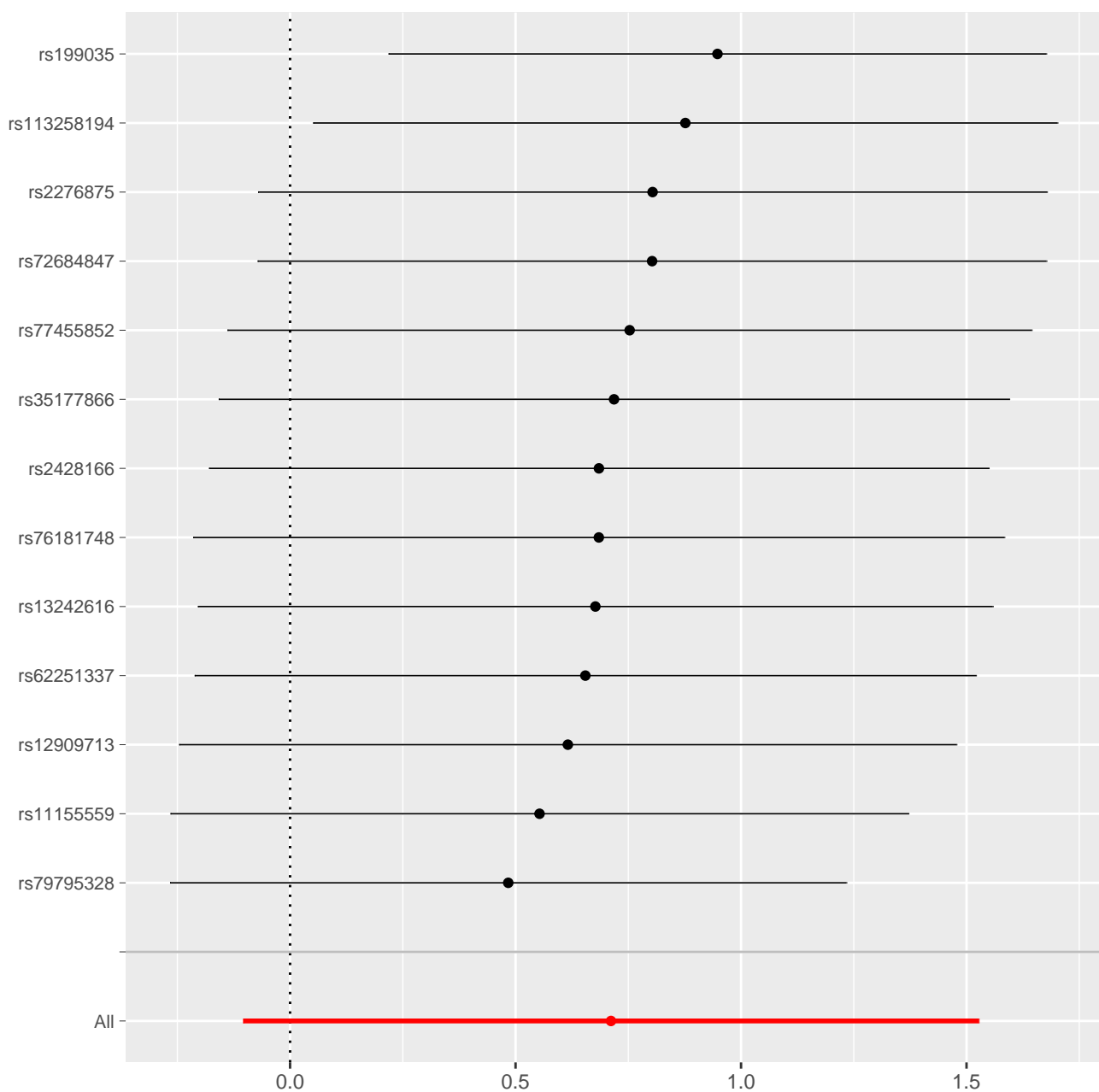

Supplement: Supplementary file 1 [file DataSheet1.zip › Annex 1 _Data/MR results/Hepatic failure, not elsewhere classified/Hepatic failure, not elsewhere classified-figures/LeaveOne_finn-b-K11_HEPFAIL_class.Bacteroidia.id.912.pdf]

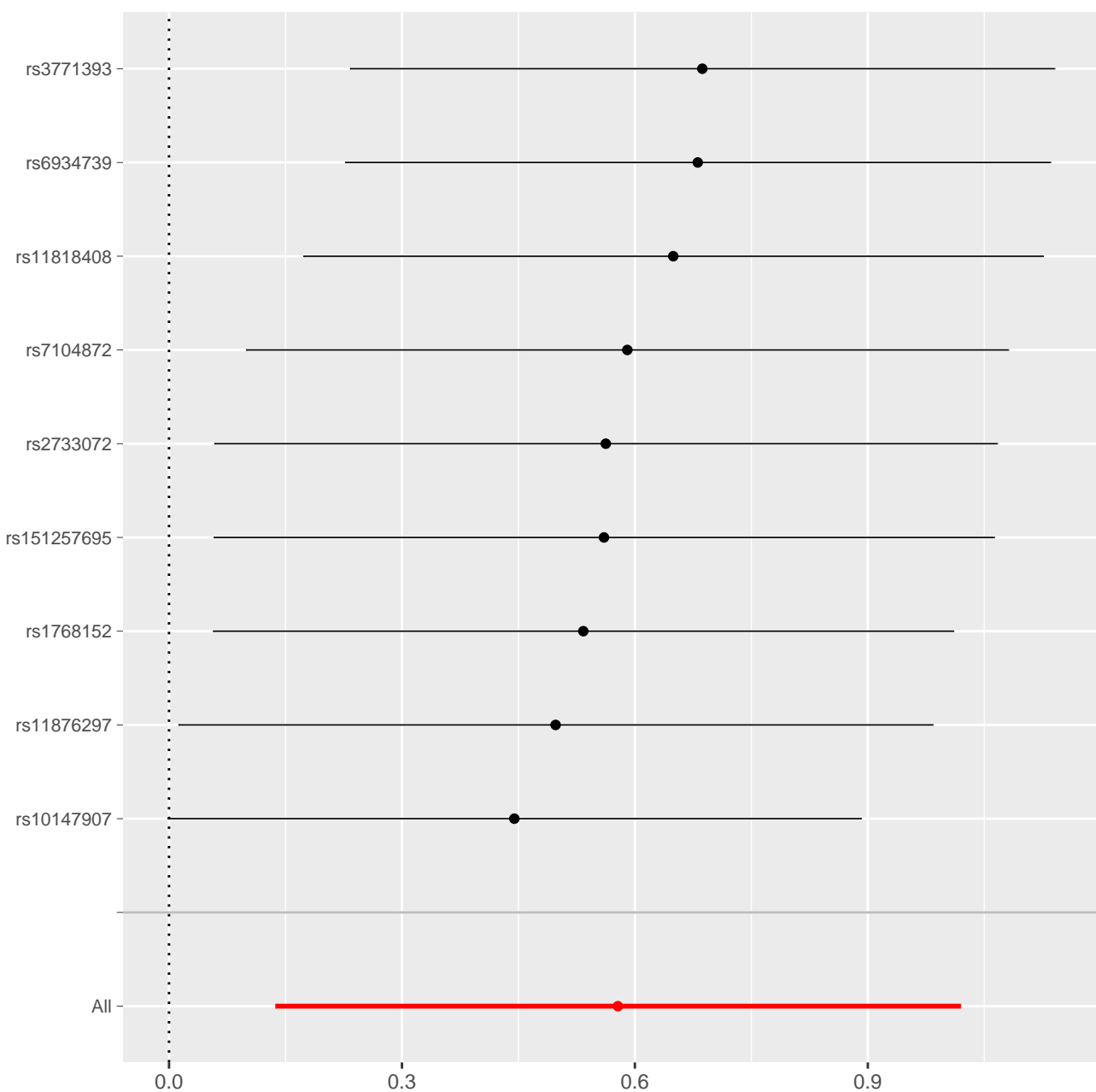

Supplement: Supplementary file 1 [file DataSheet1.zip › Annex 1 _Data/MR results/Hepatic failure, not elsewhere classified/Hepatic failure, not elsewhere classified-figures/LeaveOne_finn-b-K11_HEPFAIL_class.Clostridia.id.1859.pdf]

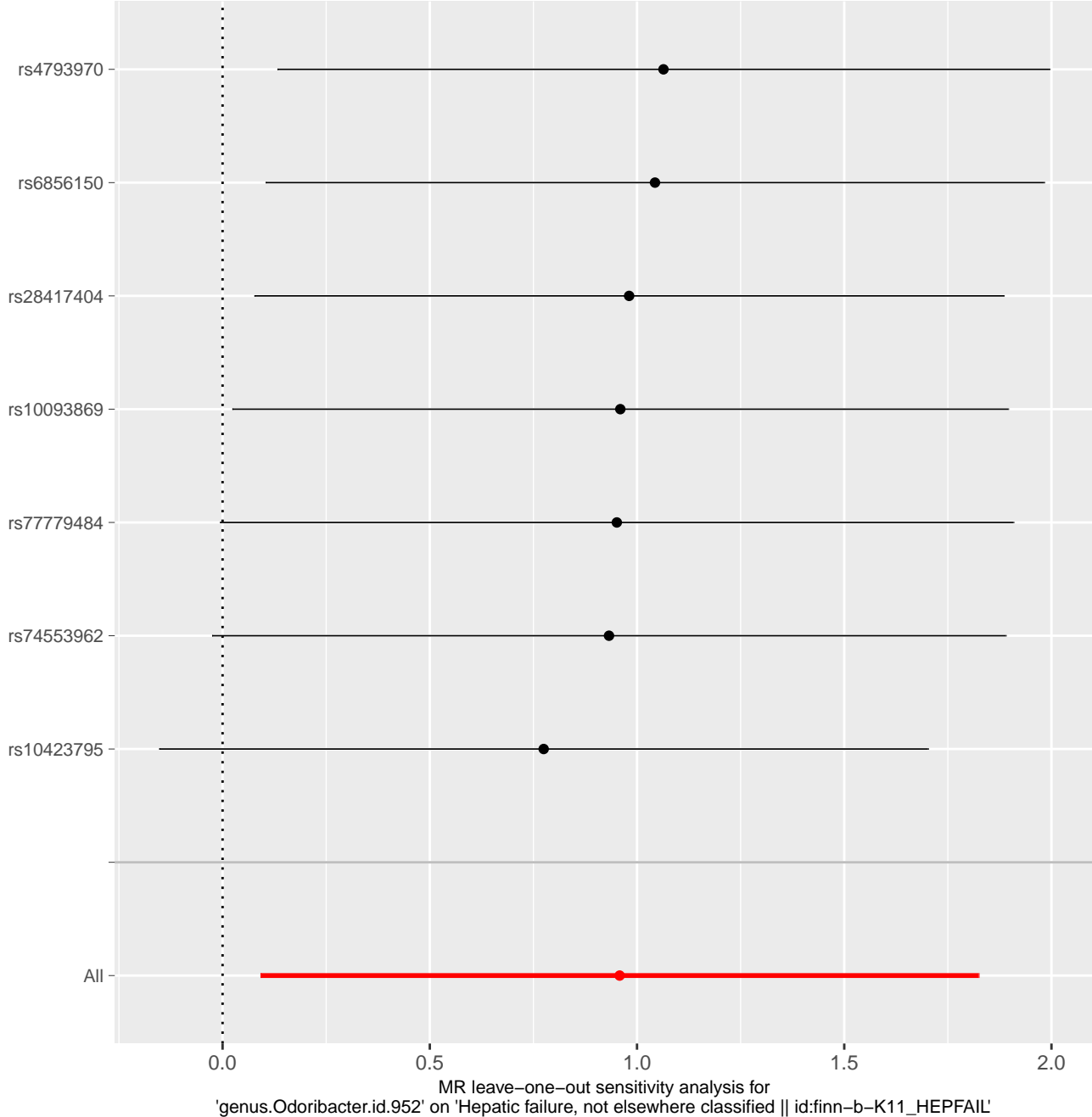

Supplement: Supplementary file 1 [file DataSheet1.zip › Annex 1 _Data/MR results/Hepatic failure, not elsewhere classified/Hepatic failure, not elsewhere classified-figures/LeaveOne_finn-b-K11_HEPFAIL_class.Coriobacteriia.id.809.pdf]

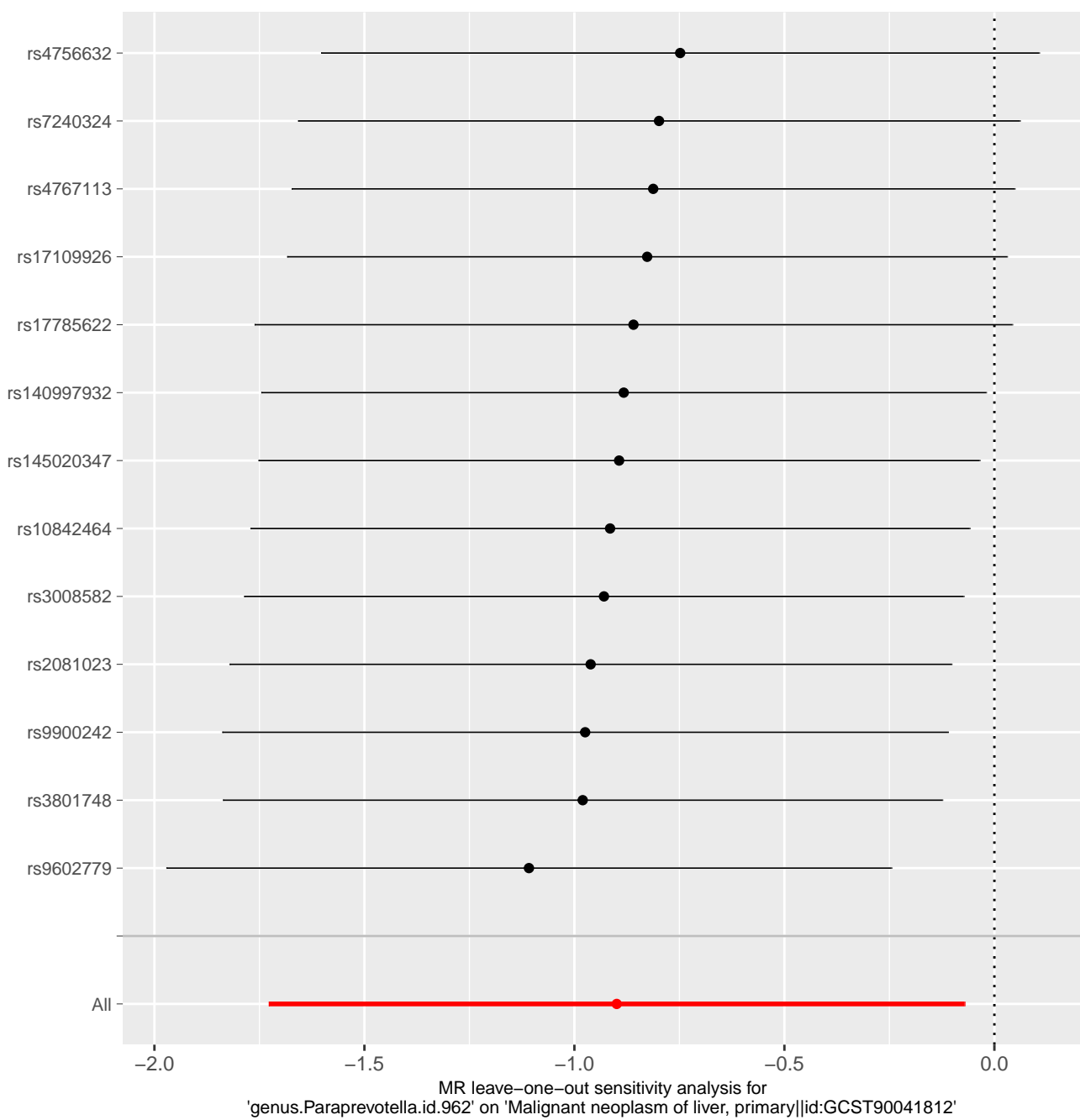

Supplement: Supplementary file 1 [file DataSheet1.zip › Annex 1 _Data/MR results/Malignant neoplasm of liver, primary/Malignant neoplasm of liver, primary-figures/LeaveOne_GCST90041812_class.Bacilli.id.1673.pdf]

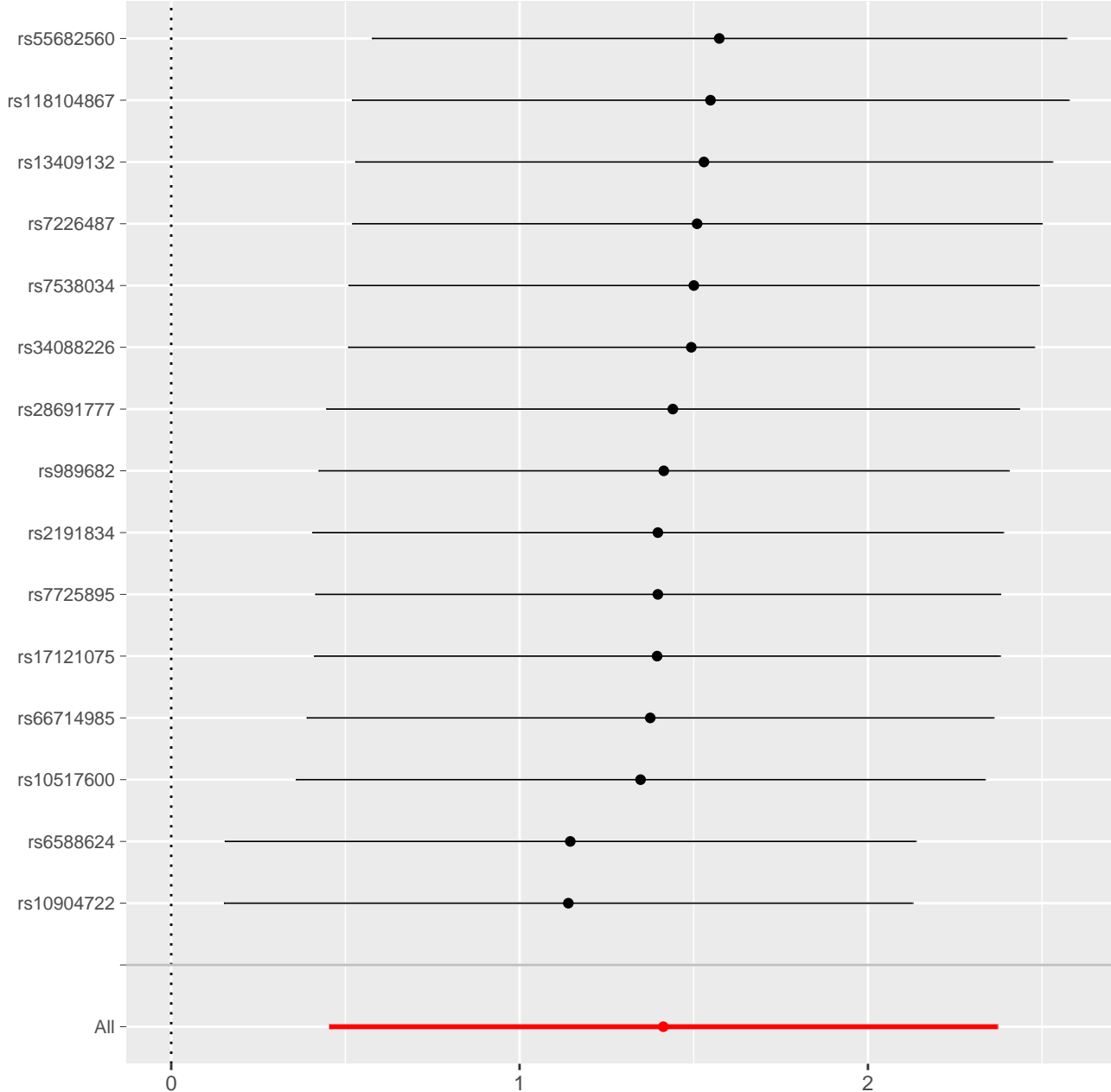

Supplement: Supplementary file 1 [file DataSheet1.zip › Annex 1 _Data/MR results/Malignant neoplasm of liver, primary/Malignant neoplasm of liver, primary-figures/LeaveOne_GCST90041812_class.Bacteroidia.id.912.pdf]

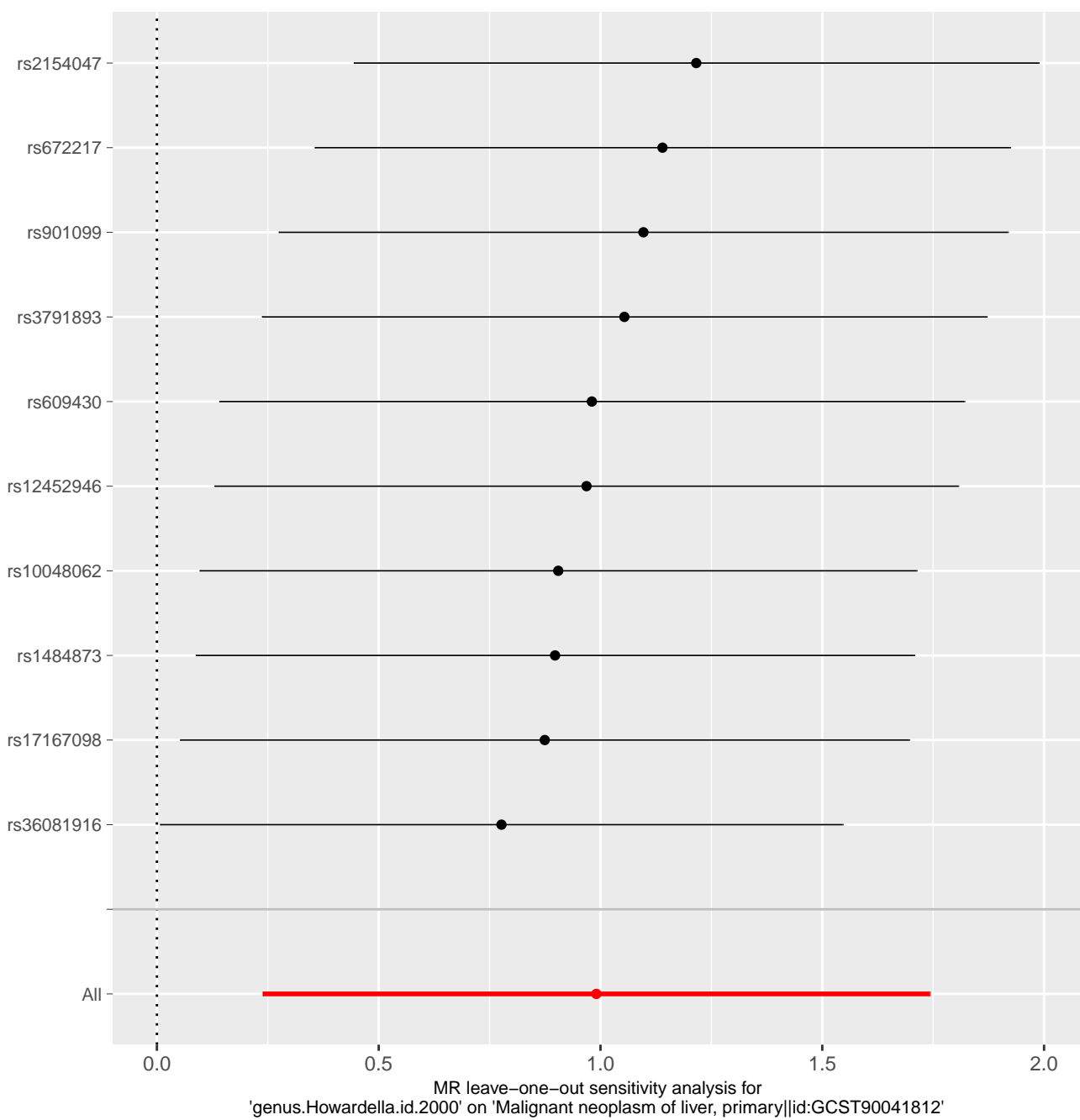

Supplement: Supplementary file 1 [file DataSheet1.zip › Annex 1 _Data/MR results/Malignant neoplasm of liver, primary/Malignant neoplasm of liver, primary-figures/LeaveOne_GCST90041812_class.Clostridia.id.1859.pdf]

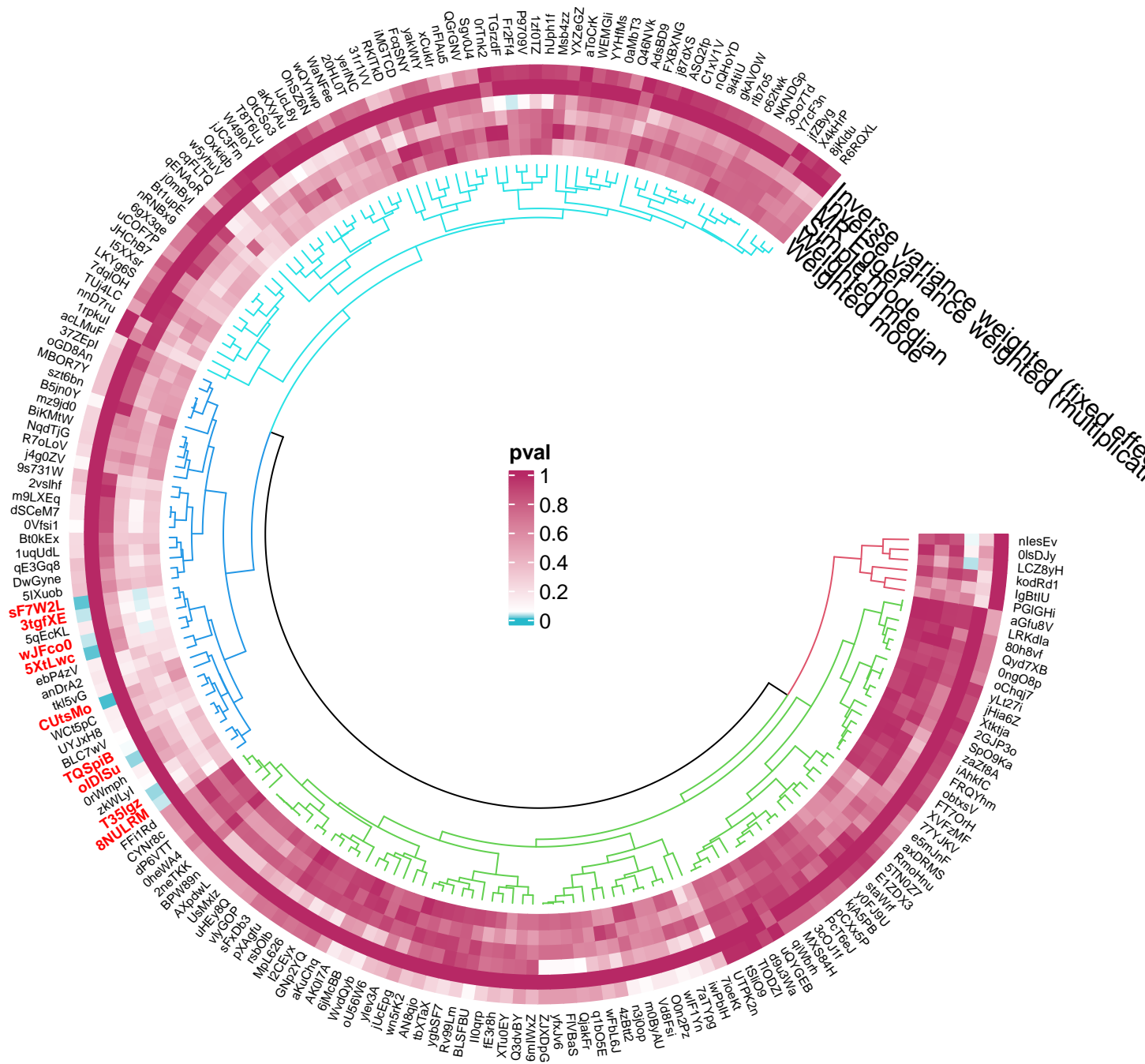

Supplement: Supplementary file 1 [file DataSheet1.zip › Annex 1 _Data/MR results/Malignant neoplasm of liver, primary/Malignant neoplasm of liver, primary-figures/Malignant neoplasm of liver, primary-circos .pdf]

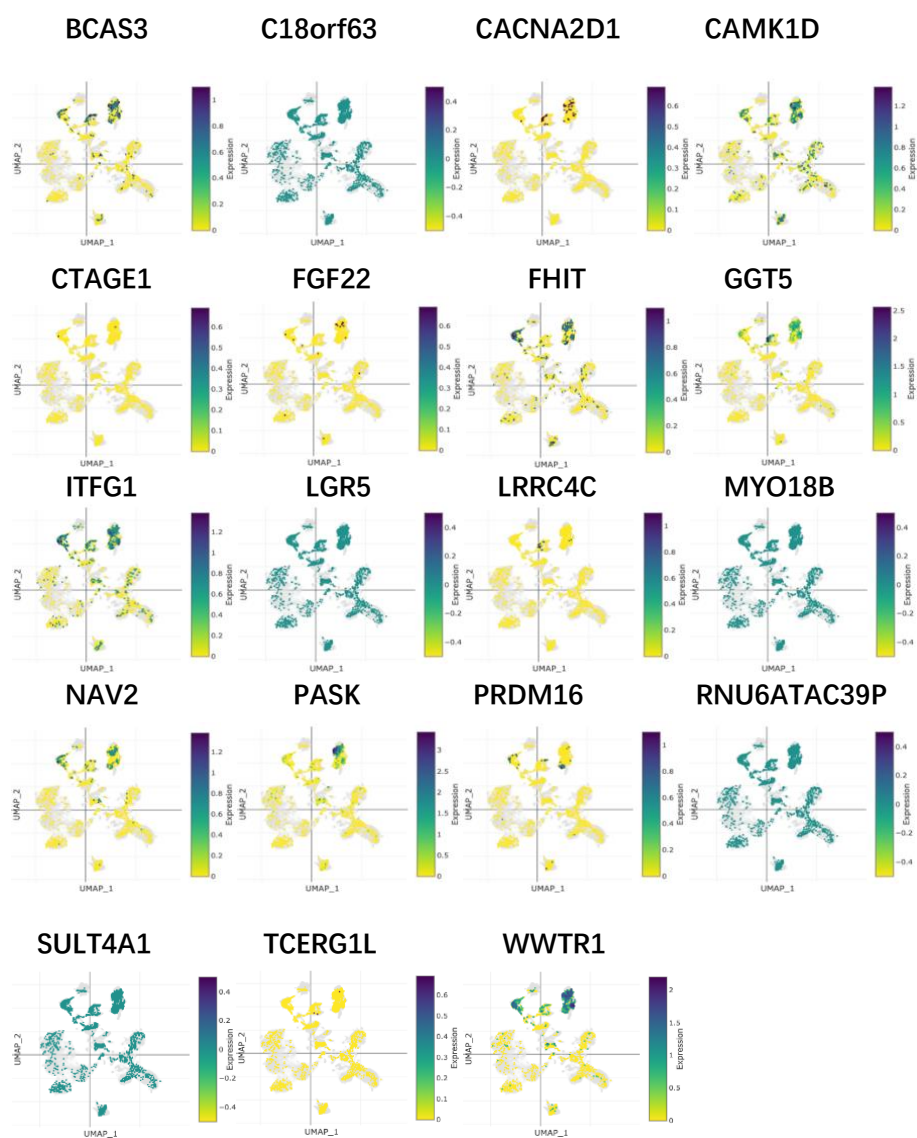

Supplement: Supplementary file 1 [file DataSheet1.zip › Annex 1 _Data/Single-cell results/alcoholic cirrhosis.pdf]

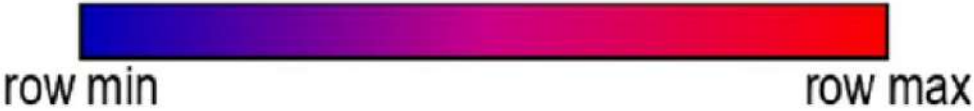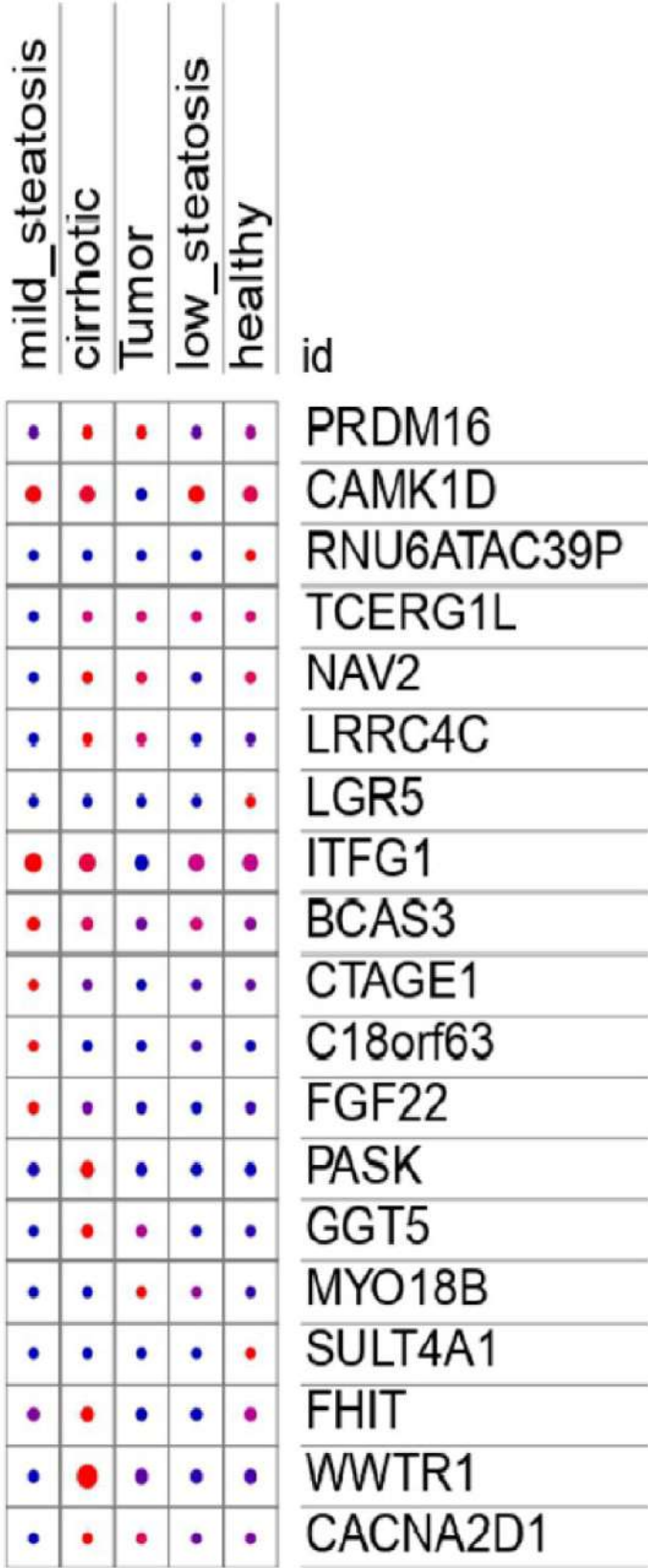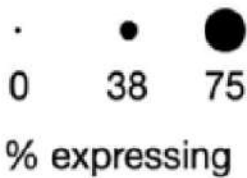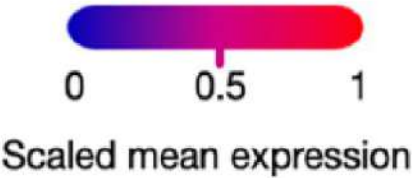

Supplement: Supplementary file 1 [file DataSheet1.zip › Annex 1 _Data/Single-cell results/Gene expression in different liver states.pdf]

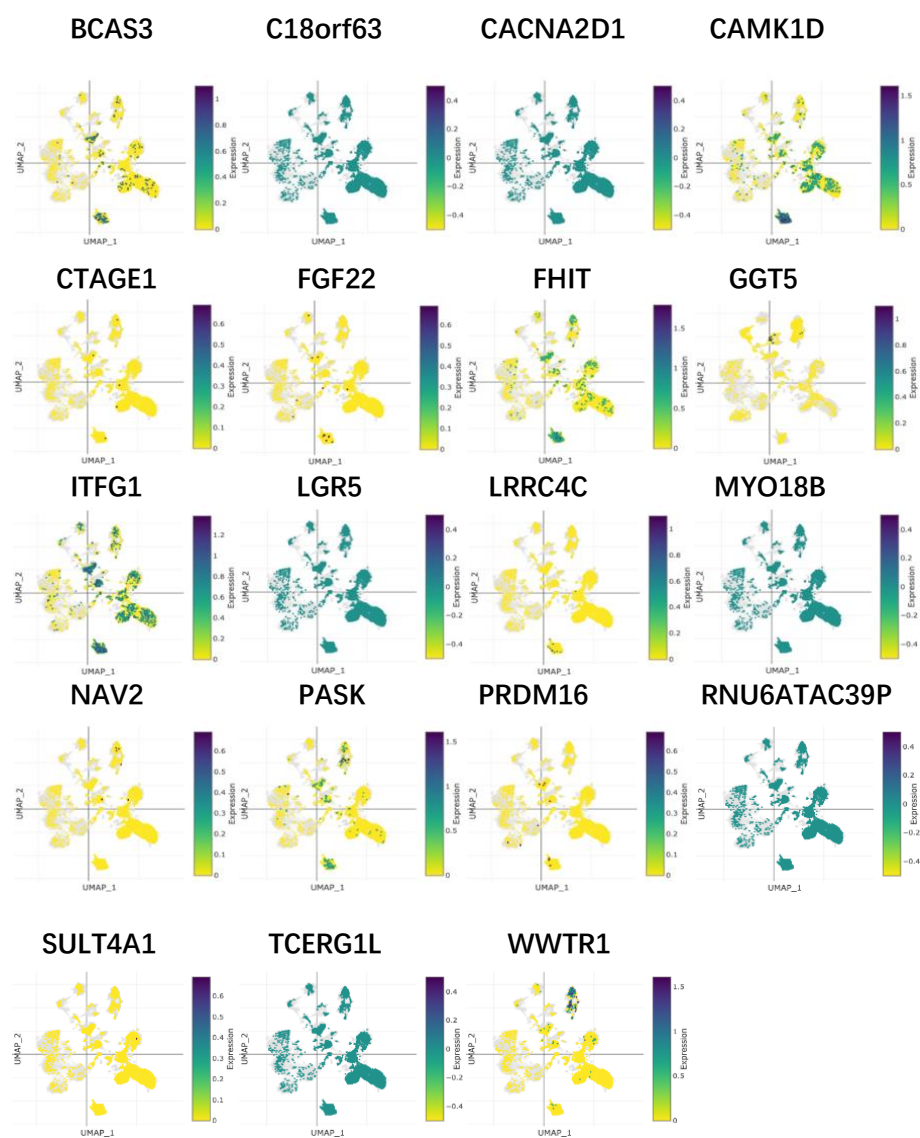

Supplement: Supplementary file 1 [file DataSheet1.zip › Annex 1 _Data/Single-cell results/health .pdf]
